# Revisiting Hydrogen Sorption–Desorption in Natural Rocks

**DOI:** 10.1021/acs.iecr.5c02081

**Published:** 2026-02-03

**Authors:** Mohammad Masoudi, Ariel G. Meyra, Mohammad Nooraiepour, Mohaddeseh Mousavi Nezhad, Aliakbar Hassanpouryouzband, Helge Hellevang

**Affiliations:** † Applied Geoscience Department, 275243 SINTEF Industry, 7465 Trondheim, Norway; ‡ Department of Geosciences, 87361 University of Oslo, P.O. Box 1047, Blindern, 0316 Oslo, Norway; § Instituto de Física de Líquidos y Sistemas Bilógicos, Facultad de Ciencias Exactas-UNLP-CONICET, La Plata 1900, Argentina; ∥ Centro de Investigación en Mecánica Experimental y Computacional, Berisso 1923, Argentina; ⊥ Department of Civil and Environmental Engineering, University of Liverpool, Liverpool L69 3GH, U.K.; # School of Geosciences, Grant Institute, University of Edinburgh, West Main Road, Edinburgh EH9 3FE, U.K.

## Abstract

Hydrogen sorption and desorption in natural rocks are increasingly referenced across subsurface energy and environmental applications, including underground hydrogen storage, natural hydrogen exploration, geological hydrogen generation, and radioactive waste containment. However, the extent to which these physical interactions influence hydrogen behavior in geological materials remains poorly understood. This review examines current experimental and theoretical studies (atomistic simulation and isotherm modeling) of hydrogen sorption and desorption in natural rocks. We evaluated reported sorption capacities and their variability across different lithologies alongside the influencing parameters and the occurrence of hysteresis. Additionally, we modeled all available data using multiple isotherm models to identify the best-fitting formulations. By synthesizing results across diverse methods and geological settings, we identify where physical sorption–desorption is likely to matter, where it is negligible, and what this means for understanding hydrogen transport and retention in the subsurface. Additionally, we provided practical implications of adsorption–desorption, identified critical data gaps, and proposed future research directions to advance the understanding of hydrogen behavior in geological formations.

## Graphical abstract

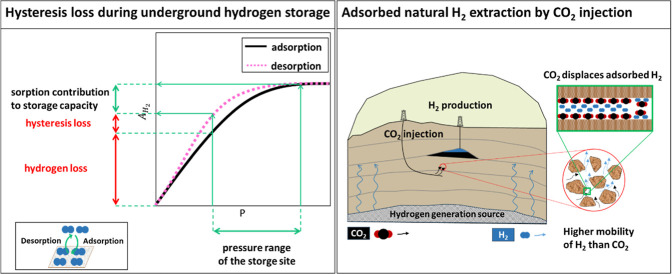

## 1 Introduction

Hydrogen has emerged as a central component in strategies for low-carbon energy systems. While much of the focus has been on hydrogen production, transport, and conversion, there is growing recognition of the role that the subsurface will play in enabling large-scale deployment. Applications such as underground hydrogen storage (UHS), natural hydrogen exploration, hydrogen farming, in situ hydrogen generation, and radioactive waste disposal all involve interactions between hydrogen and geological materials.^1–3^ In each case, understanding how hydrogen is retained, transported, or released within rock formations is essential for assessing system performance, integrity, and long-term behavior.

One of the mechanisms that govern hydrogen behavior in the subsurface is sorption, which is its temporary retention on mineral surfaces or within pore networks. Desorption, the reverse process, determines how readily hydrogen can be recovered or mobilized. These interactions are frequently cited as being relevant to storage efficiency and leakage risk. Despite this, the extent to which sorption and desorption processes affect hydrogen dynamics in natural porous media remains uncertain. Reported data are highly variable, often difficult to compare, and are influenced by experimental design, mineralogy, and environmental conditions.

In this work, we critically examine the current understanding of hydrogen sorption and desorption in natural rocks, including clays, shales, coals, and other common subsurface materials. We assess the experimental methods, theoretical models, and material-specific behaviors that underpin the existing interpretations. Particular attention is given to identifying gaps in data, challenges in measurement, and the relevance of sorption processes to emerging subsurface technologies. By clarifying what is known, where uncertainties remain, and what research is needed, we seek to inform future research directions and provide a foundation for evaluating the role of sorption and desorption in hydrogen-based systems.

### 1.1 Paper Structure: How to Read the Paper

This paper is organized to guide readers from foundational concepts through detailed experimental and theoretical studies, culminating in practical implications for subsurface hydrogen systems. Section 1 introduces the importance of hydrogen sorption–desorption across energy and waste storage applications and summarizes the main measurement techniques (volumetric and gravimetric).

Section 2 presents the fundamental principles of hydrogen physisorption, including molecular interactions, thermodynamics, and classic adsorption isotherm models, drawing distinctions between well-established theory and novel contributions in natural porous media.

Section 3 offers a chronological, lithology-based review of experimental studies of high-pressure, high-temperature H_2_ adsorption–desorption in natural porous materials. Each material group is concluded with a summary table of the reported adsorption data and experimental conditions.

Section 4 surveys theoretical research methodologies, including atomistic simulations (detailing molecular-scale insights into hydrogen interactions with different surfaces) and adsorption isotherm modeling (listing the available modeling parameters and comparing different formulations against experimental data).

Section 5 provides a critical synthesis of experimental data and analyzes influencing factors across thermodynamic (pressure, temperature, and hydration), textural (surface area, pore volumes, and pore size), and structural/mineralogical parameters, quantifying their relative impacts on hydrogen uptake in each rock type.

Section 6 discusses practical implications for UHS, natural hydrogen exploration, and radioactive waste containment, highlighting where sorption–desorption processes materially affect storage capacity, leakage risk, and recovery efficiency.

Section 7 identifies critical data gaps and proposes future research directions to advance our understanding of hydrogen behavior in geological formations.

### 1.2 Importance of Hydrogen Sorption–Desorption in the Hydrogen Energy Field

Intermittency and seasonal mismatch of supply and demand pose significant challenges for clean energy resources, such as solar, wind, and wave energy.^3–5^ Addressing these challenges requires the development of effective energy storage solutions to ensure a stable energy supply chain from renewable energy sources. Energy carriers and batteries are key components in energy storage and withdrawal.^6^ Despite recent advances in battery technology, their energy storage capacity remains limited. In this context, UHS has emerged as a promising alternative.^2,3,7–9^

Hydrogen stands out as a versatile energy carrier due to its exceptionally high energy content per unit mass and zero emissions upon use. Underground repositories such as salt caverns, aquifers, depleted hydrocarbon reservoirs, and rock caverns can serve as potential “batteries” for storing hydrogen/energy.^2–4,10–13^ These reservoirs have the capacity to store vast amounts of hydrogen, making them suitable candidates for large-scale energy storage applications.

Within the scope of UHS, H_2_ adsorption and desorption have several implications. It serves as a H_2_ storage mechanism, especially in shales and coal beds.^14–16^ Moreover, simulations indicated that the sorption indices of kerogen and clay minerals are essential factors for estimating H_2_ storage capacity in shale reservoirs.^17^ H_2_ sorption plays a crucial role in determining the potential for H_2_ losses through geological barriers and top sealing layers (i.e., both caprock and overburden layers).^18–24^ These geological sealing sequences consist of diverse clay mineral assemblages that define the pore space characteristics, transmissivity and diffusivity, and sealing efficiency.^25–28^

Furthermore, coal exhibits a lower capacity for sorbing H_2_ relative to those of CO_2_ and CH_4_ and presents a markedly higher diffusion rate for H_2_. Recent studies have introduced an innovative approach that involves injecting a mixture of CO_2_ and H_2_, derived from steam methane reforming (SMR) for H_2_ production (gray H_2_), into coal seams. This method aims to achieve two objectives simultaneously: separate H_2_ from the gas mix and sequester CO_2_ within the coalbed.^29,30^

Additionally, sorption–desorption dynamics can be used as a storage mechanism for surface hydrogen storage in hydrogen storage materials (HSM).^31–33^ However, the focus of this study is not on solid-state hydrogen storage systems. The storage capacity of natural porous materials is generally lower than that of advanced synthetic or modified-natural materials such as metal–organic frameworks (MOFs), activated carbons, and carbon nanotubes. It is also highly dependent on temperature and pressure, with reduced efficiency at room temperature.^32^ Additionally, this is a short-scale storage method.^14^ More on this type of storage method can be found in many review papers in this field.^31,33–41^

In the context of hydrogen exploration, hydrogen migration and mobility through the Earth’s crust are significantly impacted by its sorption onto surrounding rocks, providing valuable insights for the prospecting of natural hydrogen sites.^5,7,42,43^

It should be noted that although hydrogen is promoted as a crucial element for decarbonization and the advancement of sustainable energy practices, the stark reality is that low-emission hydrogen production constitutes less than 1% of the global total. In 2022, most of the global hydrogen production was derived from natural gas (62%) and coal (21%). Methods like SMR and coal gasification for hydrogen production cause substantial CO_2_ emissions, ranging from 22 to 26 kg CO_2_ equivalent per kg of hydrogen (CO_2_-eq/kg H_2_) for SMR and 10–13 kg CO_2_-eq/kg H_2_ for coal gasification, thus offering no climate benefit.^44^ Consequently, it is important to develop more advanced and environmentally friendly hydrogen production and exploration methods.^5^

### 1.3 Importance of Hydrogen Sorption–Desorption in the Waste Storage Field

Beyond energy applications, hydrogen sorption plays a key role in containing hazardous and radioactive waste. The degradation of waste materials, such as bitumen and radioactive ash, releases hydrogen gas. Additionally, hydrogen can be generated through corrosion of metallic containment vessels.

If gas generation exceeds the diffusive flux, free hydrogen gas can migrate to the clay-rich caprock. High gas pressure might even lead to fissures and fractures, compromising the containment system. Therefore, understanding the interactions between hydrogen and rock becomes critical in these contexts. This knowledge is essential for assessing the long-term safety and integrity of containment systems, ensuring they effectively isolate hazardous materials, thus safeguarding environmental and public health.^45–47^

Some examples of clay-rich formations being explored as potential sites for deep underground radioactive waste disposal facilities include Callovo-Oxfordian (COx) claystone in France,^45,47,48^ Boom Clay and Ypresian Clay in Belgium,^49–51^ Opalinus Clay in Switzerland,^52^ Boom Clay in the Netherlands,^51,53^ Boda Claystone in Hungary,^54^ and Eleana argillite Conasauga shale (USA).^55^

### 1.4 Hydrogen Sorption Measurement Techniques

Understanding the hydrogen sorption properties of materials requires accurate and reproducible measurement methods. Two primary experimental approaches are commonly employed: volumetric (manometric or Sievert-type) and gravimetric techniques. Both methods determine hydrogen uptake by exposing materials to controlled hydrogen pressures and temperatures, but they employ different detection principles.^56–59^

#### 1.4.1 Volumetric (Manometric) Measurements

Volumetric measurements, also known as manometric measurements (manometry) or the Sieverts technique, quantify hydrogen sorption by tracking pressure variations within calibrated volumes. In this approach, a sample chamber is connected to a reference reservoir of known volume through an isolation valve. Upon opening the valve, hydrogen equilibrates between the two volumes, and any deviation in pressure from the expected value, calculated using the equation of state, corresponds to the amount of hydrogen taken up by the material. Conversely, during desorption, the quantity of hydrogen released is determined from the measured pressure increase after the evacuation of hydrogen from the gas phase. Modern volumetric systems improve accuracy through the use of multiple high-precision pressure transducers and advanced thermal management to ensure isothermal conditions throughout the measurement.^57,58,60^

#### 1.4.2 Gravimetric Measurements

Gravimetric techniques directly measure changes in the exposed sample’s apparent mass using an ultrasensitive microbalance. Typically, a small specimen (on the gram or subgram scale) is suspended within a sealed chamber, evacuated, and stabilized at the desired temperature. Hydrogen is then introduced in incremental pressure steps, and the balance records the corresponding mass change once equilibrium is established at each point, thereby generating the sorption isotherm. The technique provides direct determination of sorption kinetics and equilibrium properties, with automatic correction for buoyancy effects through simultaneous density measurements of the gas phase.^56–58^

## 2 Fundamental Principles of Hydrogen Physisorption

Understanding hydrogen sorption behavior requires a knowledge of fundamental adsorption science principles that govern molecular interactions at solid–gas interfaces. This subsection briefly establishes the theoretical framework underlying the experimental observations discussed throughout this review, preventing repetitive explanations and clearly distinguishing established science from novel research findings. More details about the principles of adsorption by porous materials can be found in different textbooks.^61,62^ A more detailed analysis of the hydrogen sorption characteristics and mechanisms in clay minerals, shale, and coal can be found elsewhere.^63^

### 2.1 Molecular Interactions and Adsorption Affinity

Physisorption arises from broadly applicable low-specificity interactions between an adsorbate and its substrate. The overall adsorption energy depends primarily on the particular adsorbent–adsorbate combination. In every physisorption system, three “non-specific” forces contribute to this energy: Dispersion (London) attractions, short-range repulsive forces, and polarization interactions. Because these three terms operate regardless of molecular polarity or surface charge, they can be grouped as universal contributions to physisorption. When the adsorbate possesses a permanent dipole or quadrupole—and the surface is ionic or polar—additional, “specific” electrostatic interactions arise. These include field-dipole and field-gradient (quadrupole) energies. These electrostatic terms apply only to certain adsorbate–adsorbent pairs and are therefore classified as specific interaction energies.^61^ Some references have divided these forces as van der Waals forces (dispersion–repulsion) and electrostatic interactions comprising polarization, dipole, and quadrupole interactions.^62^ Due to its nonpolar nature and minimal quadrupole moment, hydrogen physisorption is governed by van der Waals forces and weak electrostatic attractions. This is why hydrogen storage under ambient conditions requires materials with extremely high surface areas and optimized pore structures to achieve practical storage densities.

For gas molecules relevant to H_2_-related applications mentioned in the previous section, the polarizability volumes show a clear hierarchy: H_2_ (0.819 Å^3^) ≪ CH_4_ (2.89 Å^3^) < CO_2_ (2.93 Å^3^).^64^ The quadrupole moment magnitudes are H_2_ (0.651 B), CH_4_ (0 B), and CO_2_ (−4.3 B), where B is Buckingham (equivalent to 10^–26^ esu cm^2^).^65^ This fundamental characteristic explains why H_2_ physisorption capacities are generally lower than those observed for polar gases like CO_2_, which can engage in additional specific electrostatic interactions with appropriate adsorbent sites. The combination of these properties establishes a universal adsorption hierarchy: CO_2_ > CH_4_ > H_2_. This trend appears consistently across diverse materials.

### 2.2 Thermodynamic Principles of Physisorption

All gas physisorption processes are exothermic—the differential enthalpy has negative values (Δ*H* < 0)—meaning heat is released when gas molecules adsorb onto solid surfaces.^61^ According to Le Châtelier’s principle, raising the temperature of an exothermic process drives the equilibrium toward the reverse (endothermic) direction. Therefore, higher temperatures favor desorption over adsorption, thereby reducing gas uptake.

Pressure dependence at low pressures follows Henry’s law, where the adsorbed H_2_ amount is linearly proportional to gas pressure.

### 2.3 Adsorption Isotherm Models and Material Heterogeneity

Adsorption isotherm theories have evolved to capture the variability found in real materials that seldom exhibit perfectly uniform surfaces. The widely used Langmuir isotherm assumes homogeneous surfaces with a uniform binding energy and monolayer coverage. While useful for idealized systems, this model often fails for real materials due to several limitations, such as surface heterogeneity, multilayer adsorption, and adsorbate–adsorbate interactions. The Langmuir equation is consistent with the general shape of a Type I isotherm of the IUPAC classification.^61,66^ For more heterogeneous and complex systems, various theoretical and empirical models have been developed over the past century. The heterogeneity parameter in these models (especially the empirical ones) accounts for the deviation from ideal Langmuir behavior. In Section 5 of this paper, we analyze published experimental equilibrium data by using a selection of commonly employed adsorption isotherm models. For more comprehensive coverage of the application of empirical isotherms, readers are referred to Chapter Five of Rouquerol et al.^61^

## 3 Experimental Studies of Hydrogen Adsorption–Desorption in Natural Porous Materials

In this section, we present a comprehensive overview of the current literature on experimental studies of H_2_ adsorption and desorption in natural porous materials. These materials are categorized primarily based on the availability and volume of published data, rather than strict lithological distinctions: clays, shales, coals, and a diverse group that includes sandstone, mudstone, diatomite, and diatomaceous earth. This grouping reflects the availability of experimental data, lithology, and practical applications (e.g., coalbed methane systems vs shale caprocks).

Each subsection provides a chronological enumeration of research from each group. We have aimed to highlight the primary contributions of each work to the field, while deliberately omitting results that are already well established, as outlined in Section 2. Naturally, different readers may derive distinct perspectives from the same body of literature; the interpretations presented here reflect our own assessment of these studies. Additionally, a more critical synthesis and analysis of the influencing factors based on the experimental data are provided in Section 5.

At the end of each subsection, we provide a summary table detailing the H_2_ sorption data and characteristics of the samples studied. Here are a few important notes regarding these tables:

- Each table presents the most significant or frequently reported parameters for each material group.
- The primary focus is on high-pressure, high-temperature data available in the literature.
- Not all parameters are reported in every study.
- The reported H_2_ sorption values correspond to the plateau, or the maximum pressure indicated in the table.
- The treatment of the samples is included in each table to highlight that most studies have conducted their experiments on dry powders.
- The experimental setup is also documented, as the sorption process can be influenced by the specific setup and procedures used.

We have also included example H_2_ adsorption isotherms at the end of each section to illustrate the appearance of the data.

### 3.1 Clay

In this section, we review recent studies on H_2_ sorption–desorption in clay-rich materials. Relevant high-pressure and high-temperature data for these studies are summarized in Table 1. Figure 1 illustrates example H_2_ adsorption isotherms for some clay minerals reviewed in this section.

**Table 1:** Summary of H_2_ Adsorption Studies on Clays

refs	sample	BET surface area (m^2^/g)	pore volumes (cm^3^/g)	micropore volumes (cm^3^/g)	average pore diameter (nm)	*T* (°C)	pressure range (bar)	H_2_ adsorption [* at plateau] (mmol/g)	sample treatment	experimental setup
Didier et al. (2012)^47^	Na–montmorillonites (with 0% structural Fe(III))	91.5 ± 9.1				90	0.42	0.55	dried in an oven at 120 °C for 48 h	volumetric (custom experimental setup with gas chromatography). The samples were exposed to a mixture of Ar/H_2_ (95:5%) for 30–45 days
						120	0.45	0.35		
	Na–montmorillonites (3.2% Fe)	116.9 ± 11.6				90	0.45	0.35		
						120	0.45	0.3		
	Na–montmorillonites (6.4% Fe)	102.9 ± 10.3				90	0.45	0.4		
						120	0.45	0.35		
	Callovo–Oxfordian clay rock (COX_raw_), eastern France	46.4 ± 4.6				90	0.45	0.25		
						120	0.45	0.2		
	pure clay fraction of COX (COX_pur_)	83.7 ± 8.4				90	0.40	0.3		
						120	0.45	0.25		
Ruiz-García et al. (2013)^67^	Na–montmorillonite (Na–MMT)	16		0.007		25	0–200	0	degassed at 150 °C under vacuum for 4 h and evacuated at 130 °C under vacuum for 4 h	volumetric (Quantachrome iSorbHP1)
	thermally treated carbon–montmorillonite (C/MMT)	4		0.002		25	0–200	*0.35		
	sepiolite	89		0.04		25	0–200	0		
	thermally treated carbon–sepiolite (C/SEP)	166		0.08		25	0–200	*0.70		
						50	0–120	0.53		
						75	0–120	*0.25		
Bardelli et al. (2014)^45^	COX_raw_ (Illite: 17, kaolinite: 2.6, chlorite: 2.0, mixed-layer Illite/smectite *R*_O_: 27, carbonates: 20, quartz: 22, other minerals: <5)	46 ± 5	0.05	0.01		28	4–90	*0.480	low TP: dried (vacuum at 120 °C for 30 days)	volumetric (pressure composition isotherm (PCI) unit, Advanced Materials Corp.)
						90	4–64	*0.63	high TP: degassed at 120 °C for 24 h under vacuum, and the samples were degassed for 1 h at the test temperature before starting the experiment	
	COX_pur_ (clay > 95%)	84 ± 8	0.10	0.02		–253	*p*/*p*_0_ ≤ 0.82	0.850		
						28	4–90	*1.020		
						90	4–90	*1.200		
Mondelli et al. (2015)^68^	Na–montmorillonite with 0% of structural iron	91 ± 9	0.20 ± 0.02	0.02 ± 0.002		90	0–90	*1	vacuum at 120 °C for 24 h	volumetric (PCI units)
						–253	*p*/*p*_0_ ≤ 0.22	0.52		
	Na–montmorillonites (6% Fe)	100 ± 10	0.28 ± 0.03	0.013 ± 0.001		90	0–90	*1		
Ziemiański and Derkowski (2022)^69^	montmorillonite underwent cation exchange for tetramethylammonium and dried at 315 °C **(TMA_Mt_315 °C)**	273.1		0.110		25	0–145	0.516	predried ex situ at 315 °C for 20 h and then additionally dried in situ at 210 °C for 4 h	gravimetric (IsoSorp, Rubotherm Germany)
						50	0–145	0.409		
						70	0–145	0.351		
	Cs_Mt_60 °C	58.8		0.021		25	0–150	0.207	dried for 20 h at a temperature between 40 and 210 °C	
	Cs_Mt_210 °C	71.9		0.025		25	0–148	0.191		
	Mg_Mt_40 °C					25	0–143	0.208		
	Mg_Mt_60 °C	128.1		0.047		25	0–145	0.199		
	Mg_Mt_110 °C	63.2		0.023		25	0–145	0.140		
	Mg_Mt_210 °C	71.5		0.023		25	0–150	0.118		
						50	0–145	0.101		
						70	0–145	0.081		
	Li_Mt_40 °C	-				25	0.144	0.168		
	Li_Mt_60 °C	64.3		0.026		25	0–145	0.175		
	Li_Mt_210 °C	117.6		0.032		25	0–142	0.141		
						50	0–145	0.110		
						70	0–145	0.096		
	Cs_Bd(f)_210 °C	43.8		0.014		25	0–148	0.106		
	well-crystallized, hydrothermal Illite (marked as Zempleni), from Füzérradvány, Hungary, dried at 210 °C (Zempleni_210 °C)	38.5		0.008		25	0–150	0.050	dried at 210 °C under ∼1 Pa vacuum, for 20 h	
	ultrafine-grained, barrel-shaped Illite of lacustrine origin from Le Puy, France, dried at 210 °C (LePUY(f)_210 °C)	165.7		0.043		25	0–150	0.201		
						50	0–150	0.172		
						70	0–150	0.143		
Wolff-Boenisch et al. (2023)^23^	natural pure montmorillonite	18.9	0.089	0.0151	19	–196.15	0–50	0.4	not indicated	volumetric (PCTPro, SETARAM)
						–78.15	0–50	0.32		
						29.85	0–50	0.1		
Wang et al. (2023)^43^	palygorskite	198.99				0	0–183	0.261	not indicated for H_2_	volumetric (BSD-PH high-pressure analyzer)
						25	0–183	0.223	for N_2_: degassed at 150 °C for 8 h	
						45	0–183	0.208		
						65	0–183	0.178		
						75	0–183	0.169		
	sepiolite	229.29				0	0–168	0.916		
						25	0–168	0.809		
						45	0–168	0.72		
						65	0–168	0.669		
						75	0–168	0.532		
	montmorillonite	45.27				0	0–172	0.122		
						25	0–172	0.071		
						45	0–172	0.058		
						65	0–172	0.039		
						75	0–172	0.01		
	chlorite	18.26				0	0–172	0.027		
						25	0–172	0.021		
						45	0–172	0.02		
						65	0–172	0.015		
						75	0–172	0.011		
	kaolinite	16.85				0–75	0–180	0		
	Illite	13.69				0–75	0–180	0		
Ghosh et al. (2023)^72^	acid-treated montmorillonite (MMT_1)	203.25	0.272	0.0228	4.705	40	0–90	0.278 ± 0.048	pretreated at 383 K at a high vacuum for 24 h	volumetric
						–196.15	1.2	0.827		
	acid-treated montmorillonite (MMT-2)	218.08	0.293	0.0233	4.820	40	0–95	0.448 ± 0.071		
						–196.15	1.2	0.893		
	hydrophilic bentonite (BEN-1)	11.29	0.023	0.0187	7.135	40	0–93	0.160 ± 0.026		
						–196.15	1.2	0.076		
	hydrophilic bentonite (BEN-2)	62.35	0.060	0.0049	3.899	40	0–63	0.096 ± 0.022		
						–196.15	1.2	0.300		
Zhang et al. (2024)^73^	sepiolite	149.9377	0.3796	0.0153		0	0–100	0.57465	dried at 373.15 K in an oven for over 10 h. The samples were degassed at 473.15 K under constant vacuum for 6 h in the adsorption cell	volumetric (iSorb HP2, Quantachrome)
						10	0–100	0.54558		
						20	0–100	0.52133		
						30	0–100	0.47653		
						40	0–100	0.42468		
						50	0–100	0.35902		
	montmorillonite	47.9975	0.0851	0.0070		0	0–100	0.05598		
						10	0–100	0.05447		
						20	0–100	0.04879		
						30	0–100	0.0364		
						40	0–100	0.0336		
						50	0–100	0.02865		
	Illite	31	0.0994	0.0028		0–50	0–100	0		
	kaolinite	16.57	0.0846	0.0016		0–50	0–100	0		
	chlorite	2.96	0.0069	0.0003		0–50	0–100	0		
Masoudi et al. (2025)^74^	smectite	27	0.11			50	0–100	0.038	vacuumed for 2 min to measure the dead volume	volumetric (BELSORP-HP, MicrotracBEL Corp., Japan)
	montmorillonite	70	0.09			50	0–100	0.067		
	kaolinite	11.2	0.04			50	0–100	0		
	Montmorillonite_semi_dry	70	0.09			50	0–100	0.096	dried for 200 min at 50 °C under vacuum	
	Smectite_dry	27	0.11			50	0–100	0.052	dried overnight at 50 °C under vacuum	
	Montmorillonite_dry	70	0.09			50	0–100	0.432		
	Kaolinite_dry	11.2	0.04			50	0–100	0		

**Figure 1:**
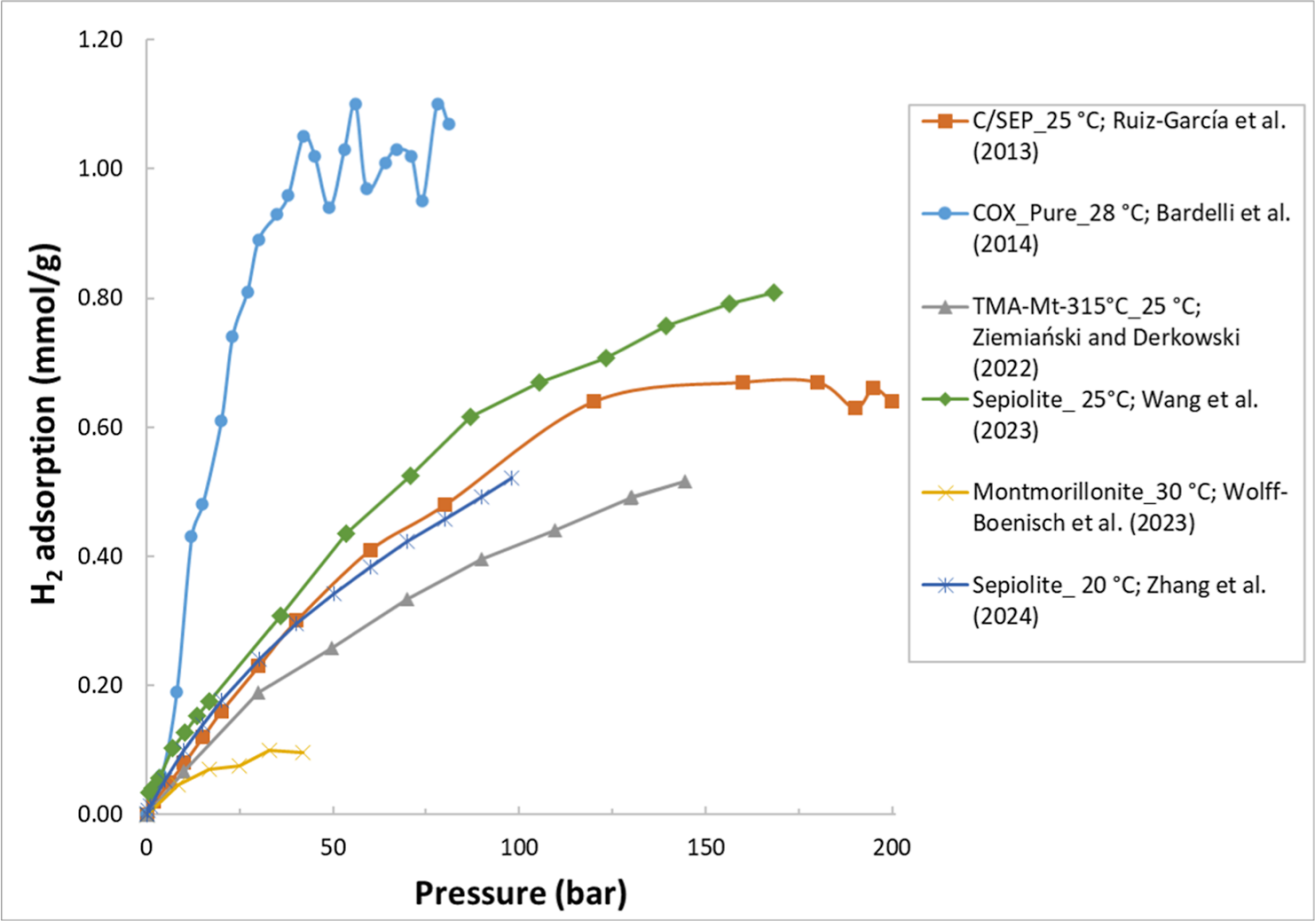
Example H_2_ adsorption isotherms for the selected clay minerals. The legend format is [sample name]_[test temperature]; reference. Data are from refs 23, 43, 45, 67, 69, and 73 .

Didier et al. (2012)^47^ experimentally investigated H_2_ gas adsorption and the reduction of structural Fe­(III) in various clays, including synthetic Na-montmorillonite with varying Fe­(III) content (ranging from 0 wt % to 6.4 wt %), COx clay rock, and its purified clay fraction. This study differs from others mentioned here due to its unique experimental procedure. They employed gas chromatography and exposed the samples to a mixture of Ar/H_2_ (95:5%) over an extended experimental period of 30–45 days (at a partial pressure of 0.45 bar at temperatures of 90 and 120 °C).

The results revealed that the clays adsorbed up to 0.11 wt % for synthetic Na-montmorillonite, 0.06 wt % for pure COx, and 0.05 wt % for the raw COx sample. These findings were correlated with the specific surface areas of the clay samples. No discernible relationship was observed between the Fe­(III) content and the amount of H_2_ gas sorbed. The similar levels of H_2_ gas adsorption in both the pure and raw COx samples were attributed to possible interactions of CaCO_3_, SiO_2_, and FeS_2_ (present in the raw sample) with H_2_, likely due to the longer experimental duration. It was concluded that over 18 m^3^ of H_2_ could be adsorbed per cubic meter of raw COx. Mössbauer spectroscopy revealed up to 6% reduction of structural Fe­(III) in synthetic montmorillonites but no significant Fe­(III) reduction in COx samples due to H_2_ adsorption under repository-relevant conditions. This indicates that natural COx clay rock remains geochemically stable in the presence of H_2_ at 90 °C and a low partial pressure.

The COx sample is the only clay-rich sedimentary rock in this section; all other samples are purified clay or single clay minerals.

Ruiz-García et al. (2013)^67^ investigated the potential of carbon-clay nanocomposites as a medium for H_2_ storage. The samples were prepared by impregnating MMT and sepiolite with caramel. At 298 K and 200 bar, the carbon–sepiolite nanocomposite exhibited H_2_ adsorption of 0.14 wt %, while the carbon–montmorillonite nanocomposite showed a H_2_ adsorption of 0.07 wt %, both relative to the total system mass. It is noteworthy that thermally treated pure clays showed no detectable H_2_ adsorption. Some gas desorption data are reported for H_2_ without any discussion. The adsorption–desorption isotherms showed a small hysteresis loop, with the desorption branch lying slightly below the adsorption branch at the same pressure. Such behavior is atypical for physisorption systems and is most plausibly attributable to experimental uncertainty rather than true inverse hysteresis behavior.

Bardelli et al. (2014)^45^ examined H_2_ uptake on the same samples of the COx clay sample and its purified clay fraction as Didier et al. (2012),^47^ but at higher pressures more closely matching nuclear waste repository conditions. They employed the volumetric method to measure H_2_ adsorption up to 90 bar at temperatures of 28 and 90 °C. The experiments employed dried samples, and the resulting adsorption isotherms were classified as Type I in the BDDT system (equivalent to IUPAC Type I). At 90 °C, the average H_2_ adsorption was 0.12 wt % for the raw COx sample and 0.24 wt % for the purified COx sample at the plateau. At 28 °C, these values slightly decreased to 0.1 wt % for the raw sample and 0.2 wt % for the purified sample, indicating a temperature-dependent trend not observed in other studies (a trend where adsorption increased slightly with temperature). These results correlated well with the BET surface area measurements. The adsorption isotherms were analyzed using the Freundlich, Langmuir, and Toth models, with the Toth equation providing the best fit to the experimental data. Additionally, a test on the purified sample at 20 K revealed a Type IV H_2_ isotherm, conclusively indicating the presence of both mesopores and micropores. Notably, the reported H_2_ uptake of 850 μmol/g further highlighted an unusual direct relationship with the temperature. The authors reported the temperature dependence to be very low and did not provide an explanation for the counterintuitive temperature trend observed.

Mondelli et al. (2015)^68^ conducted H_2_ adsorption experiments on the same synthetic Na–montmorillonite samples used by Didier et al. (2012),^47^ varying the Fe­(III) content to 0, 3, and 6 wt %. They performed high-pressure tests up to 90 bar. The resulting isotherms were classified as Type I in the BDDT system (equivalent to IUPAC Type I). Both the Fe-free and Fe-bearing samples achieved an adsorption capacity of 0.2 ± 0.02 wt % (approximately 1.0 mmol/g) at the plateau, leading to the conclusion that iron has minimal impact on the texture and sorption capacity of the synthesized clay materials. To characterize the experimental H_2_ isotherms, they employed three models: Freundlich, Langmuir, and Toth. Consistent with previous studies, the Toth equation provided the best fit for the experimental data.

Truche et al. (2018)^42^ investigated the capacity of clay minerals to adsorb H_2_ in the rock samples from the Cigar Lake uranium deposit in northern Saskatchewan, Canada, using the thermal desorption method. They showed that up to 500 ppm (0.25 mmol/g of rock) of H_2_ was trapped by clay minerals (chlorite, Illite, and kaolinite), providing field evidence of H_2_ sorption in clays. Because of the low adsorption capacity of Illite and kaolinite, sudoite (a type of chlorite) was expected to be the main mineral responsible for H_2_ adsorption. The findings suggest that clay minerals’ ability to trap H_2_ could significantly influence the fate and mobility of H_2_ in the crust. In this study, they performed tests on both nontreated and treated (crushed and dried) samples but observed no differences. Due to the significantly different methodology employed, their data are not included in Table 1.

In 2022, Ziemiański and Derkowski^69^ conducted a study on high-pressure H_2_ adsorption and desorption in two types of clays: swelling clays from the smectite group and nonswelling clays from the Illite group. They controlled the interlayer water content and spacing by subjecting the samples to drying temperatures ranging from 40 to 315 °C. This approach allowed them to examine how structural and textural factors influence H_2_ adsorption in clay minerals and to quantify their impacts on total H_2_ adsorption.

Their findings revealed that higher drying temperatures, which reduced the interlayer water content and interlayer height, resulted in decreased H_2_ adsorption. These results were consistent with their previous studies on CH_4_ adsorption using similar samples.^70,71^ By manipulating the intercalation of H_2_ and CH_4_, they highlighted the importance of “structurally controlled interlayer adsorption”. Due to its smaller size compared to CH_4_, H_2_ achieved a greater degree of interlayer adsorption, primarily because of improved accessibility of the interlayers to H_2_ molecules.

No correlation was observed between the amount of H_2_ adsorption and the textural parameters derived from nitrogen adsorption isotherms such as microporosity and BET surface area. They attributed this difference to the observation that, unlike H_2_, N_2_ is not believed to diffuse into the smectite interlayers under typical cryogenic measurement conditions (at 77 K). Instead, N_2_ is predominantly adsorbed in the meso- and micropores formed due to the irregular stacking of clay mineral layers and at the edges of the crystallites. However, they observed a relatively good correlation between the CO_2_ micropore volume (derived from CO_2_ adsorption isotherms using the Dubinin–Radushkevich equation) and H_2_ adsorption in nonswelling clay minerals. This suggests that, in nonswelling clay minerals, most of the H_2_ adsorption occurs at sites accessible to CO_2_ molecules at 0 °C under 1 bar, but not at sites accessible to CH_4_ (at 25 °C) or nitrogen gas (at 77 K). They have not observed any significant hysteresis loop, and H_2_ adsorption and desorption isotherms overlapped within the analytical error.

Wolff-Boenisch et al. (2023)^23^ measured the adsorption capacity of MMT at pressures up to 50 bar and temperatures of 77, 195, and 303 K. They used Langmuir and Freundlich models to fit the experimental data, and both followed IUPAC Type I isotherms. The Langmuir model showed a slightly better fit. Additionally, they suggested a surface area-normalized metric by reporting H_2_ adsorption capacity as volume adsorbed per specific surface area (μL/m^2^) rather than an absolute value. Their analysis suggested H_2_ storage in deeper reservoirs since higher pressure and temperature result in higher storage volumes and lower H_2_ loss through adsorption in the caprock, considering that the molar volume of H_2_ decreases significantly at higher pressures.

Wang et al. (2023)^43^ measured the H_2_ adsorption capacity of six clay minerals at various temperatures (0, 25, 45, and 75 °C) and pressures (up to 180 bar). They reported maximum H_2_ adsorption for sepiolite and palygorskite, while they did not see any detectable quantities of adsorbed H_2_ for kaolinite and Illite. They concluded that the pore structure, specific surface area, and pore volume (especially the pores with a size of less than 30 nm) of clay play important roles in their H_2_ adsorption capacity. They used the Langmuir equation to fit the experimental data.

Ghosh et al. (2023)^72^ conducted an experimental study on H_2_ adsorption in various bentonite and MMT samples under both low-pressure–low-temperature (LPLT, 77 K) and high-pressure–high-temperature (HPHT, 313 K) conditions. The study revealed that specific surface area and micropore volume positively influence H_2_ adsorption, whereas the average pore width has a negative effect. They also evaluated the applicability of several established adsorption models, including Langmuir, Freundlich, Toth, and Sips. Toth and Sips models showed good performance in both LPLT and HPHT settings.

Interestingly, one sample with a high micropore volume (a bentonite with 81% microporous volume) exhibited higher adsorption capacity under HPHT conditions compared to under LPLT conditions. This increased adsorption capacity at HPHT was attributed to enhanced gas dosing and better pore accessibility under these conditions. From these observations, the authors suggested that micropores play a crucial role in H_2_ adsorption in clay at HPHT conditions.

Zhang et al. (2024)^73^ investigated the adsorption and desorption dynamics of H_2_ and cushion gases (CO_2_, CH_4_, and N_2_) on five clay minerals—montmorillonite, Illite, chlorite, sepiolite, and kaolinite—under reservoir conditions. Sepiolite demonstrated the highest H_2_ adsorption capacity, over 12 times that of MMT, while Illite, chlorite, and kaolinite showed negligible adsorption. The Langmuir adsorption model effectively described the experimental data.

In their desorption tests, MMT exhibited pronounced hysteresis with approximately 42.19% of adsorbed H_2_ unrecoverable during desorption. This was attributed to its swelling behavior, which likely causes pore structure alterations, impeding gas release. In contrast, sepiolite showed minimal hysteresis, with only a 3.56% loss, suggesting its fibrous structure facilitates more accessible and reversible gas adsorption.

They also examined cyclic adsorption–desorption to assess the operational stability under repeated pressure variations. MMT displayed inconsistent adsorption capacity across cycles, likely due to structural changes caused by hysteresis effects. Conversely, sepiolite maintained consistent adsorption behavior across multiple cycles, indicating superior stability and predictability for repeated use.

Masoudi et al. (2025)^74^ examined the impact of hydration on H_2_ sorption in three pure clay fractions and four shale caprocks from the Norwegian Continental Shelf. Although they did not have any control over the moisture content, they showed that H_2_ uptake is significantly reduced under hydrated conditions due to competition between water molecules and H_2_ for sorption sites. They employed the Langmuir, Toth, and Freundlich models to fit the experimental data, with the Freundlich model providing the best fit to the sorption isotherms. Results indicate that dried clay powders represent the maximum sorption capacity, whereas naturally hydrated samples showed diminished H_2_ sorption. The study emphasizes that mineralogy, hydration levels, and organic content influence H_2_ sorption, highlighting the complexities of using natural clay-rich systems under realistic geological conditions. Additionally, desorption experiments revealed the presence of a hysteresis between adsorption and desorption isotherms. This hysteresis is attributed to several factors, including capillary action within micropores, varying pore sizes, and the high diffusivity of H_2_ in the shale matrix. The data for the four shale caprocks are listed in Table 2 (shales).

**Table 2:** Summary of H_2_ Adsorption Studies on Shales

refs	sample	composition (wt %)	TOC (wt %)	BET surface area (m^2^/g)	pore volumes (cm^3^/g) [micropore volumes %]	average pore diameter (nm)	*T* (° C)	pressure range (bar)	H_2_ adsorption [* at plateau] (mmol/g)	sample treatment	experimental setup
Abid et al. (2022)^22^	eagle ford shale reservoir rock: fresh shale	calcite: 89.3	3.83	2.09	0.0192 [0.77]	36.93	30	0–42.8	*0.06	dried in the oven at 333 K for 24 h, and the cell was vacuumed for at least 1 h at 333 K	volumetric (PCT-Pro adsorption analyzer, SETARAM)
		pyrite: 0.5									
		quartz: 10.2									
	Eagle Ford shale reservoir rock: aged shale with humic acid		NA	3.815	0.0117 [1.67]	20.67	30	0–42.8	0.32	powder (particle size < 45 μm) aged with humic acid solution for 2 h and then was preheated in the oven at 333 K for 48 h	
Alanazi et al. (2023)^21^	Jordanian organic and carbonate-rich source rocks	calcite: 85	17.94	3.08	0.032	41.42	60	0–100	*0.18	heated and dried in the oven at 80 °C for 48 h, then heated under vacuum at 60 °C for approximately 12 h	volumetric (PCTpro-2000, SETARAM, Hong Kong)
		quartz: 14								powder in cells was evacuated for at least 2 h at 60 °C	
		berlinite: 1									
		calcite: 92	16.15	3.68	0.031	34.60	60	0–100	*0.23		
		quartz: 4									
		apatite: 4									
		calcite: 73	14.89	3.53	0.024	27.24	60	0–100	*0.43		
		quartz: 14									
		apatite: 12									
		langite: 1									
		calcite: 44	12.93	3.19	0.011	14.73	60	0–100	*0.47		
		quartz: 54									
		apatite: 1.4									
		berlinite: 0.5									
Wang et al. (2024)^19^	Longmaxi formation shale, Sichuan Basin, China	quartz: 36.3	3.91	8.393	0.027	3.847	30	0.05–186	0.16	NA	volumetric (in house instrument)
		dolomite: 34					45	0.05–155	0.12		
		calcite: 15.3					60	175	0.11		
		Illite: 13.7									
		kaolinite: 0.7									
Wang et al. (2024)^85^	clay-rich Chang 7 member of the Yanchang formation shale in the Ordos Basin (sample 412)	clay: 41	9.684	5.811	0.047	32.02	25	0–180	0.041	not indicated for H_2_ tests	volumetric (BSD-PH)
		quartz: 12								for N_2_ tests: vacuum-degassed for 12 h at 105 °C	
		microcline: 27					45	0–180	0.035		
		dolomite: 3									
		plagioclase: 13					65	0–180	0.031		
		pyrite: 4									
	sample 413	clay: 45	7.992	6.568	0.050	30.69	25	0–180	0.055		
		quartz: 12					45	0–180	0.052		
		microcline: 19					65	0–180	0.045		
		dolomite: 2									
		plagioclase: 17									
		pyrite: 5									
	sample 427	clay: 44	14.32	6.097	0.050	32.50	25	0–180	0.057		
		quartz: 8					45	0–180	0.053		
		microcline:15					65	0–180	0.050		
		dolomite: 15									
		plagioclase: 12									
		pyrite: 6									
Al-Harbi et al. (2023)^83^	Midra shale (palygorskite rocks), Quarry, Qatar	rich in silicate clay minerals (58.83% SiO_2_)	0.74				50	20	0.275	dried at 80 °C for 24 h	gravimetric (Rubotherm magnetic suspension balance (MSB) setup)
							70	20	0		
							100	20	0		
Alanazi et al. (2025)^84^	Jordanian organic- and carbonate-rich source rocks	calcite: 44	12.93	3.19	0.011	14.73	–80	0–64	*0.46	heated in an oven at 100 °C for 24 h, and the cell was evacuated for 2 h at 60 °C	volumetric (PCTPro-2000, SETARAM, Hong Kong)
	samples (JO-1)	quartz: 54					0	0–64	*0.22		
		apatite: 1.4					30	0–64	*0.13		
		berlinite: 0.5					60	0–64	*0.12		
	JO-2	calcite: 85	17.94	3.08	0.032	41.42	–80	0–64	0.77		
		quartz: 14					0	0–64	0.4		
		berlinite: 1					30	0–64	*0.22		
							60	0–64	*0.14		
Masoudi et al. (2025)^74^	shale caprocks from the Norwegian Continental Shelf (Hekkingen)	quartz: 24.2	19.4	5.3	0.02		30	0–100	0.072	vacuumed for 2 min to measure the dead volume	volumetric (BELSORP-HP, MicrotracBEL Corp., Japan)
		feldspar: 1.7					50	0–100	0.065		
		calcite: 2.8					70	0–100	0.056		
		pyrite: 5.6									
		microcline: 2.9									
		dolomite: 1.7									
		clay: 61									
	Draupne shale	quartz: 22.4	6.8	7.1	0.03		50	0–100	0.022		
		calcite: 0.7									
		pyrite: 7.7									
		microcline: 17.8									
		dolomite: 1.2									
		clay: 50.7									
	Rurikfjellet shale	quartz: 26	3.1	3.3	0.01		50	0–100	0.027		
		feldspar: 8									
		pyrite: 1									
		siderite: 5									
		clay: 60									
	Agardfjellet shale	quartz: 13	4.6	22	0.05		50	0–100	0.050		
		feldspar: 6									
		ankerite: 7									
		calcite: 29									
		clay: 45									
	Hekkingen_dry	quartz: 24.2					50	0–100	0.081	dried overnight at 50 °C under vacuum	
		feldspar: 1.7									
		calcite: 2.8									
		pyrite: 5.6									
		microcline: 2.9									
		dolomite: 1.7									
		clay: 61									
	Draupne_dry	quartz: 22.4					50	0–100	0.033		
		calcite: 0.7									
		pyrite: 7.7									
		microcline: 17.8									
		dolomite: 1.2									
		clay: 50.7									
	Rurikfjellet_dry	quartz: 26					50	0–100	0.039		
		feldspar: 8									
		pyrite: 1									
		siderite: 5									
		clay: 60									
	Agardfjellet_dry	quartz: 13					50	0–100	0.034		
		feldspar: 6									
		ankerite: 7									
		calcite: 29									
		clay: 45									

Several attempts have been made to explore the suitability of clay materials as adsorbents for H_2_ storage. Notably, minerals like Laponite,^75,76^ sepiolite,^77–79^ and halloysite^80^ have emerged as promising candidates for H_2_ storage owing to their favorable properties. However, it is important to recognize that these minerals are not typically found in sedimentary rocks, which diminishes their relevance for geological H_2_ storage. Furthermore, there are several studies on low-temperature, low-pressure H_2_ adsorption on clay minerals like bentonite and MMT.^77,81,82^ These studies also have not been covered in detail here. Consequently, we will briefly acknowledge their potential here without going into further details.

### 3.2 Shale

There have also been some attempts to study the H_2_ sorption on various shale formations. They employed source rock or reservoir rocks to investigate H_2_ sorption as a trapping mechanism that enhances the storage capacity of shale reservoirs.^19,21,22^ In the following, we present the shale studies. Relevant high-pressure and high-temperature data for these studies are summarized in Table 2. Figure 2 illustrates example H_2_ adsorption isotherms for some of the shale samples reviewed in this section.

**Figure 2:**
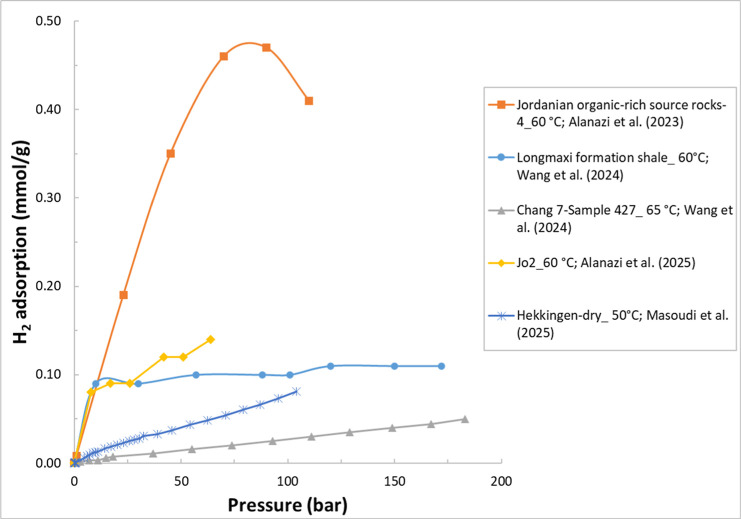
Example H_2_ adsorption isotherms for selected shale samples. The legend format is [sample name]_[test temperature]; reference. Data are from refs 19, 22, 74, 84, and 85 .

In a study conducted by Abid et al. (2022),^22^ the impact of humic acid on the adsorption capacity of Eagle Ford shale was examined by measuring H_2_ and CH_4_ adsorption on both raw and aged shale samples at 30 °C and pressures up to 42.8 bar. The findings revealed that aging the shale sample with humic acid increased its H_2_ adsorption capacity by more than five times, while having no significant effect on CH_4_ adsorption. This substantial increase in H_2_ adsorption was attributed to several factors: the shale’s smaller pore size, higher BET surface area, and micropore content, as well as the formation of a hydrophobic layer on the shale surface.

The researchers also noted that increasing the total organic carbon (TOC) content by adding humic acid could potentially result in higher H_2_ adsorption. It is worth mentioning that the shale powder used for aging had a smaller particle size (<45 μm) and was dried for a longer duration (48 h) compared to the raw shale powder, which had a particle size of 250 μm and was dried for 24 h. While humic acid contributed to enhancing the H_2_ sorption capacity, the use of finer powder particles and longer drying times may also have played a role in this effect.

In their experimental investigation, Alanazi et al. (2023)^21^ studied the adsorption of H_2_, CH_4_, and CO_2_ on organic-rich source rocks (the Jordanian source rocks from the Muwaqqar Chalk-Marl formation) at 60 °C and pressures up to 100 bar. They found that the adsorption capacities for CO_2_ and CH_4_ were significantly higher than those for H_2_, being up to 5 and 23 times greater, respectively. They reported a reduction in H_2_ adsorption as the TOC content increased (contrasting with conventional expectations). They performed a thorough mineralogical quantification and concluded that calcite, carboxyl, and sulfonyl functional groups augment H_2_ adsorption on carbonate samples. The observed increase in H_2_ adsorption in this study can also be attributed to the distribution of pore sizes, where the samples exhibiting higher H_2_ adsorption possess a smaller average pore size.

A gravimetric experimental investigation by Al-Harbi et al. (2023)^83^ on the adsorption and desorption characteristics of a Midra shale sample from Qatar, mainly composed of silicate clay minerals, revealed low H_2_ uptake at 50 °C and pressures up to 20 bar. However, the reported value (0.56 mg of H_2_/g of rock, or approximately 0.275 mmol of H_2_/g of rock) is not at the lowest end of the spectrum for H_2_ adsorption on shales (see Table 2). For instance, Wang et al. (2024)^19^ reported adsorption with even lower uptake amounts (from 0.11 to 0.16 mmol H_2_/g rock), concluding that H_2_ can be stored as an adsorbed phase on shale. Similarly, Alanazi et al. (2025)^84^ identified adsorption as an important trapping mechanism for H_2_ storage, reporting capacities of 0.12 to 0.22 mmol/g for organic-rich Jordanian shale at higher, more relevant temperatures.

Nevertheless, at temperatures of 75 and 100 °C and pressures up to 20 bar, Al-Harbi et al. (2023)^83^ observed no H_2_ uptake. The desorption results showed a small hysteresis for the tests at 50 °C. They concluded that the low adsorption affinity indicates that H_2_ is neither chemically nor physically retained by the rock surface. This finding highlights the reduced risk of H_2_ loss and rock deformation, thereby supporting the potential for safe and efficient storage and recovery in such formations.

Ho et al. (2024)^20^ employed nuclear magnetic resonance (NMR) to investigate the adsorption and diffusion behaviors of H_2_ and CH_4_ in organic-rich Duvernay shale and Berea sandstone. The results indicated negligible H_2_ adsorption in sandstone, whereas both free and adsorbed H_2_ were observed in the Duvernay shale sample. During unloading, the NMR response in Duvernay shale exhibited hysteresis in gas adsorption–desorption, indicating an approximate 10% loss of H_2_.

Wang et al. (2024)^19^ aimed to assess the potential for H_2_ storage in shale reservoirs. They conducted H_2_ adsorption tests on shale reservoir samples (the Longmaxi formation shale, collected from the Sichuan Basin, China) under high-pressure, high-temperature conditions (30–60 °C and 5–200 bar). The results demonstrated that the H_2_ adsorption capacity of shale ranged from 65% to 80% of the CH_4_ adsorption capacity. To model the H_2_ adsorption behavior, they compared four isothermal adsorption models: Langmuir, Freundlich, BET, and Dubinin–Astakhov models. Among these, the Freundlich model was the most suitable for describing the H_2_ adsorption behavior of the tested shale samples. The researchers also examined the H_2_ adsorption capacity of organic-free samples and found it to be approximately 64–75% of that of kerogen-containing samples. This indicates that kerogen, the organic component of shale, plays a crucial role in the overall H_2_ adsorption capacity of the shale reservoirs.

Wang et al. (2024)^85^ examined the H_2_ adsorption capacity of the Triassic Chang 7 Shale Member in the Ordos Basin. Their samples were characterized by a high clay content (ranging from 41% to 45%) and high organic content (TOC ranging from 8% to 14%). The adsorption behavior was found to follow both the Freundlich and Langmuir isotherm models, with the Freundlich model providing a better fit. The study concluded that organic matter and clay minerals significantly influence H_2_ adsorption. Samples with higher organic content exhibited greater adsorption capacity, and adsorption was positively correlated with specific surface area as well as micropore and mesopore volumes.

Alanazi et al. (2025)^84^ investigated H_2_ adsorption on two organic- and carbonate-rich Jordanian oil shale samples at temperatures of 193, 273, 303, and 333 K and pressures up to 64 bar. They also characterized H_2_ adsorption kinetics at the same temperatures and two pressures (15 and 45 bar) and provided valuable kinetic data. The experimental data were fitted with two different kinetic models. Results indicated a positive correlation between adsorption capacity and both TOC and calcite content and a negative correlation with quartz content. The TOC correlation contradicts their earlier findings on the same samples (Alanazi et al., 2023 ^21^). Langmuir, Freundlich, and BET models were fitted to the adsorption isotherms, with the BET model providing the best fit.

As explained in the previous subsection, Masoudi et al. (2025)^74^ examined the impact of hydration on H_2_ sorption in three pure clay fractions and four shale caprocks from the Norwegian Continental Shelf. Their results showed that H_2_ adsorption is heavily influenced by mineral composition, organic content, and the degassing (hydration) state. The Hekkingen shale, with the highest clay and organic content, exhibited the greatest H_2_ sorption capacity, while the Agardhfjellet shale, with low clay content and carbonate-rich composition, showed the least. Dried shales generally had higher sorption capacities compared to untreated (hydrated) samples, as hydration reduced available sorption sites due to water occupying the pores. Although they reported a positive correlation between the BET surface area of pure clay minerals and their H_2_ adsorption, no such correlation was found between the BET surface area and H_2_ adsorption in shale samples. No consistent correlation between the TOC and sorption capacity was observed, though the higher TOC of Hekkingen shale appeared to enhance its H_2_ uptake. Desorption data revealed hysteresis across all samples, particularly in organic-rich shales, complicating H_2_ recovery.

### 3.3 Coal

This section provides an overview of studies on H_2_ sorption–desorption in coal. Table 3 summarizes the high-pressure and high-temperature data, along with sample characterization, from these studies. Figure 3 illustrates an example H_2_ adsorption isotherm for some shale samples reviewed in this section.

**Table 3:**
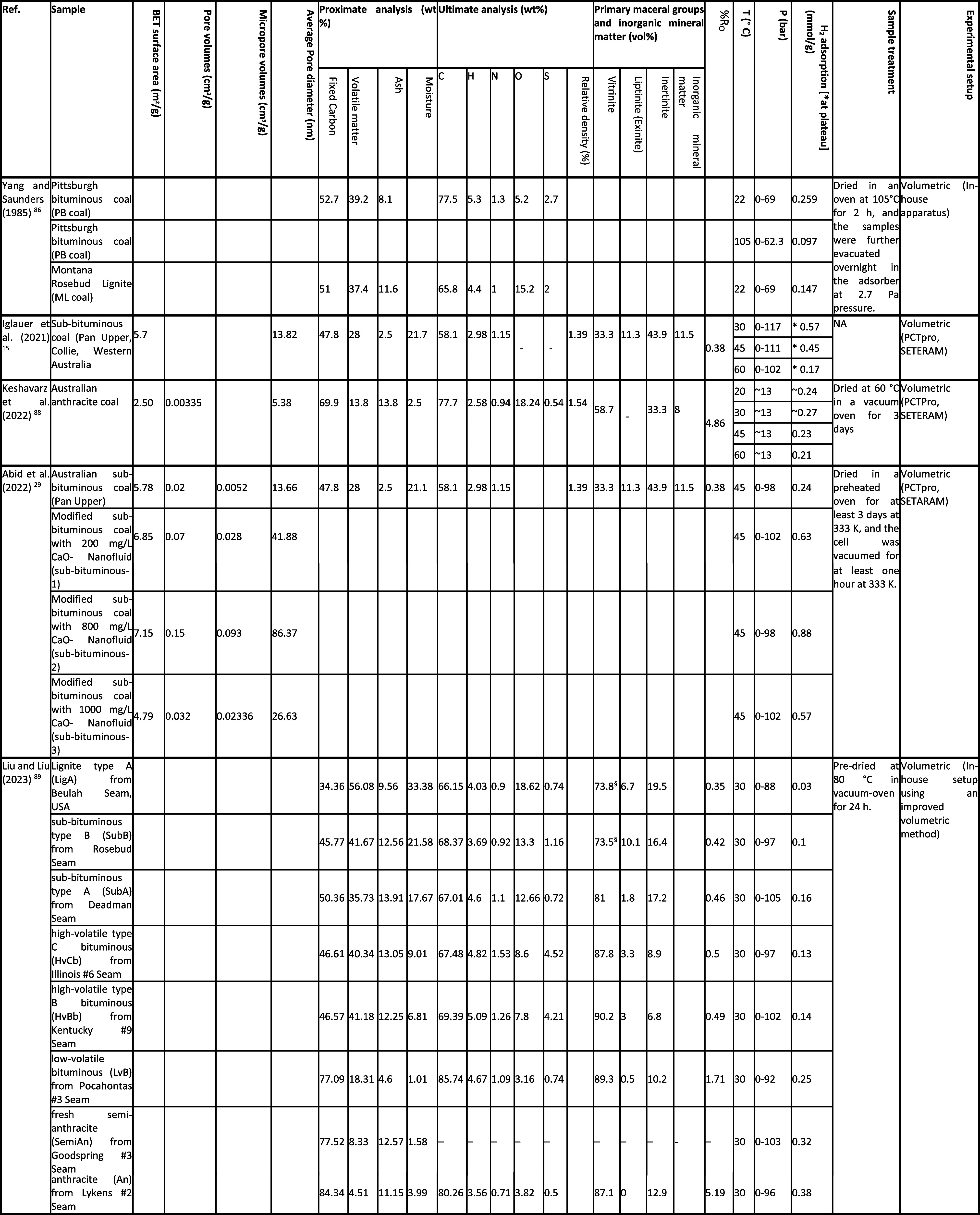

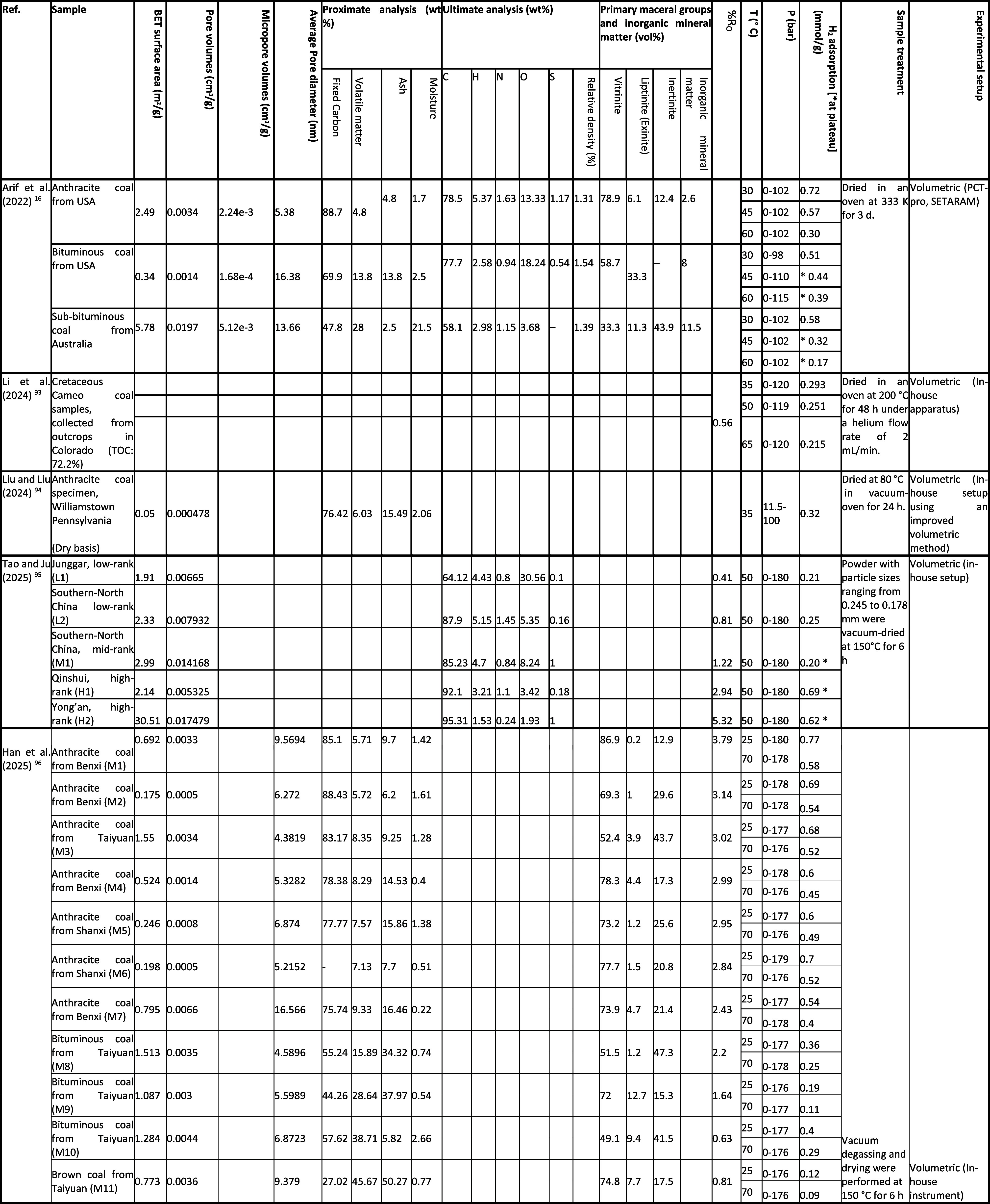
Summary of H_2_ Adsorption Studies on Coal

**Figure 3:**
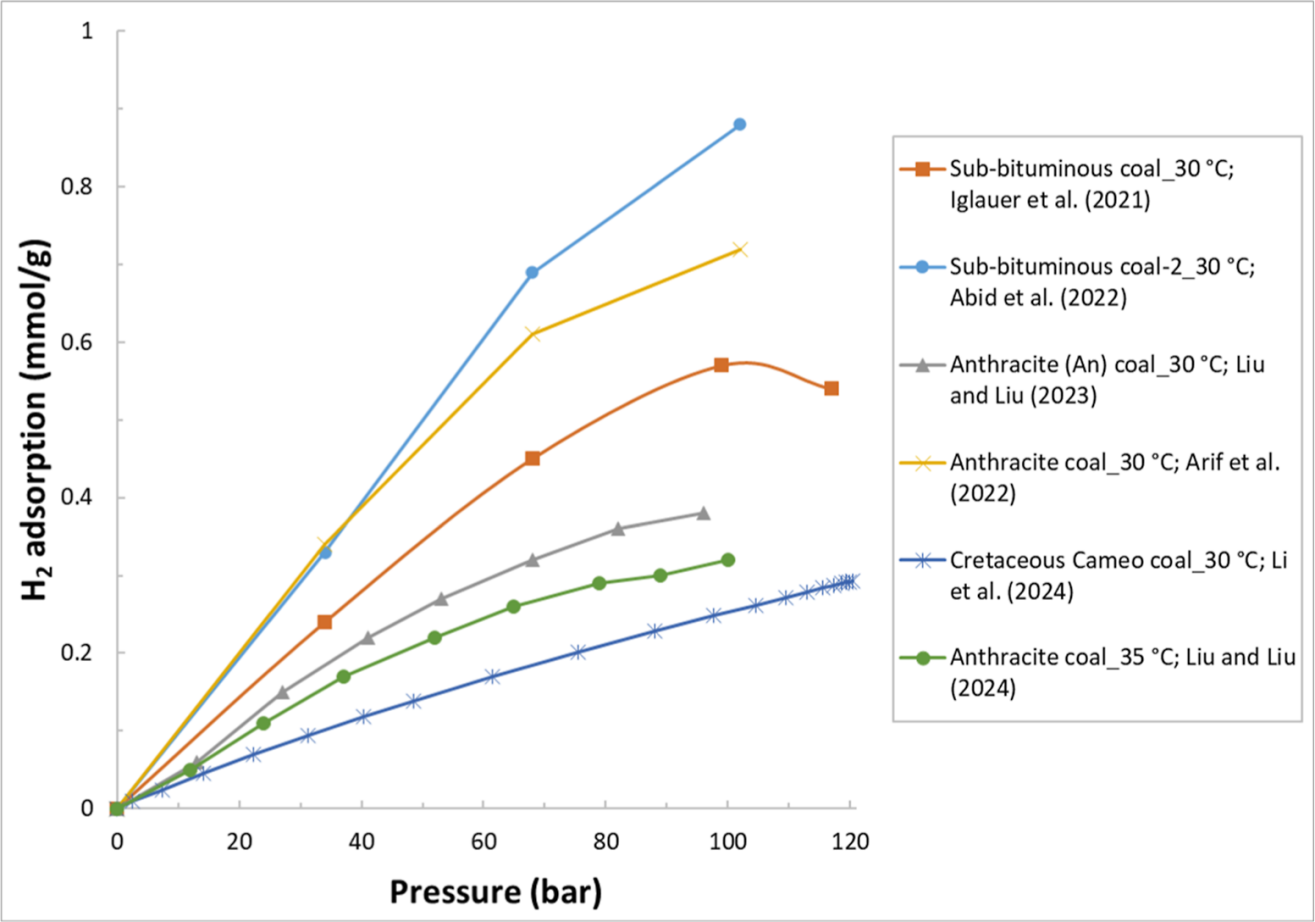
Example H_2_ adsorption isotherms for the selected coal samples. The legend format is [sample name]_[test temperature]; reference. Data are from refs 15, 16, 29, 89, 93, and 94 .

Some of the earliest data of high-pressure high-temperature H_2_ adsorption on coal belong to works of Yang and Saunders (1985)^86^ and Saunders et al. (1985).^87^ They studied the adsorption of H_2_ and CH_4_ on various raw and heat-treated coal samples as selective sorbents for H_2_ and CH_4_ separation. The outcomes demonstrated pronounced selectivity by the heat-treated coals for CH_4_ over H_2_ when dealing with H_2_–CH_4_ gas mixtures. In Table 3, only the available data from raw coal is reported.

Iglauer et al. (2021)^15^ conducted H_2_ adsorption experiments on a sub-bituminous coal sample at different pressures and temperatures (up to 143 bar and 303–333 K). They found that sub-bituminous coal can adsorb up to 0.60 mmol/g at 143 bar and 318 K. The Type I adsorption curve was observed. The study also compared H_2_ adsorption to CO_2_ adsorption, finding that CO_2_ adsorbs in much higher amounts on coal due to stronger interactions with the coal surface (as expected).

Keshavarz et al. (2022)^88^ examined the H_2_ and CO_2_ adsorption and diffusion rates in Australian anthracite coal at different temperatures and pressures of around 13 bar. Their results showed that (as expected) the CO_2_ adsorption capacity is about five times higher than that of H_2_ at all studied temperatures. Additionally, they observed that as the temperature rises, the adsorption rate of H_2_ and CO_2_ in coal increases, and temperature significantly affects adsorption kinetics.

Arif et al. (2022)^16^ investigated the capacity of three different coals with different ranks (anthracite, bituminous, and sub-bituminous) to adsorb H_2_ at high pressures and temperatures (0–102 bar and 303–333 K). The results indicated that high-rank coals with higher vitrinite and carbon content demonstrate greater H_2_ adsorption. Samples exhibited Type I adsorption behavior. Additionally, mineral content had a negative effect on the adsorption capacity of coals. They did not observe a direct correlation between micropore content and BET surface area of coal with their H_2_ adsorption capacity and suggested that these factors alone cannot explain the H_2_ adsorption capacity. They emphasized the complex effect of surface chemistry on the H_2_ adsorption capacity in coal.

In their 2022 study, Rasool Abid et al.^29^ sought to develop a cost-effective material that would enable efficient separation of H_2_ from CO_2_. They investigated the adsorption properties of an Australian sub-bituminous coal sample that had been modified with varying concentrations of CaO nanoparticles. Their findings revealed that the selectivity for CO_2_–H_2_ adsorption hit its maximum at the lowest pressure applied and increased with the highest amount of CaO nanoparticle loading.

Liu and Liu (2023)^89^ evaluated the potential of eight US coals with different coal ranks for H_2_ storage by examining their sorption and diffusion behaviors at 30 °C and up to around 110 bar. To overcome the challenges associated with measuring low H_2_ adsorption capacities, they designed and built an in-house differential volumetric apparatus adapted for coal samples by using larger sample masses (∼50 g). This setup features two parallel high-pressure volumetric setups connected by a low-range differential pressure transducer. The system employs symmetrical sample and reference sides with carefully matched void volumes using adjustable stainless-steel spheres, enabling the precise detection of small pressure decays during H_2_ sorption. Further details regarding the experimental setup can be found in their earlier work.^90^ More on differential volumetric apparatus can be found in Blackman et al. (2006)^91^ and Sircar et al. (2013).^92^

The results showed that high-ranking coals have the highest H_2_ sorption capacities. All samples showed Type I isotherms. As expected, CH_4_ showed higher sorption capacities compared to H_2_ in coal (3.8 times higher for the tested sample). By fitting the H_2_ sorption isotherms with the Langmuir model, they estimated the maximum adsorption capacity (*V*_L_) of the coal samples. Although the modeled amount of maximum H_2_ adsorption capacity is comparable with maximum CH_4_ adsorption capacity, the Langmuir pressure (*p*_L_) for H_2_ sorption is much higher than the Langmuir pressure for CH_4_ sorption (for example, ∼28.2 times for low-volatile bituminous (LvB) coal). Therefore, to attain the highest adsorption capacities in coals, the pressure of H_2_ should be substantially higher than that required for CH_4_. Additionally, they observed that a higher fixed carbon content and lower O/C ratios lead to increased maximum H_2_ adsorption capacity.

In their 2024 study, Li et al.^93^ studied the adsorptive behavior of H_2_, CH_4_, and CO_2_ on Cameo coal at 35 °C, 50 °C, and 65 °C under pressures up to 120 bar to assess the impact of cushion gas on H_2_ loss. Based on their experimental work and subsequent quantitative analyses, they suggested that under H_2_ storage conditions wherein gas mixtures contain more than 8% CH_4_ or over 2% CO_2_, the likelihood of H_2_ loss through adsorption is negligible at depths shallower than 2000 m. However, as they articulated in their publication, it is important to acknowledge that this conclusion is based solely on the adsorption characteristics observed with dried coal powder. Consequently, they advocate that additional research, which accounts for a broader range of factors, is necessary to fully understand the phenomenon.

To isolate true sorption from instrument artifacts and provide more reliable data for H_2_ sorption (which exhibits very low uptake), Li et al. (2024)^93^ emphasized the necessity of using blank experiments for correction. After conducting blank experiments with a nonadsorbing nickel cylinder and subtracting these blanks from the raw data, the corrected H_2_ isotherms were found to rise linearly with pressure, indicating that the excess adsorption of H_2_ in coal does not reach a maximum within the experimental pressure range. The authors attribute the discrepancies between the H_2_ adsorption isotherms observed in their study and those reported in earlier literature (with isotherms reaching a plateau) to a potential failure in previous studies to properly account for H_2_ blank corrections.

In Tables 1–4, we highlighted the H_2_ adsorption isotherms that reached a plateau. Many of the isotherms have reached, and many have not. These variations underscore that H_2_ adsorption is a complex process correlated to numerous parameters. As previously discussed and as will be elaborated later, H_2_ adsorption correlates with many parameters, and complex systems can exhibit complex trends. However, considering the high diffusivity and generally low adsorption levels of H_2_, the conclusion that a correction for experimental artifact is necessary is very relevant. It is crucial to note, however, that sorption behavior is affected by many factors and can vary significantly between different substrates, especially in heterogeneous natural rocks, such as coal and shale.

**Table 4:** Summary of Studies on H_2_ Adsorption in Sandstone, Mudstone, Diatomite, and Diatomaceous Earth

refs	sample	BET surface area (m^2^/g)	pore volumes (cm^3^/g)	average pore diameter (nm)	*T* (°C)	pressure range (bar)	H_2_ adsorption [* at plateau] (mmol/g)	sample treatment	experimental setup
Jin et al. (2014)^101^	natural diatomite (from Jilin, China)	32.439	0.0604	7.44	25	0–26.3	2.297	well-dried samples were completely degassed in vacuum at 513 K (less than 4.0 Pa)	volumetric (in-house)
	acid-thermal activated diatomite (A-diatomite)	25.766	0.0464	7.2	25	0–26.3	4.132		
	palladium-modified diatomite (Pd-diatomite)	18.637	0.0530	11.38	25	0–26.3	4.861		
	platinum-modified diatomite (Pt-diatomite)	15.374	0.0554	14.42	25	0–26.3	3.452		
Mirchi and Dejam (2023)^24^	Berea sandstone, sourced from an Ohio quarry (water-wet)	2.79			25	1–80	0.046	dried in an oven at 110 °C for 3 days. The samples in the sample chamber were vacuumed for at least 15 h at 120 °C	volumetric (HPVA II, Micromeritics)
					40	1–80	0.029		
					60	1–80	0.026		
	Berea sandstone aged with crude oil (oil-wet)				25	1–80	0.044		
					40	1–80	0.028		
					60	1–80	0.023		
Wang et al. (2024)^103^	deep purple mudstone	10.408			25	0–185	0.022	not indicated for H_2_	volumetric (BSD-PH)
					45	0–185	0.019	for N_2_: degassed at 100 °C for 10 h	
					65	0–185	0.018		
	gray sandstone	8.212			25	0–185	0.025		
					45	0–185	0.022		
					65	0–185	0.022		
	gray–green silty mudstone	5.056			25	0–185	0.011		
	gray–green silty mudstone	3.449			25	0–185	0.006		
	gray–green mudstone	8.757			25	0–185	0.025		
					45	0–185	0.023		
					65	0–185	0.022		
	gray–green mixed with purple mudstone	7.272			25	0–185	0.021		
	gray coarse sandstone	2.208			25	0–185	0.004		
	volcanic tuff	6.899			25	0–185	0.014		
	gray fine sandstone	2.959			25	0–185	0.007		
	conglomerate	1.384			25	0–185	0.005		
	gray–green mudstone in conglomerate	4.813			25	0–185	0.012		
Wang et al. (2024)^104^	diatomaceous earth from Jilin, China	18			25	0–180	0.197	not indicated for H_2_	volumetric (BSD-PH, Beijing, China)
					45	0–180	0.152	for N_2_: degassed at 120 °C for 8 h	
					60	0–180	0.143		

In another study, Liu and Liu in 2024 ^94^ investigated the feasibility of injecting a CO_2_–H_2_ gas mixture, produced from gray H_2_ production, into coalbed methane formations for permanent CO_2_ storage and temporary H_2_ storage. As expected, CO_2_ exhibited the highest sorption capacity, followed by CH_4_, both of which were roughly 3–5 times higher than that of H_2_. Hydrogen, however, demonstrates superior diffusive gas deliverability compared to that of CO_2_, with its diffusion coefficient being approximately six times higher. The study also found that H_2_ adsorption caused matrix shrinkage in coal, in contrast to that of CH_4_ and CO_2_ (strong sorbing gases), which induced matrix swelling.

Tao and Ju (2025)^95^ investigated the potential of coal as a medium for H_2_ storage by analyzing how its pore structure and surface chemistry affect adsorption. They performed H_2_ adsorption–desorption tests on five coal samples, ranging from low to high rank, at a temperature of 50 °C and within a pressure range of 0–180 bar. They improved their volumetric apparatus by using a pressure sensor with higher accuracy (although the accuracy of the new sensors was not reported) to overcome the significant measurement errors caused by weak H_2_ affinity in conventional isothermal equipment. They reported that coal rank and pore size distribution have significant impacts on H_2_ adsorption capacity. Coals with higher ranks exhibited more sorption capacity (about four times), faster adsorption rates, and easier desorption (less hysteresis). Micropores (<2 nm) were identified as the dominant contributors to H_2_ uptake, with higher-rank coals showing more developed microporous networks. Additionally, the presence of oxygen-containing functional groups (such as hydroxyl and carboxyl groups) on coal surfaces enhanced the H_2_ interaction via physisorption. Coals with higher oxygen content exhibited 20–30% greater H_2_ uptake compared to those with fewer surface functionalities. Desorption data indicated that 5–12% of the adsorbed H_2_ remained retained within the coal matrix.

Han et al. (2025)^96^ investigated the H_2_ adsorption characteristics of 11 coal samples collected from various Chinese basins. The experiments were conducted at 70 °C and within a pressure range of 0–180 bar. To overcome measurement challenges for weak H_2_–coal interactions, they improved the test accuracy by using more accurate pressure and temperature sensors. Their findings showed that the adsorption capacity is strongly influenced by coal rank. Higher-rank coals exhibited greater sorption capacity due to higher organic matter content, enhanced microporosity, and improved pore connectivity. Organic matter accounted for the majority of H_2_ adsorption, with clay minerals playing a secondary role and brittle minerals contributing negligibly. It should be noted that they also reported H_2_ adsorption data from a separate study of theirs published in Chinese,^97^ conducted on the same coal samples at 25 °C. This data has been included in Table 3.

H_2_ sorption research on coals, much like that on other rocks, is missing critical components: desorption and the impact of moisture. Among the previously mentioned studies, only one presented desorption data, and all conducted their experiments solely on dried coal powders. As a result, the reversibility of H_2_ adsorption on coals and the effects of water’s presence (analogous to the situation in other rocks) remains uncharted territories in the field. Another study addressing coal that included desorption data—but only at low pressures—was carried out by Cygankiewicz et al. in 2012.^98^ They performed H_2_ adsorption/desorption experiments on different Polish hard coals under room conditions (298 K and low pressures not exceeding 0.9 bar). The study sought to determine how certain hard coal characteristics influence the ability to adsorb and desorb H_2_. Findings from the research indicate that factors such as the carbon and oxygen content, surface hydrophobicity, and moisture levels have a notable impact on H_2_ sorption. The results demonstrated that sorption is generally low but varies significantly among different coal samples. It was observed that H_2_ desorption was only reversible in certain coal samples; in other cases, the desorption isotherms lay notably below the corresponding adsorption isotherms at the same pressure, suggesting the concurrent release of other gases or volatile components from the coal matrix and pointing to complex chemical interactions within the coal. Furthermore, the study found that moisture content had a significant effect on H_2_ adsorption; coals with a moisture content between 1 and 4% displayed optimal H_2_ sorption, whereas a higher moisture level (up to 14%) drastically reduced H_2_ adsorption. It is important to note that this study’s data are not included in Table 3 due to its focus on low-pressure ranges.

It should be noted that there are more data on H_2_ adsorption on coal in the literature. However, these studies provide valuable data, but we did not include them here because their experimental condition is not related to the context of this paper, as either the temperature or pressure or both^99,100^ are too low. Additionally, the literature on H_2_ adsorption on activated carbon produced from coal is rich, but it is out of context of this study.

### 3.4 Other Rocks

In this section, we review studies on a diverse group of rocks, including sandstone, mudstone, diatomite, and diatomaceous earth. Summary data from these studies are listed in Table 4. Figure 4 illustrates an example of H_2_ adsorption isotherms for some samples reviewed in this section.

**Figure 4:**
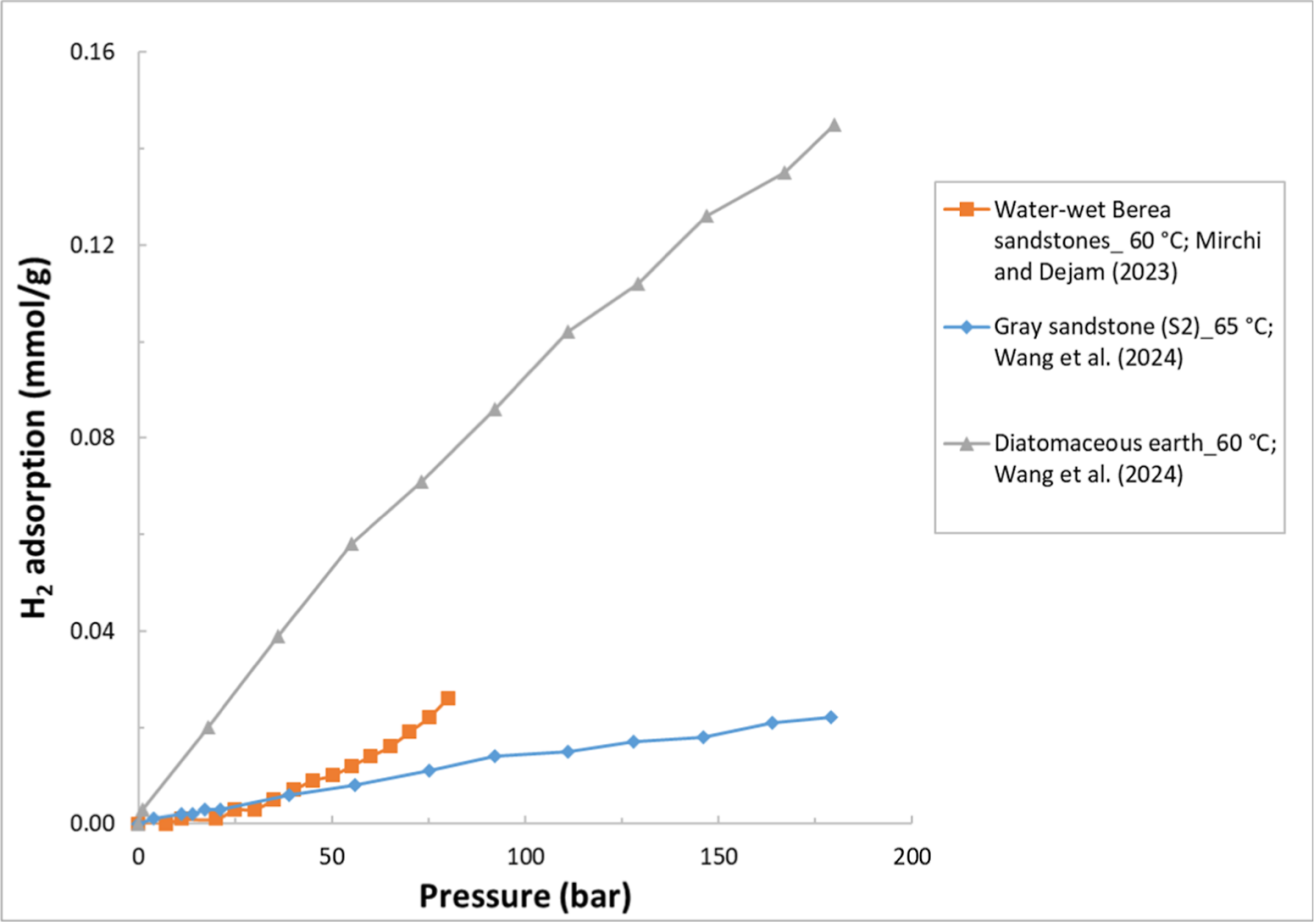
Example H_2_ adsorption isotherms for the selected samples. The legend format is [sample name]_[test temperature]; reference. Data are from refs 24, 103, and 104 .

Jin et al. (2014)^101^ investigated the H_2_ adsorption capacities of natural diatomite subjected to various modifications, including acid-thermal activation and noble metal (Pt and Pd) modifications. The results demonstrated that both diatomite and its modified forms exhibited exceptionally high adsorption capacities, ranging from 0.463 to 0.980 wt % at 26.3 bar and 298 K. The modifications improve the specific surface area and pore structure. Among the modifications, Pd-diatomite showed the highest enhancement, attributed to the strong H_2_ storage capability of palladium. Additionally, the structure and composition of the modified diatomite remained stable after H_2_ adsorption.

Mirchi and Dejam (2023)^24^ investigated the impact of wettability on the adsorption capacity of Berea sandstones for H_2_ and 80–20% H_2_–CH_4_ mixtures at pressures up to 80 bar and three different temperatures (25, 40, and 60 °C). Their research revealed that the sorption capacity of H_2_ on sandstone is significantly lower (by about an order of magnitude) compared to other materials such as coal and shale. It was found that wettability has a minimal effect on the adsorption of pure H_2_. However, H_2_–CH_4_ mixtures exhibited a higher adsorption on oil-wet sandstone. The study also demonstrated that the adsorption of the H_2_–CH_4_ mixture is higher than pure H_2_, suggesting a stronger affinity of CH_4_ for oil-wet rock. The enhanced adsorption capacity was attributed to the interaction between CH_4_ and the C–H functional groups present on the oil-wet sandstone surface. Analysis of the adsorption isotherms revealed that the data aligned more closely with the Freundlich, Redlich–Peterson, and Sips models than with the Langmuir model.

This study was among the few that reported desorption data. They observed hysteresis in the adsorption–desorption isotherms for both H_2_ and H_2_–CH_4_ mixtures (desorption branch above adsorption). The amount of gas desorbed was lower than the amount adsorbed at the same pressure. Additionally, they found that the amount of hysteresis increased with higher temperatures and was more pronounced for the H_2_–CH_4_ mixture. The observed hysteresis was attributed to the interplay of pores with a range of varying sizes and the high diffusivity of H_2_ within the rock matrix.

Carchini et al.^102^ conducted experiments in 2023 to assess the adsorption of H_2_ on intact sandstone and carbonate samples at temperatures of 50 to 100 °C and pressures up to 20 bar. The results of their study indicated that there was no substantial uptake of H_2_ by the samples.

Wang et al. (2024)^103^ investigated the presence and behavior of natural hydrogen in the Songliao Basin. The study analyzed mudstone and sandstone samples from the Denglouku Formation and Yingcheng Formation of Well SK-2 to understand H_2_ adsorption characteristics and their geological implications. The samples primarily consisted of felsic minerals, followed by mica, with a low clay content ranging from 2% to 8%. H_2_ adsorption was found to be low in all samples, particularly in sandstones.

In another study by Wang et al. (2024),^104^ the H_2_ adsorption characteristics of diatomaceous earth from Jilin, China, were investigated. The results indicated that diatomaceous earth possesses a strong H_2_ sorption capacity, achieving a maximum sorption of 4.4 cm^3^/g at 25 °C and 180 bar, surpassing materials like shale and MMT. The adsorption behavior followed the Langmuir isotherm model, revealing that the pressure required for diatomaceous earth to reach sorption saturation is higher than that for MMT. The desorption data exhibited hysteresis; however, above 60 bar, the adsorption and desorption isotherms were closely aligned, nearly overlapping.

## 4 Theoretical Research Methodologies

In this section, we first provide an overview of the current literature on atomistic simulations of H_2_ adsorption and desorption in natural porous materials.

### 4.1 Atomistic Simulations

In this section, we review papers that deal with H_2_ sorption/desorption from a molecular point of view using atomistic simulations, including density functional theory (DFT), Monte Carlo simulations in their multiple ensembles, and molecular dynamics (MD) approaches. These computational methods provide molecular-scale insights into hydrogen–surface interactions, adsorption energies, and transport mechanisms that complement experimental observations. Relevant data and details of these studies are summarized in Table 5.

**Table 5:** Summary of Atomistic Studies on H_2_ Adsorption^a^

refs	type of sample	technique	composition	*T* (K)	*P* (bar)	maximum H_2_ adsorption	porous radio or geometry
López-Chávez et al. (2020)^105^	calcite	DFT	pure H_2_	400–600	1	hydrogen capacity of CaCO_3_ 0.42 mass %	plane wall
Deng et al. (2022)^106^	calcite	GCMC (equilibrium) + MD (diffusion)	pure H_2_	320	300	0.025 mmol/m^2^	slit pore 2.5 nm
						0.04 mmol/m^2^	3.5 nm
						0.05 mmol/m^2^	4.5 nm
				300	300	0.042 mmol/m^2^	3.5 nm
				320		0.040 mmol/m^2^	
				340		0.038 mmol/m^2^	
			binary mixtures (1 of CO_2_/2 H_2_)	300	300	0.006 mmol/m^2^	2.5 nm
				320		0.006 mmol/m^2^	
				340		0.006 mmol/m^2^	
Raza et al. (2022)^107^	kerogen II-A	MD for kerogen GCMC for adsorption	H_2_ at (bulk chemical potential)	323	200	3.00 mmol H_2_/g	2 nm and larger (not defined)
				373		2.60 mmol H_2_/g	
				423		2.25 mmol H_2_/g	
	kerogen II-B			323		3.70 mmol H_2_/g	
				373		2.70 mmol H_2_/g	
				423		2.30 mmol H_2_/g	
	kerogen II-C			323		3.50 mmol H_2_/g	
				373		3.00 mmol H_2_/g	
				423		2.50 mmol H_2_/g	
	kerogen II-D			323		3.75 mmol H_2_/g	
				373		3.00 mmol H_2_/g	
				423		2.60 mmol H_2_/g	
Raza et al. (2023)^108^	kerogen IA	MD for kerogen GCMC for adsorption	H_2_ (bulk chemical potential)	323	413.68	0.4 mmol/g	Ø = *V*_p_/*V*_t_ = 0.049
				360		0.36 mmol/g	
	kerogen IIA			323		0.45 mmol/g	Ø = 0.056
				360		0.38 mmol/g	
	kerogen IIIA			323		0.70 mmol/g	Ø = 0.076
				360		0.6 0 mmol/g	
	kerogen IIB			323		0.70 mmol/g	Ø = 0.073
				360		0.60 mmol/g	
	kerogen IIC			323		0.65 mmol/g	Ø = 0.075
				360		0.55 mmol/g	
	kerogen IID			323		1.62 mmol/g	Ø = *V*_p_/*V*_t_ = 0.144
				360		1.40 mmol/g	
Abdulelah et al. (2023)^110^	Illite	GCMC	pure H_2_	273	400	0.074 g H_2_/cm^3^	slit pore
				373		0.048 g H_2_/cm^3^	1.5 nm
	kerogen II D			273		0.073 g H_2_/cm^3^	
				373		0.047 g H_2_/cm^3^	
	kaolinite			273		0.044 g H_2_/cm^3^	
				373		0.037 g H_2_/cm^3^	
	quartz			273		0.043 g H_2_/cm^3^	
				373		0.032 g H_2_/cm^3^	
	calcite			273		0.043 g H_2_/cm^3^	
				373		0.033 g H_2_/cm^3^	
^111^							
Wang and Chen (2024)^115^	brown coal	GCMC + MD	pure H_2_	293.15	150	1.8–1.5 mmol H_2_/g	slit pore
	bituminous coal			333.15		1.9–1.6 mmol H_2_/g	5.38 nm
	anthracite coal					2.2–1.8 mmol H_2_/g	
				303.15	150	38 kg H_2_/m^3^	0.5 nm
						40 kg H_2_/m^3^	
						38 kg H_2_/m^3^	
						14 kg H_2_/m^3^	1.0 nm
						15 kg H_2_/m^3^	
						14 kg H_2_/m^3^	
						38 kg H_2_/m^3^	2 nm
						40 kg H_2_/m^3^	
						38 kg H_2_/m^3^	
						38 kg H_2_/m^3^	5 nm
						40 kg H_2_/m^3^	
						38 kg H_2_/m^3^	
Raza et al. (2024)^116^	type II-D kerogen	MD for kerogen GCMC for adsorption	pure H_2_	350	20.67–207	0.25–2.2 mmol/g	slit pore 1 nm
						0.5–4.1 mmol/g	2 nm
			equimolar mixtures H_2_/CO_2_/CH_4_/C_2_H_6_/C_3_H_8_			0.0–1.2 mmol/g	1 nm
						1.0–4.0 mmol/g	2 nm
Zhang et al. (2024)^117^	type II-D kerogen	GCMC methods	pure H_2_	333.15	30–180	3.0–14.5 kg H_2_/m^3^	slit pore 2 nm
						2.6–13.0 kg H_2_/m^3^	5 nm
			H_2_/CH_4_ mixtures (50% mol)			1.2–5.0 kg H_2_/m^3^	2 nm
						1.0–5.5 kg H_2_/m^3^	5 nm
			H_2_/CO_2_ mixtures (50% mol)			1.0–3.0 kg H_2_/m^3^	2 nm
						1.0–5.9 kg H_2_/m^3^	5 nm
	montmorillonite		pure H_2_			2.2–13.6 kg H_2_/m^3^	2 nm
						2.2–12.5 kg H_2_/m^3^	5 nm
			H_2_/CH_4_ mixtures (50% mol)			1.2–6.3 kg H_2_/m^3^	2 nm
						1.2–6.3 kg H_2_/m^3^	5 nm
			H_2_/CO_2_ mixtures (50% mol)			1.0–4.0 kg H_2_/m^3^	2 nm
						1.0–6.0 kg H_2_/m^3^	5 nm
Shang et al. (2024)^119^	kaolinite	GCMC + MD	pure H_2_	303	100	(*) 0.00075 mmol/m^2^	1 nm
						(*) 0.0015 mmol/m^2^	5 nm
						(*) 0.0015 mmol/m^2^	10 nm
						(*) 0.0015 mmol/m^2^	15 nm
						(*) 0.0015 mmol/m^2^	20 nm
				303		(*) 0.0015 mmol/m^2^	10 nm
				330		(*) 0.0014 mmol/m^2^	
				360		(*) 0.0013 mmol/m^2^	
				390		(*) 0.0008 mmol/m^2^	
				423		(*) 0.0006 mmol/m^2^	
				303	200	(*) 0.0025 mmol/m^2^	
				303	300	(*) 0.003 mmol/m^2^	
Alafnan et al. (2024)^121^	quartz	GCMC	CH_4_ + H_2_	350	50–234	0.001–0.013 g/cm^3^	1 nm
			all the results are pure H_2_			0.001–0.012 g/cm^3^	8 nm
	calcite					0.001–0.01 g/cm^3^	1 nm
						0.001–0.012 g/cm^3^	8 nm
	kerogen II-D type					0.001–0.011 g/cm^3^	1 nm
						0.001–0.012 g/cm^3^	8 nm
Muther and Kalantari Dahaghi (2024)^124^	hydroxylated quartz	GCMC	pure H_2_	323	500	2.76 mmol/g	slit pore 3 nm
				363		2.50 mmol/g	
				403		2.25 mmol/g	
			90% H_2_ + 10% CO_2_	323.15		2.37 mmol/g	
				363.15		2.20 mmol/g	
				403.15		2.00 mmol/g	
			75% H_2_ + 25% CO_2_	323.15		1.91 mmol/g	
				363.15		1.75 mmol/g	
				403.15		1.15 mmol/g	
			50% H_2_ + 50% CO_2_	323.15		1.28 mmol/g	
				363.15		1.75 mmol/g	
				403.15		1.15 mmol/g	
			90% H_2_ + 10% CH_4_	323.15		2.45 mmol/g	
				363.15		2.20 mmol/g	
				403.15		2.20 mmol/g	
			70% H_2_ + 25% CH_4_	323.15		1.99 mmol/g	
				363.15		1.80 mmol/g	
				403.15		1.65 mmol/g	
			50% H_2_ + 50% CH_4_	323.15		1.30 mmol/g	
				363.15		1.19 mmol/g	
				403.15		1.15 mmol/g	
Wang et al. (2024)^126^	Na–montmorillonite	MD (*NVT*)	H_2_ + CH_4_ (1:1 molar) with 10% of molar fraction of H_2_O	343	no data	maximum relative selectivity	sit pore
						0.0105	3.7 nm
						0.0105	5.2 nm
						0.0105	6.7 nm
						0.0105	8.2 nm
			H_2_ + CH_4_ (1:1 molar) with 50% of molar fraction of H_2_O	343	no data	0.015	3.7 nm
						0.017	5.2 nm
						0.018	6.7 nm
						0.018	8.2 nm
Yang and Jin (2025)^129^	Ca–montmorillonite	GCMC + MD	pure H_2_			density at the center of the slit	slit pore
				293	400	42 mg/cm^3^	10 nm
				323		35 mg/cm^3^	
				353		32 mg/cm^3^	
				308	50	5 mg/cm^3^	10 nm
					200	20 mg/cm^3^	
					400	38 mg/cm^3^	
					600	58 mg/cm^3^	
				308	400	58 mg/cm^3^	1 nm
						42 mg/cm^3^	2 nm
						41 mg/cm^3^	4 nm
						39 mg/cm^3^	10 nm
	Illite			308	400	58 mg/cm^3^	10 nm
	kaolinite					41 mg/cm^3^	
	montmorillonite					39 mg/cm^3^	

a DFT: density functional theory; MD: molecular dynamics, MC: Monte Carlo, GCMC: grand canonical Monte Carlo, GEMC: Gibbs Ensemble Monte Carlo, *V*_p_: pore volume, *V*_t_: simulation cell volume, k: kerogen, MMT: montmorillonite. Hydrogen adsorption in the table is always the maximum value at the lowest temperature and maximum pressure and concentration when a mixture is simulated. It must be noted that (*) means that these values are the excess adsorption with respect to bulk density under the same thermodynamic conditions.

López-Chávez et al. (2020)^105^ conducted theoretical studies based on DFT to investigate H_2_ adsorption on calcite surfaces. They first used DFT calculations to determine the geometry and stability of CaCO_3_ (*T* < 1000 K). Following this, MD simulations were carried out through a harmonic potential to determine the thermodynamic properties of the calcite crystal and its temperature dependence. Subsequently, the adsorption energy of different configurations of 3, 5, and 10 hydrogen molecules was analyzed on the (110) surface, which is the most reactive surface of calcite. These simulations for different numbers of hydrogen molecules show negative energies and smaller distances to the calcite crystal wall (oxygen atom in particular) when the adsorbed hydrogen molecules form hydrogen clusters. This behavior is characteristic of physisorption. Chemisorption occurs at positive energy values and with hydrogen molecules distributed randomly. Another result from the model is that calcite rock has a 0.42% H_2_ storage capacity by mass.

Deng et al. (2022)^106^ explored the adsorption properties of pure CO_2_, pure H_2_, and a CO_2_/H_2_ mixture (in a 1:2 molar ratio) within calcite slit-like pores through grand canonical Monte Carlo (GCMC) and MD simulations. The investigation considered the effects of varying pore sizes (2.5, 3.5, 4.5 nm), pressures (up to 300 bar), and temperatures (300 K < *T* < 340 K) on the molecular behavior of the CO_2_/H_2_ mixture. Both calcite and the CO_2_ molecules were modeled as rigid bodies. As was expected, the findings revealed that CO_2_ consistently exhibited higher adsorption than H_2_. In mixtures, CO_2_ molecules tend to form bilayers when adsorbed onto the pore walls, which is attributed to the strong attraction between the oxygen atoms in CO_2_ and the calcium atoms in calcite. The selective adsorption coefficients of CO_2_ were found to be greater than those of H_2_, with lower pressures favoring increased selectivity toward CO_2_, while temperature exerted a minimal influence on selectivity. Regarding diffusion, the coefficients for H_2_ were consistently higher than those for CO_2_. Among the diffusion coefficients analyzed, the Fick diffusion coefficient was the largest, followed by the Maxwell–Stefan diffusion coefficient, with the self-diffusion coefficient being the smallest for the mixture. Additionally, the diffusion coefficients for H_2_ decreased with increasing pressure, whereas those for CO_2_ increased before stabilizing. All diffusion coefficients increased with rising temperature.

Raza et al. (2022)^107^ performed MD simulations using the polymer-consistent force field plus (pcff++) to stabilize the kerogen type II cell, followed by GCMC simulations to analyze H_2_ adsorption behavior. They investigated each kerogen maturity level (classified into four categories—immature, top of oil window, middle/end-of-oil window, and overmature), with different porosity and carbon content over a wide range of pressures (27.5 to 200 bar) and temperatures (323 to 423 K). The amount of H_2_ adsorbed was directly proportional to the carbon content of kerogen and its porosity. In an extension of the previous work using the same force field and methodology, Raza et al. (2023)^108^ extended the analysis to higher pressure (400 bar) but lower temperature (360 K), examining six other types of kerogens, covering type I through Type III. A larger cell volume was used to avoid problems caused by kerogen macromolecules (Table 5). H_2_ adsorption was greater for overmature kerogens with high aromatic contents. This finding agrees with the existing literature.^109^ The isosteric heat exhibited exactly the opposite trend. We must emphasize that in the presence of CH_4_, the adsorbed mass of H_2_ was lower, although the pressure and temperature dependencies remained the same. For isosteric heat, the results were inconclusive.

Using the GCMC technique, Abdulelah et al. (2023)^110^ simulated the physisorption of H_2_ in different materials, both inorganic and organic, across a range of temperature and pressure. The results were consistent with those of previous experiments and simulations. The different adsorbent solids were ranked from the highest to lowest in terms of storage capacity: Illite, type II-D kerogen, kaolinite, quartz, and calcite.

Chen et al. (2023)^111^ conducted simulations of various CH_4_–H_2_ mixtures, both with and without water, confined within slit-type pores (2–10 nm) using the GCMC technique. The selected temperatures (340, 370, and 400 K) and pressures (100, 300, and 500 bar) resemble those found in gas reservoirs in the United States. The simulation results were consistent with those from previous studies. The influence of the pore size on adsorption remained negligible. In all cases, hydrogen remained in the center of the slit pore as a free gas, except when the hydrogen concentration exceeded 80%. Under these conditions and in the absence of water, an adsorbed layer of hydrogen was observed.

Al-Harbi et al. (2023)^83^ investigated the interaction of hydrogen molecules with a slab surface of silica-clay shale, consisting of 50% silica–50% kaolinite, utilizing DFT to calculate adsorption energy. The interface was built with QuantumATK Virtual NanoLab.^112^ Simulations were performed using Vienna ab initio simulation package 5.4.4,^113^ and H_2_ adsorption was investigated at three different locations: within the kaolinite-dominated region of the interface, within the silica-dominated portion, and directly at the interface. The results showed that H_2_ adsorption is strongest at the interface region, with progressively weaker adsorption observed in the kaolinite and silica regions, as indicated by the corresponding average adsorption energies of −0.14, −0.05, and −0.035 eV, respectively.

Ho et al. (2023)^114^ used metadynamics MD simulations in LAMMPS to calculate the free energy landscape (potential of mean force) of H_2_ intercalation into swelling MMT interlayers. They found that H_2_ intercalation was thermodynamically unfavorable, with accumulation near hydrophobic sites being slightly less unfavorable than that near hydrophilic sites. The solubility of H_2_ in confined water within the interlayers was comparable to that in pure water. These results suggest that H_2_ loss due to adsorption into this porous caprock material or leakage through interlayers in H_2_ geological storage may be minimal.

Using a hybrid GCMC and MD technique, Wang and Chen (2024)^115^ analyzed the adsorption of H_2_ on brown, bituminous, and anthracite coal with up to 10% water by weight, at different pressures and temperatures. The effect of slit pore size ranging from 0.5 to 5.38 nm was also analyzed. The results were in qualitative agreement with those of previous experiments and simulations. However, there was a significant difference; the smallest pores exhibited adsorption almost 4 times greater than the larger pores. This effect was observed consistently across all of the coal types studied.

As depleted shale gas wells are promising candidates for H_2_ storage, Raza et al. (2024)^116^ used GCMC simulations to investigate the adsorption of H_2_ in type II-D kerogen in the presence of CH_4_, C_2_H_6_, C_3_H_8_, and CO_2_ within slit-type pores of 1 and 2 nm. Different temperatures, pressures, and gas mixtures were analyzed, and the results of these simulations were consistent with previous simulation results. H_2_ adsorption was always lower when other gases competed for adsorbent sites on the pore wall. H_2_, whether alone or in binary mixtures, always exhibited self-diffusion coefficients 20 times higher than the other species simulated in this work (>0.025 cm^2^/s). This value decreased by up to half when the pressure was higher than 70 bar in both the 1 nm and 2 nm cases, suggesting that 70 bar represents a pressure threshold for abrupt changes in this dynamic property.

Zhang et al. (2024)^117^ conducted GCMC simulations to study the adsorption of pure H_2_ and mixtures of H_2_/CH_4_ and H_2_/CO_2_ (50% mol) in slit pores (2 and 5 nm) on kerogen type II-D and MMT. To describe H_2_ molecular interactions, a three-site linear charge model was used.^118^ The results showed that pure H_2_ exhibited higher adsorption on kerogen surfaces compared to MMT, and (as expected) the presence of CO_2_ and CH_4_ significantly reduced H_2_ adsorption capacity. This reduction was more significant in pores of 2 nm.

In their study, Shang et al. (2024)^119^ focused on UHS in reservoirs, highlighting the significance of understanding H_2_ adsorption and diffusion in kaolinite slit pores using the ClayFF force field.^120^ Using GCMC and MD simulations, they examined the adsorption of pure H_2_ in slit pores ranging from 1 to 20 nm, at pressures between 10 and 300 bar, and temperatures from 303 to 423 K. The study found that in pores larger than 5 nm, hydrogen primarily remained in the bulk phase, minimizing adsorption-related losses. Furthermore, the mineral composition of the formation influenced H_2_ storage, with H_2_ tending to accumulate more on gibbsite surfaces than on siloxane surfaces due to variations in surface charge properties. As a result, negatively charged formations were determined to be more suitable for UHS.

Alafnan et al. (2024)^121^ used GCMC simulations with the polymer consistent force field to investigate the adsorption behavior of H_2_, CH_4_, and their mixtures within slit-like pores ranging from 1 to 8 nm in three substrates: quartz,^122^ calcite,^123^ and type II-D kerogen.^109^ The findings revealed that while the H_2_ storage capacity was nearly independent of pore size, it was found to be highest in quartz and lowest in calcite. An important conclusion from these simulations was that H_2_ adsorption in organic-rich formations was minimal when residual natural gas was present.

Muther and Kalantari Dahaghi (2024)^124^ simulated the adsorption and Henry coefficients of H_2_, and CH_4_, H_2_O, and CO_2_ mixtures, within a 3 nm hydroxylated alpha quartz slit using the GCMC technique. The CLAYFF force field was applied to describe the interactions between quartz and water molecules, with the water molecule being modeled using the simple-point charge extended (SPC/E) model. H_2_ was modeled using Darkrim et al.’s (1999) model.^125^ The maximum amount of H_2_ adsorbed was approximately 2.7 mmol of H_2_/g of quartz, which occurred at the lowest temperature (323 K) and maximum pressure (500 bar) for a pure hydrogen system. Additionally, as expected, the presence of other components, such as CO_2_, CH_4_, or H_2_O films, led to a significant reduction in H_2_ adsorption.

In a recent study, Wang et al. (2024)^126^ examined the adsorption of an equimolar mixture of H_2_ and CH_4_ in the presence of water, with concentrations ranging from 10% to 50%, using MD simulations. The study was conducted both under thermodynamic equilibrium (*NVT*) and nonequilibrium conditions, with a constant applied force. The simulations were conducted using LAMMPS in Na–montmorillonite slit pores,^120^ with pore sizes ranging from 3.7 to 8.2 nm. It must be remarked that H_2_ was represented by two-site model potential.^127^ They evaluated and plotted the selectivity in H_2_ adsorption, which represents the ratio of H_2_ to CH_4_ adsorbed within the pore, compared to the same ratio in the nonadsorbed phase. These selectivity data are provided in Table 5. The key conclusion was that 10% water concentration, with CH_4_ as a cushion gas, provided nearly optimal conditions for H_2_ storage capacity, while also promoting the self-diffusion of H_2_ for extraction. However, when the water concentration exceeded 50%, a water channel formed at the center of the slit, which reduced the H_2_ storage capacity.

Phan et al. (2025)^128^ employed MD simulations to investigate the effects of CO_2_ and CH_4_ on the wettability of H_2_–brine–clay systems (20 wt % NaCl, 1 wt % KCl) under geological conditions (15 bar and 333 K). Talc and hydroxylated kaolinite served as the clay substrates. The findings indicated that CO_2_ and CH_4_ reduced water wettability in H_2_–brine–talc systems but had no effect on H_2_–brine–kaolinite systems, emphasizing the influence of clay–brine interactions, with talc exhibiting hydrophobic behavior and kaolinite displaying hydrophilic characteristics. No evidence of H_2_ physisorption was observed in any case.

Yang and Jin (2025)^129^ analyzed the H_2_ storage capacity of three clays (Illite, Ca–montmorillonite, and kaolinite) using GCMC and MD simulations within the pressure range of 50–800 bar and temperature range of 293–353 K. For hydrogen molecules, the force field employed was the classical model proposed by Darkrim and Levesque.^130^ The simulations revealed that Illite exhibited the highest H_2_ adsorption capacity due to its stronger binding energy and superior diffusion properties, surpassing MMT and kaolinite. H_2_ storage capacity is enhanced by an optimized slit pore size. Table 5 presents the density of H_2_ within the pores. This value corresponds to the minimum density at the center of the pore, while the density of the adsorbed monolayer was always higher—in some cases, reaching up to twice the minimum value.

### 4.2 Isotherm Modeling Approaches and Calibration

This section focuses on modeling H_2_ sorption using adsorption isotherm models. We begin by summarizing the existing isotherm modeling data reported in the literature, followed by an analysis of experimental equilibrium data utilizing a selection of widely used isotherm models. In another work of ours,^131^ we further incorporated the experimental equilibrium data into a deep learning framework using a physics-informed neural network methodology, which integrates physical laws directly into the training of neural networks.

#### 4.2.1 Literature Data on Adsorption Isotherm Models

The Langmuir model is the most widely applied in the literature, followed by the Freundlich and Toth models. Other models, such as Sips, BET, Redlich–Peterson, and Dubinin–Astakhov, have also been employed but less frequently. As summarized in Table 6, which compiles reported parameters for Langmuir, Toth, and Freundlich models from existing studies, the best-fitting model varies in different studies. As highlighted, many studies have used only the Langmuir model to fit their experimental data, reporting it as sufficient for their systems.^43,73,89,93,103,104^ However, in comparative studies, particularly those involving natural rock samples like shales, other models (such as Freundlich, Toth, BET, and Sips) have outperformed the Langmuir model.^19,24,45,68,72,74,84,85^ This is an expected finding, attributable to the heterogeneous surfaces and potential for multilayer adsorption in such complex materials.

**Table 6:** Summary of H_2_ Adsorption Modeling Studies Using Langmuir (L), Toth (T), Sips (S), and Freundlich (F) Models^a^

				Langmuir		Toth			Freundlich	
refs	sample	*T* (°C)	best fit	*A*_0_ (mmol/g)	*K*_L_ (MPa^–1^)	*A*_0_ (mmol/g)	*K*_T_ (MPa^–1^)	*m*_T_	*k*_F_	1/*n*_F_
Bardelli et al. (2014)^45^	COX_pur_	28	T	1.535	0.36	1.141	0.300	2.714	0.456	0.451
	COX_pur_	90	T	2.170	0.19	1.245	0.230	5.030	0.392	0.602
	COX_raw_	28	T	0.558	0.73	0.463	0.340	10.95	0.261	0.293
	COX_raw_	90	T	0.648	1.63	0.587	0.680	3.254	0.424	0.176
Mondelli et al. (2015)^68^	Na–montmorillonite (0% Fe)	90	T	1.450	0.35	1.090	0.320	2.000	0.402	0.490
	Na–montmorillonite (6% Fe)	90	T	1.470	0.38	1.020	0.300	6.400	0.433	0.460
Wolff-Boenisch et al. (2023)^23^	natural pure montmorillonite	–196	L	0.523	0.807				0.257	0.316
		–78	L	0.493	0.38				0.157	0.455
		30	L	0.180	0.324				0.052	0.451
Wang et al.* (2023)^43^	montmorillonite	0	L	0.261	0.051					
		25	L	0.180	0.039					
		45	L	0.127	0.049					
		75	L	0.037	0.022					
	chlorite	0	L	0.028	0.524					
		25	L	0.023	0.262					
		45	L	0.027	0.120					
		75	L	0.024	0.050					
	sepiolite	0	L	1.715	0.044					
		25	L	2.678	0.021					
		45	L	2.805	0.019					
		75	L	5.358	0.007					
	palygorskite	0	L	1.047	0.043					
		25	L	1.285	0.025					
		45	L	1.725	0.017					
		75	L	14.158	0.001					
Ghosh et al. (2023)^72^	hydrophilic bentonite (BEN-1)	–196	F	0.082	16,015	0.002	54.66	2.584	0.015	0.527
		40	T	0.218	39.674	0.009	30.59	1.482	0.050	0.521
	hydrophilic bentonite (BEN-2)	–196	T	0.324	84,314	0.005	25.07	0.429	0.042	0.635
		40	F	0.096	3.451	0.037	32.98	2.718	0.069	0.176
	acid-treated montmorillonite (MMT_1)	–196	T	0.933	67,460	0.030	21.78	0.849	0.152	0.574
		40	S	0.328	16.496	0.055	19.24	1.273	0.133	0.362
	acid-treated montmorillonite (MMT-2)	–196	T	1.004	69,355	0.027	21.21	0.573	0.160	0.578
		40	F	0.420	8.609	0.168	13.64	0.999	0.226	0.267
Zhang et al.* (2024)^73^	montmorillonite	0	L	0.085	0.244					
		10	L	0.100	0.120					
		20	L	0.087	0.097					
		30	L	0.071	0.096					
		40	L	0.068	0.085					
		50	L	0.061	0.068					
	sepiolite	0	L	1.314	0.084					
		10	L	1.228	0.082					
		20	L	1.206	0.078					
		30	L	1.073	0.074					
		40	L	1.012	0.067					
		50	L	0.953	0.064					
Masoudi et al. (2025)^74^	Hekkingen	50	F	22,040	2.34 × 10^–7^	1.326	0.004	4.704	1.10 × 10^–3^	1.767
	Draupne	50	F	7083	2.92 × 10^–7^	0.971	0.002	3.849	1.06 × 10^–3^	1.335
	Rurikfjellet	50	F	6201	3.45 × 10^–7^	1.000	0.002	3.880	1.09 × 10^–3^	1.339
	Agardhfjellet	50	F	22,178	2.05 × 10^–7^	1.262	0.004	4.583	2.95 × 10^–3^	1.215
	Hekkingen_dry	50	F	0.267	0.038	-	-	-	1.12 × 10^–2^	0.821
	Draupne_dry	50	F	11,395	2.34 × 10^–7^	1.135	0.002	4.344	1.76 × 10^–3^	1.207
	Rurikfjellet_dry	50	F	16,765	1.83 × 10^–7^	1.162	0.003	4.435	1.72 × 10^–3^	1.283
	Agardhfjellet_dry	50	F	6712	3.14 × 10^–7^	1.023	0.002	4.068	9.74 × 10^–5^	2.468
	smectite	50	F	0.595	0.007	0.073	0.056	2.835	3.96 × 10^–3^	0.997
	montmorillonite	50	F	21,243	2.65 × 10^–7^	1.323	0.004	4.688	3.08 × 10^–3^	1.295
	Smectite_dry	50	F	18,839	2.32 × 10^–7^	1.291	0.003	4.659	3.15 × 10^–3^	1.161
	Montmorillonite_dry	50	F	0.635	0.174	1.263	0.139	0.540	0.108	0.588
Wang et al. (2024)^19^	Longmaxi formation shale	30	F	0.146	1.316				0.084	0.210
Wang et al. (2024)^85^	Chang 7 shale (sample 412)	25	F	0.095	0.043				0.001	1.298
		45	F	0.094	0.039				0.001	1.310
		65	F	0.060	0.060				0.001	1.402
	sample 413	25	F	0.171	0.027				0.001	1.159
		45	F	0.154	0.026				0.001	1.167
		65	F	0.134	0.028				0.001	1.337
	sample 427	25	F	0.172	0.026				0.001	1.167
		45	F	0.136	0.033				0.001	1.161
		65	F	0.116	0.033				0.001	1.183
Alanazi et al. (2025)^84^	Jordanian source rock (JO-1)	–80	B	0.538	1.751				0.355	0.192
		0	B	0.250	0.661				0.107	0.357
		30	B	0.150	1.134				0.084	0.253
		60	B	0.256	0.101				0.026	0.737
	JO-2	–80	B	1.519	0.166				0.240	0.649
		0	B	0.634	0.263				0.150	0.537
		30	B	0.282	0.597				0.113	0.386
		60	B	0.145	1.120				0.079	0.279
Liu and Liu* (2023)^89^	lignite type A (LigA) from Beulah Seam, USA	30	L	0.050	0.177					
	sub-bituminous type B (SubB) from Rosebud Seam	30	L	0.450	0.032					
	sub-bituminous type A (SubA) from Deadman Seam	30	L	0.760	0.027					
	high-volatile type C bituminous (HvCb) from Illinois #6 Seam	30	L	0.700	0.024					
	high-volatile type B bituminous (HvBb) from Kentucky #9 Seam	30	L	0.450	0.049					
	low-volatile bituminous (LvB) from Pocahontas #3 Seam	30	L	1.170	0.031					
	fresh semianthracite (SemiAn) from Goodspring #3 Seam	30	L	0.820	0.066					
	anthracite (An) from Lykens #2 Seam	30	L	0.950	0.072					
Li et al.* (2024)^93^	cretaceous Cameo coal samples, collected from outcrops in Colorado (TOC: 72.2%)	30	L	1.170	0.028					
		45	L	1.120	0.024					
		60	L	1.100	0.020					
Mirchi and Dejam (2023)^24^	oil-wet sandstone	25	F,S	16.646	0.000				0.001	1.692
Wang et al.* (2024)^103^	deep purple mudstone	25	L	0.051	0.044					
		45	L	0.040	0.050					
		65	L	0.047	0.035					
	gray–green mudstone	25	L	0.068	0.032					
		45	L	0.128	0.012					
		65	L	0.296	0.005					
	gray sandstone	25	L	0.068	0.031					
		45	L	0.050	0.041					
		65	L	0.063	0.027					
Wang et al.* (2024)^104^	diatomaceous earth	25	L	0.450	0.042					
		45	L	0.376	0.039					
		65	L	0.473	0.024					

a The studies highlighted by an asterisk (*) only used the Langmuir equation for fitting their experimental isotherm data.

#### 4.2.2 Modeling Ranking and Calibration

In this section, the experimental equilibrium data were analyzed using a selection of commonly employed adsorption isotherm models

1 $$Langmuir:\;A=\frac{A_{0}k_{L}P}{1+k_{L}P}$$

2 $$Toth:\;A=\frac{A_{0}k_{T}P}{{\left[1+{\left(k_{T}P\right)}^{n_{T}}\right]}^{\frac{1}{n_{T}}}}$$

3 $$Sips:\;A=\frac{A_{0}{\left(k_{S}P\right)}^{\frac{1}{n_{S}}}}{1+{\left(k_{S}P\right)}^{\frac{1}{n_{S}}}}$$

4 $$Redlich-Peterson\;\left(RP\right):\;A=\frac{aP}{1+cP^{d}}$$

##### 4.2.2.1 Maximum Likelihood Model Calibration and Identification Criteria

We use formal model selection criteria to assess, in a relative sense, the ability of each selected model to interpret the available sorption measurements. Specifically, we use Bayesian information criterion (BIC),^132^ Akaike information criterion (AIC),^133^ and corrected AIC (AICc),^132^ which are based on the maximum likelihood framework. These criteria enable the ranking of model formulations by balancing the goodness-of-fit of the models with their complexity. Importantly, they adhere to the principle of parsimony, favoring models with fewer parameters when the explanatory power is comparable.

They are defined as follows

5 $$BIC=NLL+N_{p}\;\ln\left(N_{c}\right)$$

6 $$AIC=NLL+2N_{p}$$

7 $$AICc=NLL+2N_{p}+\frac{2N_{p}\left(N_{p}+1\right)}{N_{c}-N_{p}-2}$$

where *N*_c_ is the number of data points, *N*_p_ is the number of model parameters, and NLL is the negative log-likelihood criterion, defined as

8 $$NLL=\frac{J}{\sigma_{Y}^{2}}+N_{c}\;\ln\left(2\pi \sigma_{Y}^{2}\right)$$

where *J* is the sum of the squares of residuals and σ_Y_^2^ is the variance of the residuals.

Posterior model weights derived from the BIC, AIC, and AICc criteria are presented in the Supporting Information (Tables S1–S3). They consistently indicate that the Sips model provides the most reliable interpretation of the sorption isotherms across all examined rock types. For clay samples, the Sips model shows the highest degree of support among the tested models. The same preference is observed for shale and coal samples, where Sips also ranks as the most suitable model. Depending on the rock type, the RP and Toth models emerge as secondary choices, demonstrating relatively strong but less consistent performance compared to Sips.

## 5 Critical Synthesis and Factors Influencing Hydrogen Adsorption Behavior

In this section, we present a critical synthesis of the current literature and analyze the key quantifiable parameters affecting the H_2_ sorption capacity in natural porous materials. This assessment is based on the experimental data gathered from the literature presented in Section 3. It is important to note that the experimental tests in different studies were conducted at varying temperatures and pressures, both of which influence the sorption capacity. To compare the effects of different parameters accurately, we standardize the conditions to match the most frequently reported conditions, which are

- 25 °C and 100 bar for cross-lithological parameters
- 25 °C and 100 bar for clay minerals
- 60 °C and 100 bar for shales
- 30 °C and 100 bar for coal samples
- 25 °C and 100 bar for other samples

If equivalent data for certain data sets were not available under these specific conditions, we employed alternative methods to estimate the equivalent values. First, we used the plateau amount if the sorption curve had already reached a plateau under these conditions. Second, we applied the suggested models (e.g., Langmuir and Sipp) from the respective studies to extrapolate the data. If neither of these approaches was applicable, the data were not included.

We classified the influencing parameters into three main categories: thermodynamic and environmental parameters, textural parameters, and structural/mineralogical parameters. It is important to note that not all structural or mineralogical analyses are applicable across all of the material groups. Accordingly, due to both data limitations and differences in relevance among sample categories, we present a targeted evaluation of the key factors within each specific material type.

### 5.1 Thermodynamic/Environmental Parameters

Thermodynamic and environmental factors (including pressure, temperature, and hydration) universally influence the H_2_ adsorption behavior. The effects of these parameters are generally consistent across material types and align with established theoretical and experimental trends.

As anticipated (see Section 2), thermodynamic evidence consistently indicates weak physisorption, confirming that van der Waals interactions dominate over chemisorption. The uptake of H_2_ decreases with increasing temperature, reflecting the exothermic nature of the physisorption. Nonetheless, Bardelli et al. (2014)^45^ reported a minor but direct correlation between adsorption and temperature. This might be due to either experimental artifacts or specific secondary reactions.

Pressure effects are also predictable: At low pressures, H_2_ adsorption follows Henry’s law (adsorbed amount increases linearly with pressure), while at higher pressures, the behavior conforms to one of the classical isotherms as per IUPAC classification. Some materials reach a plateau in adsorption (saturation), while others do not. In Tables 1–4, samples exhibiting a defined plateau are specifically highlighted.

Most of the atomistic simulations in Section 4.1 reported the temperature and pressure trends as one of their main results: H_2_ adsorption is directly proportional to the pressure and inversely proportional to the temperature.

A noteworthy consideration is hydration; except for Masoudi et al. (2025),^74^ all other experimental studies on H_2_ sorption–desorption in natural rocks have been conducted on dry samples. Although Masoudi et al.’s study lacked control over moisture content, it provides valuable insights into the significance of considering moisture content in H_2_ sorption studies. Unlike the dry conditions often studied, the rock samples studied in their natural state within geological formations are not dry. In fact, the rock matrix, especially in deep aquifers considered for H_2_ or CO_2_ storage, is water-saturated in shallower regions and brine-saturated in deeper sections. In such environments, water molecules can occupy or block pores and compete with H_2_ molecules for sorption sites on the surface.^134–141^ As a result, the reported sorption capacities at dry conditions likely represent the maximum potential, as they do not account for the complexities found in natural geological environments.^45,68,74,137,139,141–143^

As was explained in Section 4.1, the effect of hydration has been studied in a few molecular simulation studies.^111,124,126^ They showed that increasing water content in modeling decreases H_2_ adsorption and sometimes leads to the formation of blocking water channels, further limiting adsorption sites.

### 5.2 Textural Parameters

Textural parameters, such as specific surface area, (micro)­pore volume, and average pore diameter, represent cross-lithological descriptors. Their reported impact varies across studies. When feasible (i.e., with multisample data available), (micro)­pore volume and total surface area are typically identified as primary controls. An exception is the data of Alanazi et al. (2023),^21^ who observed a clear inverse relationship for shales. No discernible correlation between micropore volume and BET surface area (derived from nitrogen adsorption isotherms) with H_2_ adsorption was observed in the data sets of Arif et al. (2022),^16^ Tao and Ju (2025),^95^ and Han et al. (2025)^96^ for coal, Masoudi et al. (2025)^74^ for shale, and Ziemiański and Derkowski (2022)^69^ for clay minerals. However, Ziemiański and Derkowski (2022)^69^ did observe a significant correlation between the micropore volume derived from CO_2_ adsorption isotherms and H_2_ adsorption (as was explained in Section 3). The same pattern can be seen in the data reported by Han et al. (2025),^96^ where a clear positive correlation can be seen between the parameters obtained from CO_2_ adsorption isotherms (specific surface area and total pore volume) and H_2_ adsorption.

It is important to point out that such a poor correlation is predominantly reported in shales and coals. Although textural parameters are generally thought to correlate with gas adsorption, the complexity of natural systems means that additional influences, including structural and mineralogical parameters such as total clay or organic content, may exert greater control and explain observed data dispersion. These aspects will be explored in detail in subsequent lithology-specific analyses.

The influence of the average pore size is generally less pronounced. The prevalence of micropores (reflected by a small average pore diameter) is more critical than the mean pore size itself. Total pore volume matters more than average pore size; specific micropore characteristics (not adequately captured by average values) dominate the sorption process. Thus, the average pore diameter alone is not a reliable predictor of adsorption performance; detailed micropore characterization is required.

Analyzing the experimental data reveals the same trend. Figure 5 displays the effect of textural parameters on the H_2_ adsorption. Textural properties exhibit a positive correlation with sorption capacity. There are more data available for the BET surface area, which varies greatly among samples (ranging from 0.05 to 273 m^2^/g).

**Figure 5:**
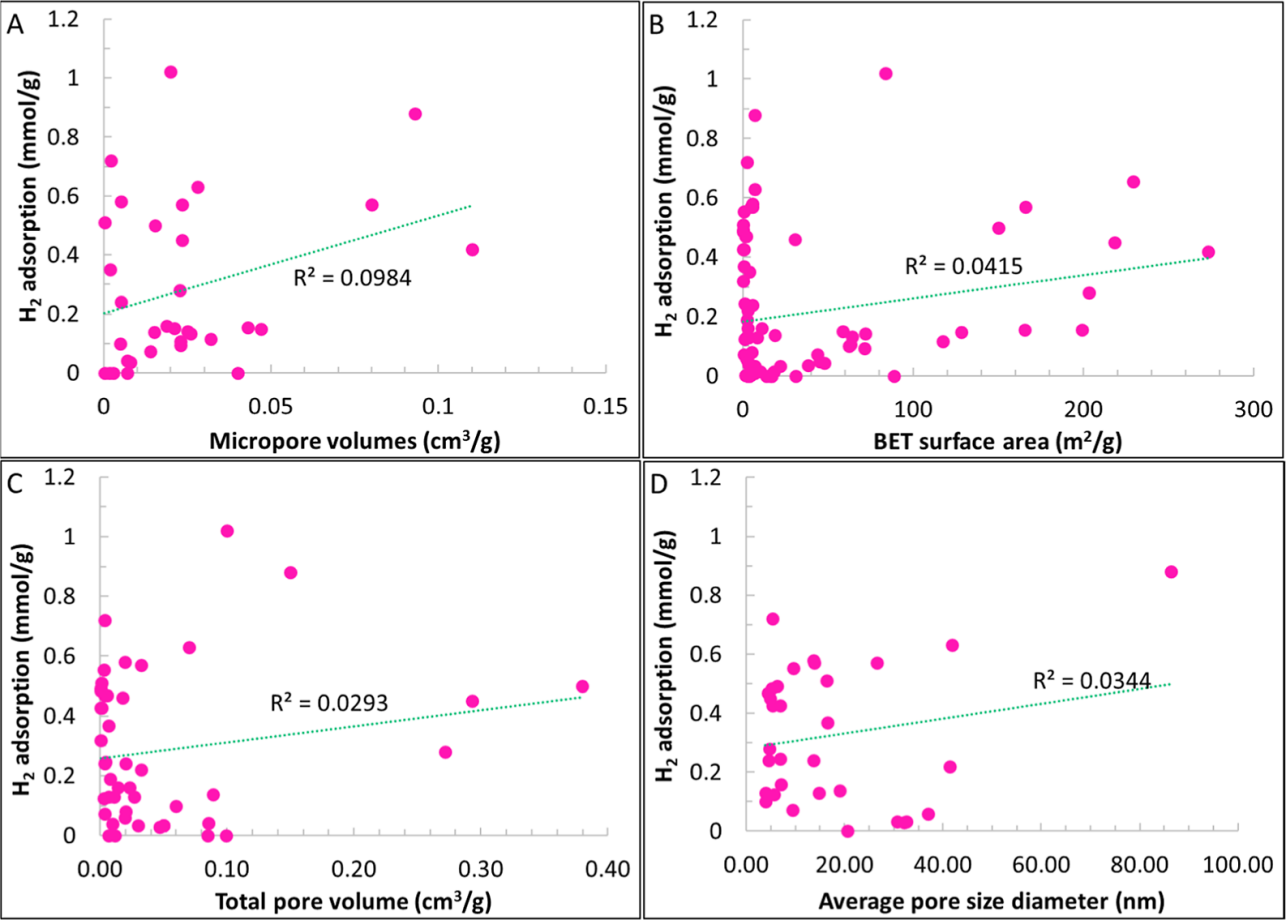
Effect of textural parameters on H_2_ adsorption for all of the samples.

We also analyzed the textural parameters for different sample categories. We categorized clay samples based on the type of clay mineral, as the mineral type appears to be the most crucial factor in determining the H_2_ sorption capacity. Figure 6 shows the effect of the textural parameters.

**Figure 6:**
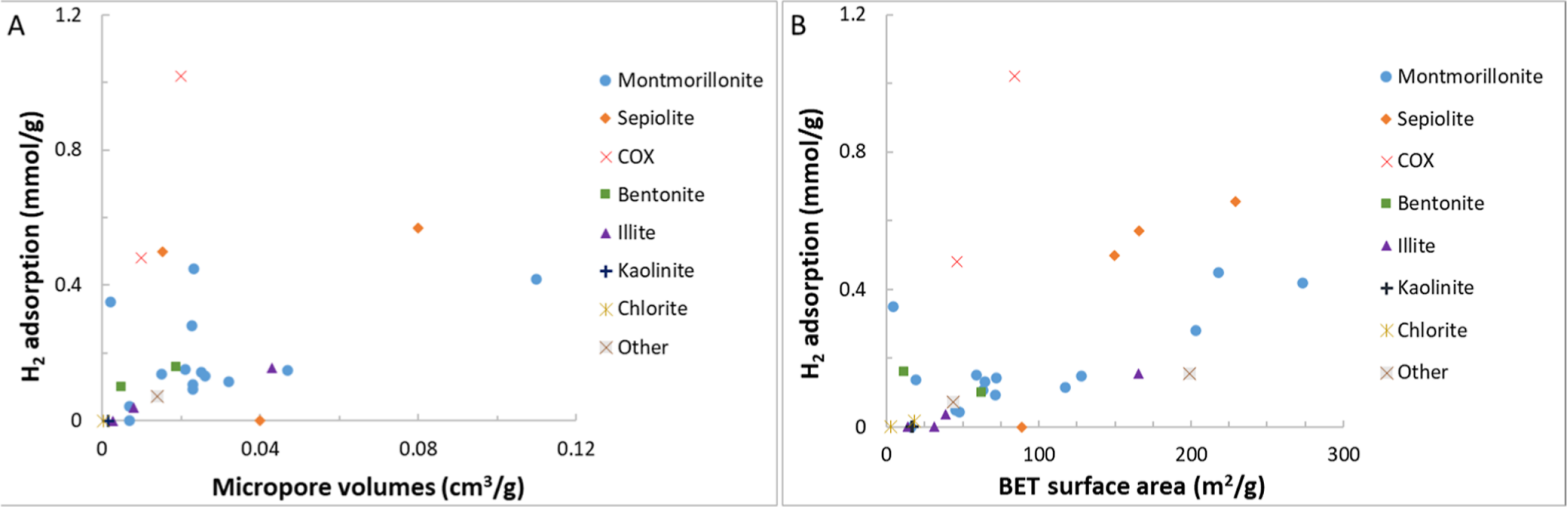
BET surface area and microporous volume vs H_2_ sorption for clay minerals.

To explore the influencing parameters for clayey rocks, we categorized them into three groups: shale gas and oil reservoirs, caprock formations, and shallow siliciclastic mudstone. The “shallow mudstone” category is based on the study by Wang et al. (2024),^85^ which investigated the Triassic Chang 7 Shale Member in the Ordos Basin. Their samples, composed of mudstone, were collected from depths of 223 to 228 m. As shown in Figure 7, there is no clear or consistent correlation between the BET surface area, pore volume, average pore diameter, and H_2_ sorption capacities in these lithologies. As discussed previously, this observation reflects the complexity of these systems: in shales and mudstones, mineralogical and structural attributes exert a stronger influence over sorption behavior than do textural parameters alone. Factors such as clay type, organic content, and mineral matrix are often more decisive, overshadowing the expected influence of surface area or pore metrics.

**Figure 7:**
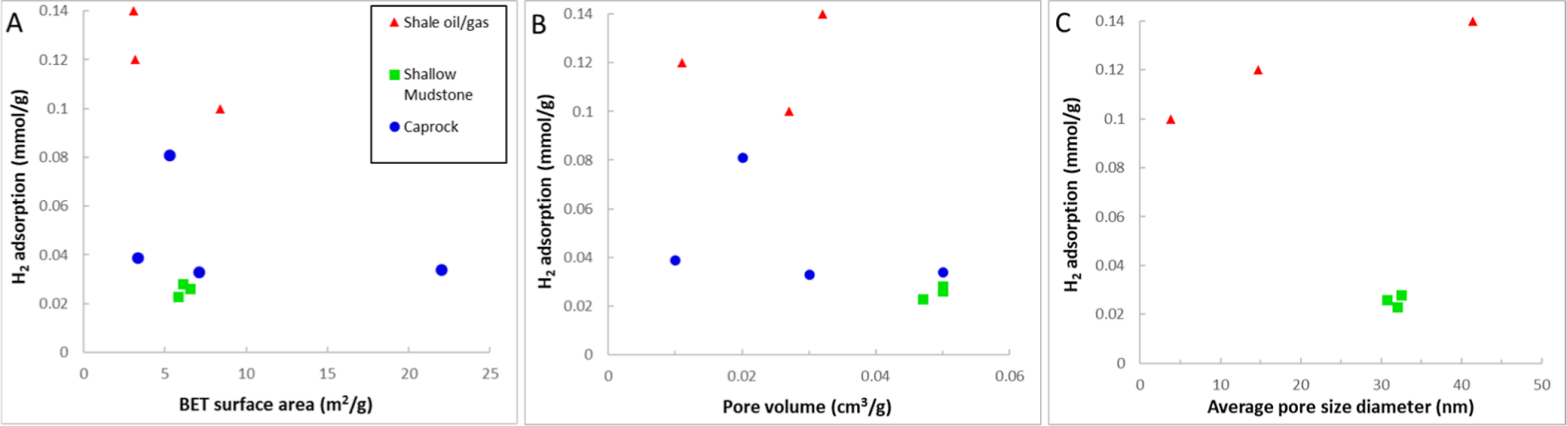
Effect of textural parameters on H_2_ sorption in clayey rocks.

For coal, more data are available. As shown in Figure 8, textural parameters exhibit a moderately positive relationship with H_2_ sorption.

**Figure 8:**
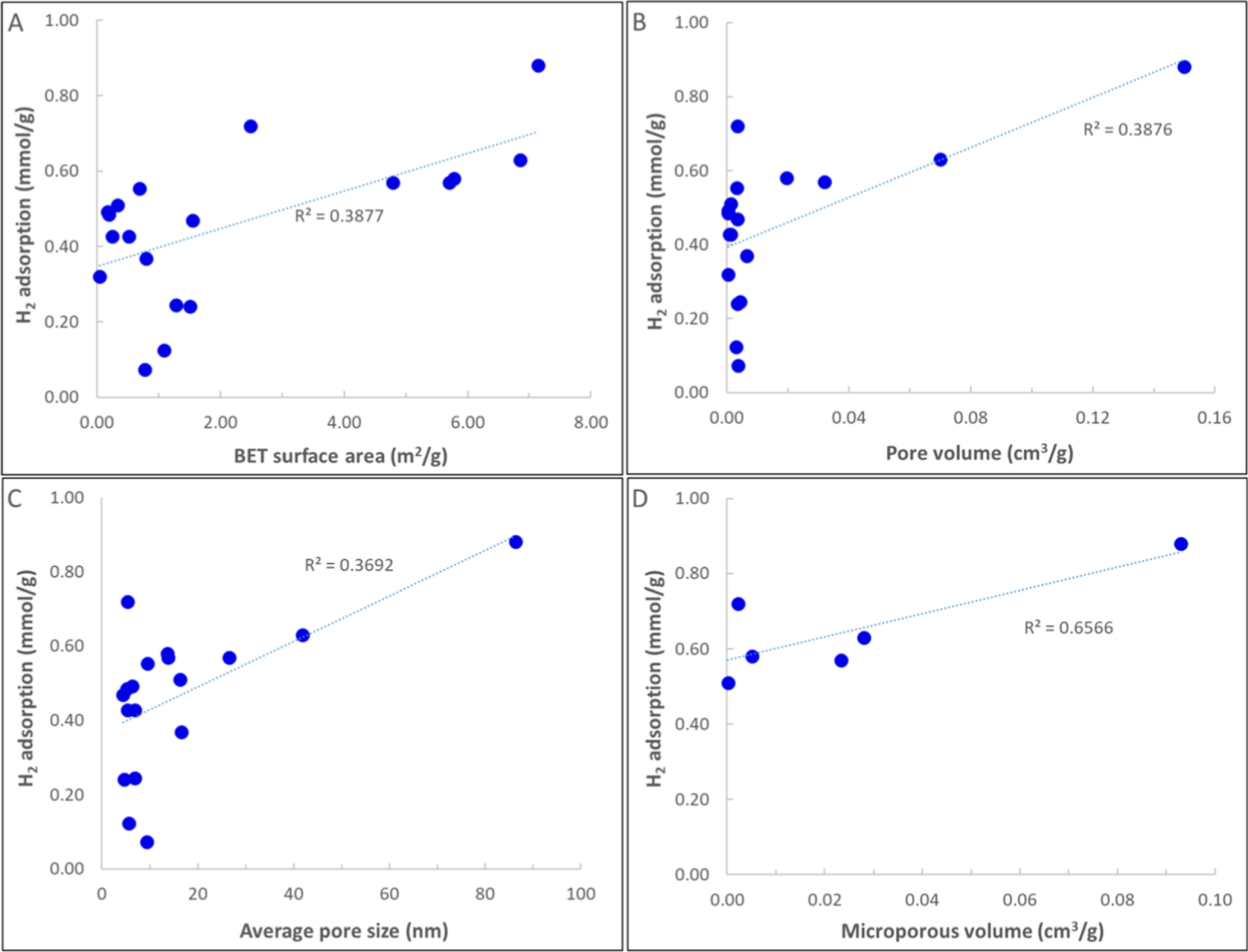
Effect of textural parameters on H_2_ sorption in coal.

### 5.3 Structural/Mineralogical Parameters

The type and structure of the host materials emerge as the most influential factors governing the H_2_ adsorption capacity. Although direct comparison is not feasible due to variations in experimental design, pressure–temperature conditions, and sample preparation, a general hierarchy among the most studied lithologies is evident:

Sepiolite > MMT > high-rank coals ≥ organic-rich shale ≫ sandstone/quartz/carbonate ≥ kaolinite/Illite

The experimental results usually showed very minimal/negligible H_2_ adsorption for quartz, carbonates, kaolinite, and Illite (under the relevant high-pressure, high-temperature conditions for UHS). However, it should be noted that large discrepancies are reported even among similar materials; for instance, the adsorption capacity for MMT ranges between 0 and 1 mmol/g according to cited sources. Reproducibility is one of the biggest issues in this field (see Section 7.1).

#### 5.3.1 Clay Minerals

For clay minerals, most studies consistently demonstrate that sepiolite and MMT are the most H_2_-adsorptive clays, whereas Illite, chlorite, and kaolinite show minimal uptake. This hierarchy reflects structural differences, with fibrous sepiolite’s high microporosity and MMT’s expandable interlayers providing superior H_2_ access compared to nonswelling 1:1 and 2:1 clays.

MMT is the most frequently studied clay mineral, even via MD or MC techniques;^69^ however, their H_2_ uptakes vary dramatically. These differences stem from varying pretreatment protocols, measurement uncertainties, and sample purity/sources. A wide variety of MMT samples have been used by different researchers, undergoing treatments such as thermal treatment at various temperatures, cation exchange, and acid treatment. These treatments affect the structural properties of clay minerals (e.g., basal spacing and surface charge) and, therefore, their sorption capacity. This has been specifically noted in the study by Ziemiański and Derkowski (2022),^69^ which stands out as a significant contribution to the field. They controlled the interlayer water content and spacing by drying the samples at temperatures ranging from 40 to 315 °C. This enabled them to analyze how structural and textural factors affect H_2_ sorption in clay minerals and quantify their impact on total H_2_ sorption. They emphasized the importance of “structurally controlled interlayer adsorption”.

Only four of the clay studies reported desorption data. Ziemiański and Derkowski^69^ reported no hysteresis. From the data reported by Masoudi et al. (2025),^74^ hysteresis is more pronounced for untreated samples compared to the dried ones. Zhang et al. (2024)^73^ stated that hysteresis is more pronounced in swelling clays (MMT) and is weak in microfibrous (sepiolite). Such a trend can be seen in Ruiz-García et al. (2013)^67^ data, where MMT showed more hysteresis compared to the minimal hysteresis of sepiolite.

#### 5.3.2 Shale and Mudstones

For shales and mudstones, the amount of data available to reach definitive conclusions is very limited. Our interpretations are based on the best available data and will likely require revision as more comprehensive and higher-resolution data sets become available in the future.

As shown in Figure 9, the carbonate content and quartz content do not seem to have any meaningful correlations. However, the feldspar content demonstrates a negative correlation with H_2_ sorption. The effect of TOC varies depending on the clay content of the samples. In low clay content samples (shale oil and gas), H_2_ sorption increases with higher TOC levels. It appears that organic matter provides most of the sorption sites for these samples. Conversely, in samples with high clay content, there is a weak or negligible correlation between sorption and TOC. Nonetheless, in these high clay content samples, a positive correlation exists between the clay content and H_2_ sorption, which becomes even more pronounced when considering the combined effect of TOC and clay content.

**Figure 9:**
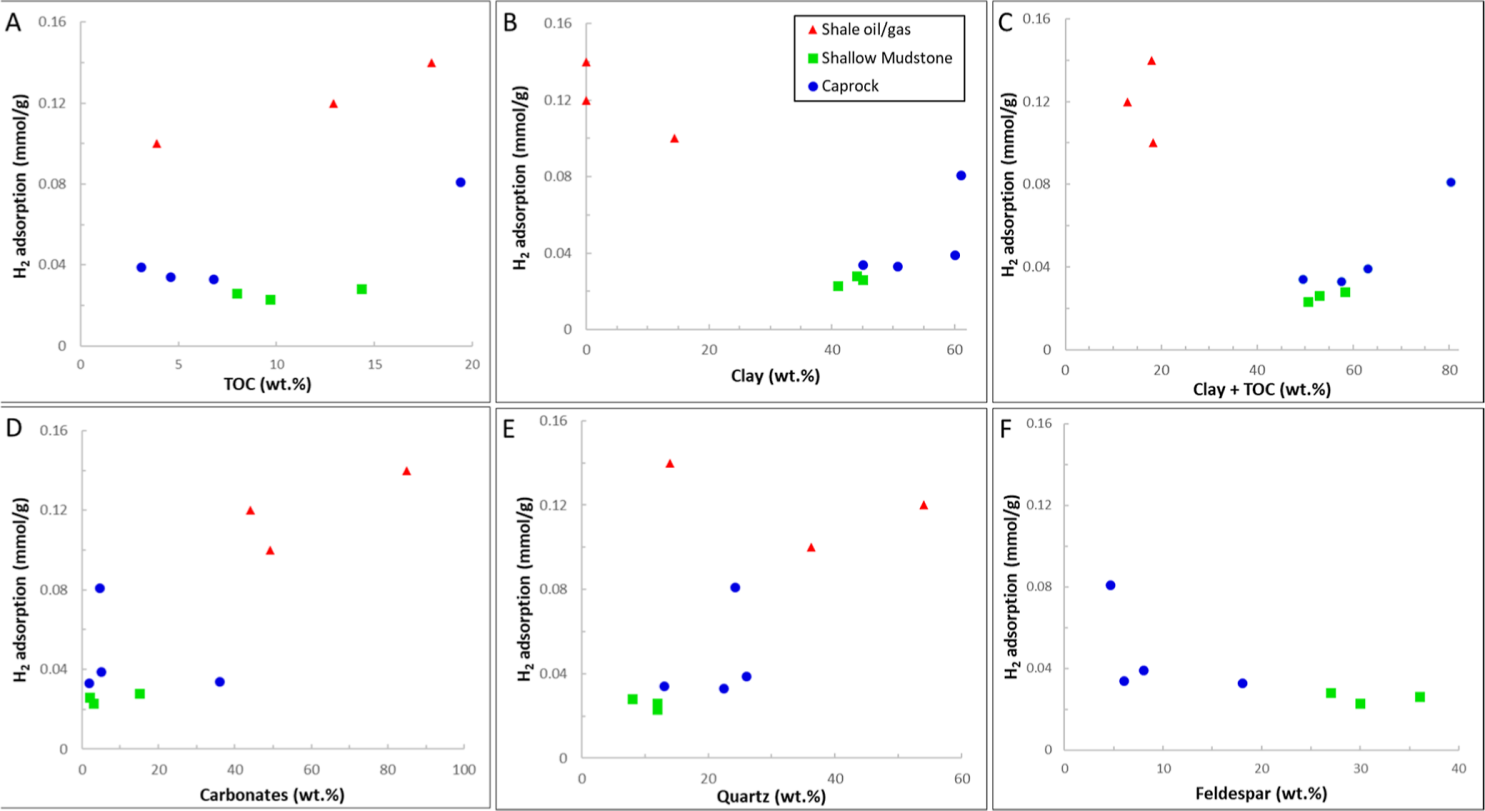
Effect of mineralogical parameters on H_2_ sorption in shales.

It is important to note that the organic matter type and maturity, as well as clay mineralogy, are likely to influence these trends. Most samples in Figure 9 contain low-maturity Type I and II kerogen, except for some high-maturity caprock samples from Masoudi et al. (2025).^74^ While these studies report organic matter maturity levels, none specifically address its impact on H_2_ sorption. For CH_4_, sorption capacity typically increases with organic maturity (within the range of vitrinite reflectance, *R*_O_ < 3, so not for overmature organic matters).^144^ In the context of H_2_ sorption, the data are too sparse to determine whether similar trends apply for shales. Moreover, the diversity in clay content and clay mineral types adds further complexity, making it difficult to isolate the individual contributions of organic matter and minerals to H_2_ sorption.

Several review articles^141,144–147^ highlight that increased TOC levels generally lead to higher gas sorption in shales, a trend supported by numerous studies cited within these reviews. However, a few studies have reported weak or negligible correlations between sorption and TOC in shale samples characterized by very low TOC and high clay content.^145,148,149^ These studies indicate a positive correlation between a high clay content and gas sorption. It is worth noting that these studies are about CH_4_ and CO_2_ adsorption due to the historical emphasis on natural gas exploration and, recently, CO_2_ storage in the energy industry. While these gases differ in certain aspects from H_2_, they can still provide valuable insights and lay the foundation for understanding and predicting the sorption behavior of H_2_ on different minerals.

#### 5.3.3 Coal

For coal, a greater number of parameters were reported, allowing for a comprehensive comparison of their effects on H_2_ sorption. Figure 10 highlights the impact of proximate analysis parameters (moisture, volatile matter, fixed carbon, and ash) on H_2_ sorption in coal. Volatile matter and fixed carbon content appear to have stronger correlations with H_2_ sorption. Fixed carbon shows a positive correlation, volatile matter and ash display negative correlations, and inherent moisture exhibits a weak negative correlation. This aligns with observations of sorption for other gases in coal^150–152^ since moisture, volatile matter, and ash can hinder sorption by blocking pores or reducing the available surface area.

**Figure 10:**
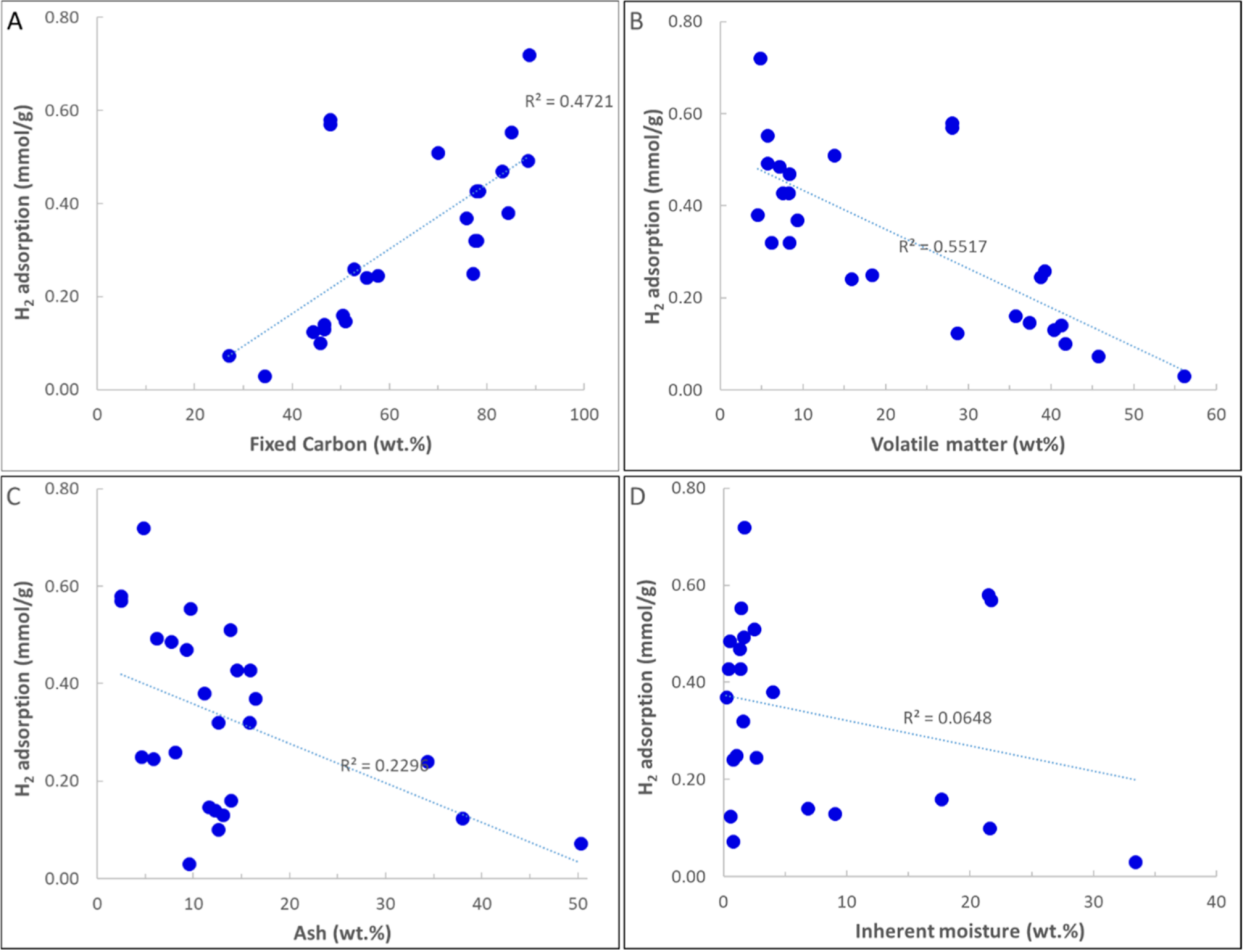
Impact of proximate analysis parameters (moisture, volatile matter, fixed carbon, and ash) on H_2_ adsorption in coal.

Vitrinite reflectance (*R*_O_ %), a measure of coal maturity, shows a positive correlation with H_2_ sorption (Figure 11). As *R*_O_ % increases, indicating higher coal rank and greater structural ordering, the coal develops more micropores and a more condensed aromatic structure,^153^ both of which enhance its capacity to physically sorb H_2_ molecules within its pore network.

**Figure 11:**
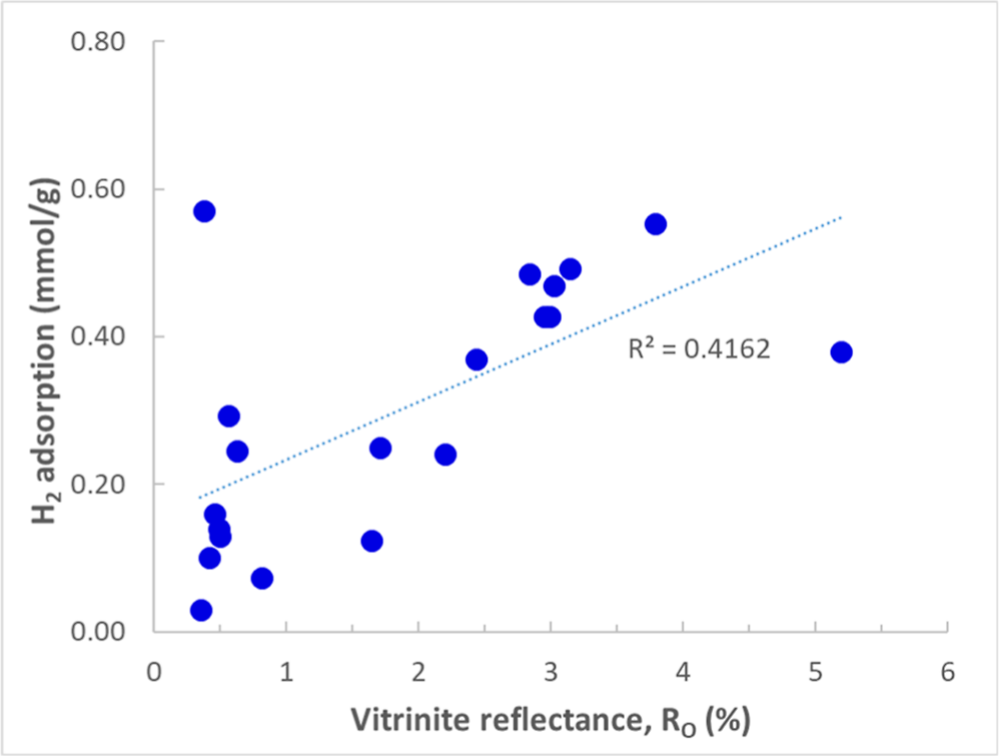
Impact of vitrinite reflectance (*R*_O_ %) on H_2_ sorption in coal.

The plots illustrating the influence of the elemental composition (carbon, hydrogen, nitrogen, oxygen, and sulfur, derived from ultimate analysis) and the relative proportions of the primary maceral groups (vitrinite, liptinite, and inertinite) on H_2_ adsorption in coal are provided in the Supporting Information (Figures S1 and S2). No statistically significant correlations are observed between these compositional parameters and the H_2_ adsorption capacity in coal samples. Due to the weak and inconsistent correlations, it remains challenging to draw definitive conclusions regarding their impact on H_2_ adsorption behavior.

## 6 Practical Implications of Sorption

This section evaluates the implications of H_2_ sorption for various applications, focusing on its potential advantages, disadvantages, and overall significance. Drawing on the extensive literature review conducted in this work, we critically assess whether H_2_ sorption is beneficial or detrimental to specific processes. Additionally, we discuss the practical significance of sorption and whether it should be a key consideration in the design and operation of the discussed systems.

### 6.1 UHS in Porous Media

UHS can be implemented in various geological formations, including aquifers, depleted oil and gas reservoirs, CBM systems, salt caverns, and lined or unlined caverns. In nonporous systems such as salt caverns, lined rock caverns, or hard rocks (e.g., granite), H_2_ sorption is typically minimal due to the low surface area and nonporous (or very low porous) nature of the confining materials (e.g., salt, steel, or dense rock). As a result, this section focuses primarily on UHS in porous media, where sorption plays a more significant role in H_2_ storage and recovery.

That said, experimental data on H_2_ sorption in nonporous formations, such as salt or hard rocks, could provide valuable insights to confirm the negligible sorption capacity of these materials.

In UHS systems, such as aquifers, depleted oil and gas reservoirs, and CBM formations, the presence of a caprock is essential to prevent gas migration. H_2_ sorption at the reservoir–caprock interface can partition H_2_ between adsorbed and free phases. While sorption may temporarily immobilize some H_2_ molecules by attaching them to the rock surface, the high diffusivity of H_2_ means that sorption alone is unlikely to substantially reduce H_2_ leakage through caprocks.

In a caprock, H_2_ may exist in two forms: (i) as dissolved gas in the aqueous phase and (ii) as a gas phase. In water-saturated conditions, the sorption of dissolved H_2_ is typically negligible due to the low H_2_ solubility in water and strong water–rock interfacial interactions (because the rock surface is hydrophilic). The gas phase forms only if the pressure exceeds the capillary entry pressure of the caprock pores, leading to partial desaturation. Under such conditions, some H_2_ molecules might adhere to the rock surface through gas-phase sorption. The sorption of free-phase H_2_ may locally reduce the pore pressure by transferring gas molecules to the adsorbed phase; however, this effect is localized and transient, and the injection pressure maintained at the wellhead dominates the caprock pressure regime. The long-term sealing capacity of a caprock is controlled primarily by its permeability, mineralogy, and capillary properties rather than H_2_ sorption. The small fraction of H_2_ immobilized through sorption may slightly reduce recoverability but has a negligible impact on caprock integrity.

However, it should be noted that gas sorption can induce swelling in certain materials, as observed with CO_2_ in coals, which can reduce permeability.^154–156^ However, H_2_-specific data remain limited. Iglauer et al. (2022) observed no swelling or permeability reduction in deep coal seams during H_2_ injection.^157^ In contrast, Liu and Liu (2024)^94^ reported matrix shrinkage in coal due to H_2_ adsorption, as opposed to CH_4_ and CO_2_, which induced matrix swelling. These contradictory findings highlight the need for further testing, especially in clay-rich lithologies where swelling/shrinkage behavior could impact caprock integrity.

In the reservoir itself (e.g., aquifers, depleted oil and gas reservoirs, and coal formations), H_2_ adsorption can theoretically increase storage capacity only if it can be recovered easily. The energy barrier for desorption often leads to a scenario in which not all stored H_2_ can be withdrawn quickly or entirely (i.e., there is hysteresis in adsorption–desorption). In such cases, irreversibly adsorbed H_2_ is considered lost. As discussed in Sections 3 and 5, H_2_ desorption data are scarce: some studies have reported little to no hysteresis,^69,83^ while others have observed significant hysteresis.^20,24,73,74^ Thus, regardless of adsorption capacity, without efficient desorption, adsorbed H_2_ not only fails to contribute to usable storage but also represents a net loss.

From an energy extraction perspective, hysteresis introduces uncertainty in estimating recoverable reserves and can affect the stability and predictability of gas production over time. Even in the absence of hysteresis, not all adsorbed H_2_ can be recovered due to the operational pressure constraints of the storage site. As illustrated schematically in Figure 12, subsurface systems operate within a specific pressure range during the injection and withdrawal cycles. The minimum pressure of the storage site determines the recoverable fraction of adsorbed H_2_, meaning that some loss is inevitable even under ideal conditions.

**Figure 12:**
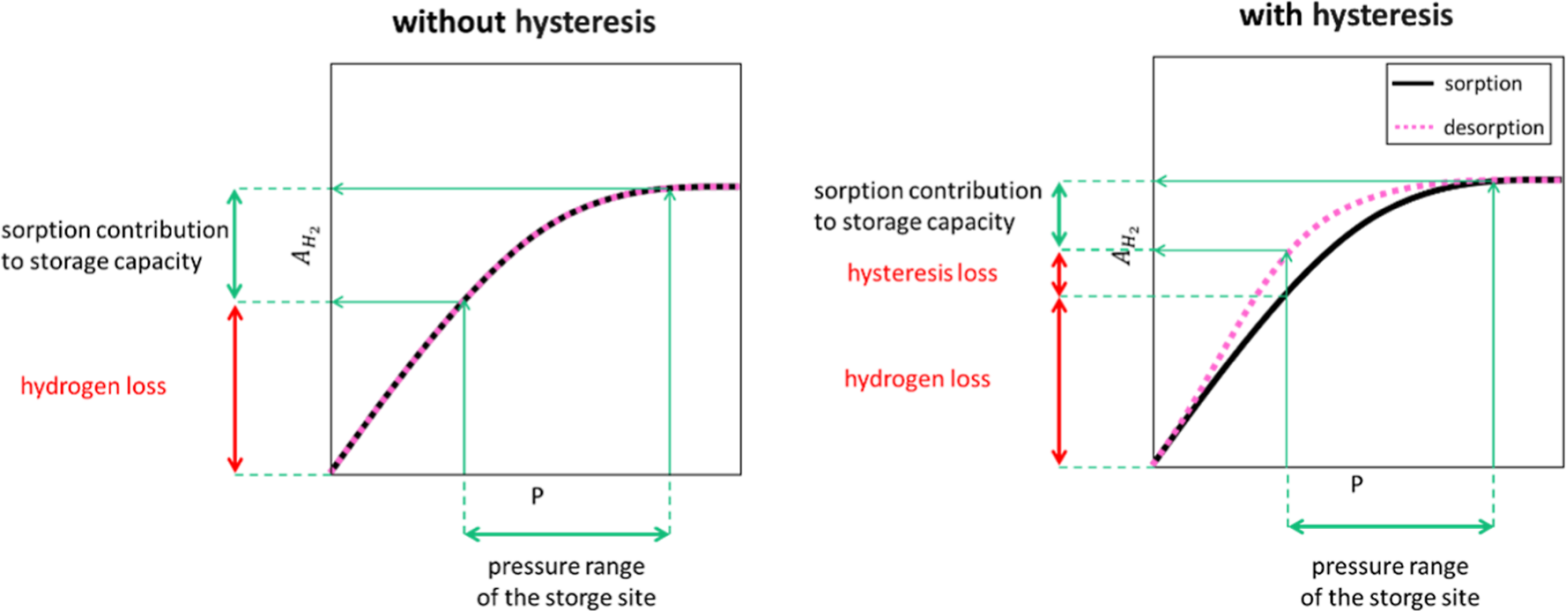
Schematic representation of H_2_ adsorption and desorption behavior in an idealized subsurface storage system: (Left) without hysteresis and (right) with hysteresis. The minimum pressure of the storage site determines the recoverable fraction of adsorbed H_2_, with some loss inevitable even under ideal conditions.

It is important to note that Figure 12 represents an idealized system with minimal or no hysteresis. In practice, more significant hysteresis has been reported in the literature. Furthermore, Figure 12 depicts only the first injection/withdrawal cycle, while the effects of repeated cycling remain poorly understood due to limited data. For instance, Zhang et al. (2024)^73^ conducted 10 cycles of adsorption/desorption tests on MMT and sepiolite. For MMT, no consistent trend was observed across pressure cycles, likely due to pronounced hysteresis effects. In contrast, sepiolite exhibited consistent H_2_ sorption measurements across cycles, indicating minimal hysteresis and more stable sorption properties under repeated pressure variations. However, with repeated cycling, the system would be expected to approach a steady-state equilibrium, in which the amounts of H_2_ sorbed and desorbed per cycle become equal. This occurs because not all sorbed H_2_ is released during desorption, leading to progressively reduced sorption capacity in subsequent cycles until equilibrium is reached. The questions are how many cycles are required to reach this steady state and what role lithology plays in influencing equilibrium behavior.

Under high-pressure conditions and temperatures ranging from 25 to 70 °C, H_2_ sorption leads to an increase in the density of the sorbed phase by up to a factor of 2. This density is comparable to that of gaseous H_2_ sorbed at cryogenic temperatures (77–100 K) and atmospheric pressure.^69^ However, this value remains significantly lower than the density of liquid hydrogen. The elevated density of the sorbed phase substantially increases the volumetric capacity of sorbed H_2_ and, as a result, can amplify both the advantages and the disadvantages of this phenomenon in subsurface systems.

Another important factor to consider in H_2_ storage in porous media is the introduction of H_2_ as a new component to the formation fluid. The composition of the formation fluid varies depending on the repository: it is primarily formation brine in aquifers and a mixture of residual hydrocarbons and brine in depleted oil and gas reservoirs or CBM systems (where CH_4_ is the dominant hydrocarbon). The presence of these fluids means that H_2_ will compete with other molecules (e.g., water, CH_4_, or other gases) for sorption sites.

As discussed previously and evidenced by the data gathered in Section 3, nearly all H_2_ sorption tests on natural porous materials have been conducted on dried powders. However, from the extensive literature on CH_4_ (and CO_2_) adsorption in coal and shale, it is well-established that moisture content significantly reduces gas adsorption capacity—up to the critical (or equilibrium) moisture content. Water molecules decrease gas sorption by adhering to hydrophilic surfaces (primarily carboxylic functional groups) via hydrogen bonding, occupying capillary pores, and aggregating to block pore spaces. Additionally, moisture has a pronounced effect on gas sorption kinetics, decreasing the sorption rate in moist samples. Furthermore, moisture can significantly affect gas desorption due to capillary condensation of the vapor phase. This phenomenon occurs when water vapor condenses in the pore spaces of the rock, blocking access to adsorption sites and hindering the release of sorbed gases. As a result, the reversibility of sorption and the efficiency of gas recovery can be compromised. The effect of moisture on gas sorption has been extensively documented in numerous review articles on CH_4_ and CO_2_ sorption in rocks.^141,144,146,147,150–152,158–160^

H_2_ has been shown to exhibit a significantly lower sorption capacity in shale and coal compared to CH_4_ and CO_2_. For instance, CH_4_ has been documented to have up to 6 times higher sorption capacity, while CO_2_ exhibits 5 to 22 times higher sorption capacity than H_2_.^21,22,29,30,73,88,93^ This stark difference highlights the importance of considering gas competition in UHS, particularly in depleted oil and gas reservoirs and CBM systems, where CH_4_ is often the dominant residual gas. In such systems, the presence of CH_4_ and other gases can significantly reduce the number of available sorption sites for H_2_, potentially limiting its storage capacity.

In nanoporous media, where flow and mass transport are diffusion-dominated, the sorption capacity plays a significant role in determining gas behavior. Strongly sorbing gases, such as CO_2_ in coal systems, exhibit apparent diffusion coefficients up to 2 orders of magnitude smaller than weakly sorbing gases like N_2_ and CH_4_.^161^ For H_2_, which has an even weaker sorption affinity, the apparent diffusion coefficient is expected to be significantly higher than that of CO_2_. However, experimental or simulation data for H_2_ in such systems remain scarce. Recent simulations by Babatunde and Emami-Meybodi (2025)^162^ support this hypothesis. In low-permeability reservoirs, CO_2_ partitions between free and adsorbed phases due to its strong sorption, while H_2_ predominantly occupies the free gas phase. This behavior suggests that H_2_ transport in nanoporous media is less hindered by sorption, resulting in faster diffusion and lower retention compared to CO_2_.

Another critical consideration is the use of powdered samples in sorption experiments. While powdered materials provide a large surface area and (theoretically) facilitate reproducible measurements, they do not accurately represent the natural structure of intact geological formations. In intact rock, minerals are arranged in complex structures, and gas access to adsorption sites is more restricted compared with powdered samples. This means that the sorption capacity measured in laboratory experiments using powders may overestimate the actual adsorption capacity under real-world subsurface conditions. However, the sorption behavior should be quite comparable if it is well normalized to a measured or estimated surface area. Wolff-Boenisch et al. (2023)^23^ proposed a useful approach by reporting H_2_ adsorption capacity as volume adsorbed per specific surface area (μL/m^2^) rather than an absolute value.

Furthermore, many studies focus on pure clay minerals (Section 3.1), but natural rocks typically consist of a diverse range of minerals. The sorption behavior of H_2_ in such systems is the cumulative result of interactions with all of these minerals, as well as their spatial arrangement within the rock matrix. As explained in Section 5, the presence of certain minerals may either enhance or inhibit H_2_ adsorption. Therefore, extrapolating sorption data from pure clay minerals or powdered samples to real-world rocks requires careful consideration of these complexities.

The competitive effects of water and other gases, combined with the significantly lower sorption capacity of H_2_ compared to CH_4_, suggest that H_2_ sorption is not a dominant factor in storage capacity for most porous media, and H_2_ will probably exist in the free phase. However, it cannot be entirely disregarded if the reservoir contains materials with higher sorption capacity (such as organic matter, clay, and diatomite). Its significance depends on factors such as mineralogy and lithology, pressure, and temperature (depth), water saturation, and the presence of competing gases. Overall, while H_2_ sorption is not a primary concern in most UHS systems, site-specific assessments are critical to avoid underestimating potential losses and ensure accurate storage capacity estimates.

### 6.2 Separation of H_2_ and CO_2_ with Coal

Coal exhibits a lower sorption capacity for H_2_ compared to that of CO_2_ and CH_4_, but it has a significantly higher diffusion rate for H_2_. Leveraging these properties, recent studies have proposed a new approach involving the injection of a CO_2_ and H_2_ mixture from SMR (for gray H_2_ production) into coal seams to separate H_2_ from the gas mixture and store CO_2_ within the coalbed simultaneously.^29,30^

For the proposed method of injecting a CO_2_ and H_2_ mixture into coal seams, several critical factors must be considered to assess its feasibility and potential success. These include technical feasibility, operational challenges, and economic viability, among others:

#### 6.2.1 Sorption Selectivity

Under competitive conditions, CO_2_ exhibits twice the sorption capacity of CH_4_ across various coal types and moisture states.^163^ However, moisture significantly reduces overall gas sorption capacity.^163^ Given the presence of pre-existing water in the coal seam, competition of water with CO_2_ for sorption sites potentially reduces the separation efficiency.

#### 6.2.2 Hydrogen Purity and Loss

The injection of CO_2_ can lead to the desorption of the CH_4_—a process that is also employed in enhanced coalbed methane recovery. Therefore, recovered H_2_ may contain residual CO_2_ and CH_4_, requiring subsequent purification steps for industrial use. According to standard ISO 14687, gaseous H_2_ must be at least 98% pure for internal combustion engines and domestic appliances and over 99.9% pure for use in industrial fuels and 99.97% for fuel cells for vehicles.^164^

Additionally, H_2_ can be lost through other processes, such as microbial consumption or conversion,^165,166^ which warrants further investigation.

#### 6.2.3 Coal Injectivity

The permeability and porosity of the coal seam must allow for efficient gas injection and migration.

#### 6.2.4 Coal Swelling/Shrinkage

As was explained earlier in Section 6.1, CO_2_ sorption can induce coal swelling, reducing permeability and injectivity over time.

#### 6.2.5 Long-Term CO_2_ Stability

CO_2_ stored in coal must remain securely sorbed over centuries to avoid leakage, which depends on the coal properties, necessitating thorough analysis and monitoring.

### 6.3 Natural H_2_ Occurrence

The exploration of natural H_2_ has gained significant attention in recent years. Unlike fossil fuel exploration, natural H_2_ requires effective trapping mechanisms to accumulate in the subsurface, as H_2_ is highly mobile and prone to abiotic or biotic consumption. H_2_ sorption on clay minerals may play an important role in both the loss and the retention of H_2_.

#### 6.3.1 Sorption as a Trap or Loss Mechanism

Ellis and Gelman (2024)^167^ proposed that H_2_ sorption on clay minerals could contribute to H_2_ loss at lower temperatures. This hypothesis aligns with the hysteresis effects discussed in Section 6.1, where sorbed H_2_ may become partially irrecoverable. For instance, Truche et al. (2018)^42^ investigated clay-rich rock samples from the Cigar Lake uranium deposit (Canada) using thermal desorption. They found that clay minerals (mainly sudoite) trapped up to 500 ppm of H_2_ (0.25 mmol/g of rock). Over the deposit’s 1.4-billion-year lifespan, they estimated that a maximum 1.34 × 10^4^ tons of H_2_ were produced by water radiolysis, and 4–17% of it was retained in surrounding clays (by sorption).

#### 6.3.2 Seepage Detection

Naturally generated H_2_ may seep into the surface or migrate laterally. Understanding sorption behavior across rock types (e.g., clays vs serpentinites) can improve predictions of migration pathways and accumulation zones, aiding in the identification of viable exploration targets.

#### 6.3.3 Sorption as an Exploration Tool

Sorbed H_2_ may also serve as an indicator of past or present H_2_ flux. Ziemiański and Derkowski (2022)^69^ modeled the maximum sorbed H_2_ capacity in shale and serpentinite systems, assuming 2% microporosity available for sorption. Their results suggest that sorbed H_2_ could account for 20–80% of the total H_2_ in the pore system (depending on the micropore-to-total-pore volume ratio). Thus, detecting sorbed H_2_ in rocks could:

- Reveal historical migration pathways of H_2_.
- Guide models of H_2_ evolution in geological systems.
- Identify lithologies with favorable adsorption properties for targeting natural reservoirs.

#### 6.3.4 Extracting Sorbed Natural H_2_

Extracting sorbed H_2_ would require extensive techno-economic analysis. Insights gained from methane extraction in shale gas and CBM could be highly advantageous in determining the commercial level of H_2_ adsorption.

However, the following properties suggest a potential strategy (Figure 13):

- *Higher CO*_*2*_ *affinity*: CO_2_ sorption capacity on rocks is significantly higher than H_2_.
- *Faster H*_*2*_ *diffusion*: H_2_ migrates more readily than CO_2_.
- *Buoyancy effects*: low density of H_2_ enhances upward migration.

**Figure 13:**
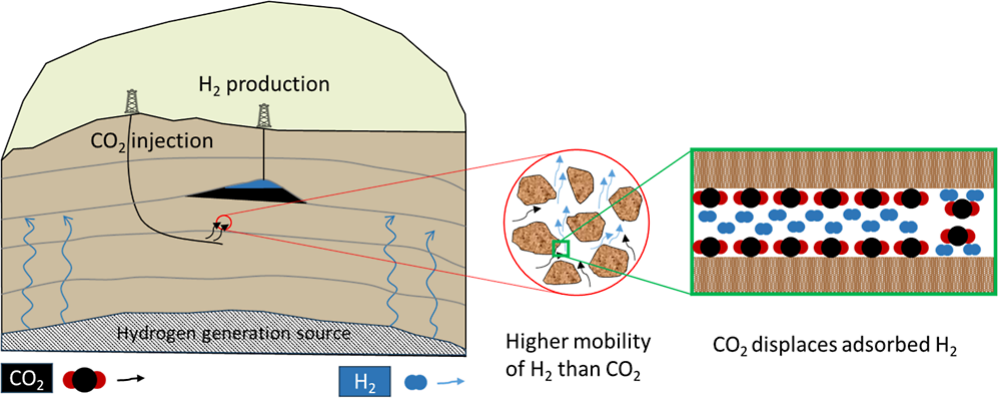
Adsorbed natural H_2_ extraction by CO_2_ injection.

Injecting CO_2_ into such formations could displace adsorbed H_2_, leveraging competitive sorption. The released H_2_ might then accumulate beneath impermeable seals due to buoyancy, provided geological conditions permit safe CO_2_ containment. While promising, this approach requires rigorous feasibility studies to address leakage risks and economic viability.

### 6.4 Waste Storage

H_2_ flow and transport are important in underground waste storage facilities. H_2_ can be released by the degradation of waste materials (such as bitumen and radioactive ash) and the corrosion of metallic containment vessels. As mentioned in Section 1.3, some clay-rich formations are potential candidates for radioactive waste repositories. If gas generation exceeds the diffusive flux through the clay matrix, free gas can accumulate, migrate upward, and pressurize the clay-rich caprock. High gas pressure might even lead to fissures and fractures, compromising the containment system. In such systems, H_2_ sorption is important for decreasing the free gas phase volume and, consequently, delaying pressure buildup.

Increasing the density of sorbed H_2_ at high-pressure^69^ can be particularly beneficial, as it enhances the volumetric storage capacity of the clay matrix. However, several key factors influencing sorption efficiency must be considered:

- **Clay content and mineralogy**: Smectite-rich clays exhibit higher sorption capacity than Illite or kaolinite. Therefore, smectite-to-Illite transformation at larger depths and higher temperatures^168,169^ can reduce sorption capacity, posing a disadvantage.
- **Swelling behavior**: Clay swelling can have both advantageous and disadvantageous effects. On the one hand, swelling can block pores and reduce permeability, limiting gas migration. On the other hand, it may negatively impact the mechanical properties of the rock. These effects must be studied carefully to optimize containment design.
- **Depth:** H_2_ adsorption is strongly dependent on pressure and temperature, which vary with depth. As a result, sorption capacity and behavior may change across different depths, requiring site-specific assessments.

## 7 Challenges and Key Research Issues

Based on this review, we identify the following major scientific and technical challenges, grouped into thematic categories.

### 7.1 Data Scarcity and Measurement Limitations

#### 7.1.1 Limited Hydrogen-Specific Data

Compared to the extensive literature on CH_4_ and CO_2_, experimental data for H_2_ sorption–desorption in natural porous media are scarce. This gap stems from historical research priorities that have favored fossil fuel exploration and CO_2_ storage. More data are needed (across a range of geological materials, pressures, temperatures, and saturations) to better understand this phenomenon and to inform the models.

#### 7.1.2 Sensitivity and Uncertainty of Measurements

H_2_ sorption experiments are prone to many pitfalls compared to other gases^56,69,170^ due to their sensitivity to sorption measurements, high mobility, and diffusive nature of H_2_, instrument design and calibration, and experimental protocols. H_2_ exhibits a low sorption capacity in most natural rocks. Additionally, the sorption capacity of natural porous materials is generally lower than that of advanced-synthetic or modified-natural materials like MOFs, activated carbons, and carbon nanotubes. This intensifies the challenge and makes the results more susceptible to systematic errors and artifacts, especially if the sorption level is very low and close to the detection limits of the experimental setups. Accurate measurements require careful attention to the experimental setup, gas purity, leakage prevention, and instrument calibration. One useful suggestion is performing blank experiments, as suggested by Li et al. (2024),^93^ to overcome the artifacts.

#### 7.1.3 Lack of Standardized Protocols

There is no accepted protocol for H_2_ sorption experiments in geological media. Differences in sample preparation (e.g., particle size and degassing), instrument design, equilibration time, and correction methods make it difficult to compare results across studies. A coordinated effort is needed to establish standardized methodologies, including calibration routines, reporting formats, and validation procedures. Interlaboratory exercises, similar to those performed for other gases on coals and shales,^171–175^ are essential to identify and minimize sources of variability and achieve reproducibility across different laboratories. Measurement methods cannot be standardized until it is understood how to achieve good agreement between data generated in different laboratories.

### 7.2 Desorption, Hysteresis, and Reversibility

#### 7.2.1 Limited Desorption Studies

Most studies to date have focused on sorption isotherms, neglecting desorption hysteresis, kinetics, and reversibility. This gap in the research presents a significant challenge in the predictions of recoverable H_2_ in storage systems. Desorption experiments must be systematically conducted, and data should be reported, regardless of results.

Desorption hysteresis, if present, may also indicate physical or chemical retention mechanisms not taken into account in simple models.

#### 7.2.2 Impact of Cycling and Time

The effect of repeated sorption–desorption cycles on total capacity, pore accessibility, and material structure is poorly understood. As explained in Section 6.1, repeated cycling is expected to gradually drive the system toward a steady–state equilibrium, where the amounts of H_2_ adsorbed and desorbed in each cycle become equal. Main uncertainties are the number of cycles needed to reach this equilibrium and the extent to which lithology influences this behavior.

Moreover, prolonged exposure over time may induce gradual changes in sorption characteristics, potentially due to surface rearrangement, structural compaction, or chemical alteration of the material.

### 7.3 Competitive Sorption and Environmental Effects

#### 7.3.1 Lack of Moisture-Coupled Data

Most studies are conducted under dry conditions, yet subsurface formations often contain water or brine. Moisture reduces the sorption capacity by blocking hydrophilic sites and pores, altering surface energy, or inducing swelling. The influence of water on H_2_ sorption remains unquantified, despite clear evidence of competition in analogous systems.

#### 7.3.2 Multicomponent Gas Interactions

In real subsurface conditions, H_2_ often coexists with other gases such as CH_4_, N_2_, or CO_2_. These species can compete for sorption sites or alter pore accessibility. Multigas experiments are needed to explore these competitive effects under representative conditions, particularly for applications like natural H_2_ extraction via CO_2_ injection (Section 6.2) and H_2_–CO_2_ separation in CBM systems. However, it needs to be mentioned that multicomponent gas adsorption measurements are experimentally challenging because they require accurate determination of composition and equilibrium in each component, with large uncertainties that complicate comparison between models. Binary measurements are at least an order of magnitude more complicated and time-consuming than pure gas measurements.^176^ Additionally, the lack of standardized protocols and reference materials for multicomponent systems perpetuates significant knowledge gaps in understanding gas mixture adsorption in porous materials.^177^

### 7.4 Weak Physical Interactions and Thermodynamic Uncertainties

H_2_ sorption in geological media is governed by physisorption, involving weak van der Waals forces.^73,96^ At typical reservoir temperatures, the adsorption enthalpies are small^69,73^ and may be comparable to thermal energy fluctuations (*K*_B_ *T* ≈ 2.6 kJ/mol at 315 K, where *K*_B_ is the Boltzmann constant). Therefore, assuming reversibility of sorption, sorbed H_2_ may desorb easily with modest changes in temperature or pressure. This raises fundamental questions about the thermodynamic stability and persistence of sorbed H_2_, particularly under variable pressure–temperature conditions typical of geologic formations.

Hence, more detailed thermodynamic consideration is needed to quantify the sensitivity of H_2_ retention to temperature and pressure fluctuations, especially during storage cycling operations.

### 7.5 Mineralogy and Textural Variability

#### 7.5.1 Role of Clay Type and Swelling

Clay minerals are often cited as potential H_2_ sorbents due to their high surface area and interlayer spacing. However, their sorption capacity varies widely depending on the clay type, cation content, and hydration state. The contribution of swelling clays to total sorption in complex rock matrices remains uncertain and context-dependent.

#### 7.5.2 Organic Matter and Coal Variability

Coal and organic-rich shales show higher H_2_ uptake in some cases, attributed to microporosity and functional groups. However, coal rank, maturation, and structural anisotropy introduce significant variability. More controlled experiments on well-characterized coal types are needed to isolate the governing parameters.

### 7.6 Pore Structure, Accessibility, and Anisotropy

#### 7.6.1 Restricted Access and Dead-End Pores

H_2_ uptake may be limited, not only by the total surface area but also by pore accessibility. In rocks with complex or disconnected pore networks, sorption may be confined to near-surface regions. The role of tortuosity, dead-end pores, and surface roughness in limiting H_2_ diffusion and access remains underexplored.

#### 7.6.2 Anisotropy in Sorption Behavior

The pore structure in rocks is often anisotropic, meaning that the size, shape, and connectivity of pores can vary with the direction. As a result, sorption capacity and kinetics may differ depending on the orientation relative to the bedding planes or prevailing stress fields. These anisotropic effects are rarely considered in experimental setups or models, but they could impact field-scale predictions of the sorption and transport processes.

### 7.7 Coupled Geochemical, Mechanical, and Microbial Effect

The coupled effect of H_2_ sorption should also be investigated. For example, sorption-induced swelling or shrinkage and its effect on permeability, geochemomechanical effect of prolonged H_2_ sorption on mineral surfaces, and the possibility of biotic consumption of sorbed H_2_. These changes can alter future sorption behavior or affect reactive transport.

### 7.8 Scale Effects and Upscaling Frameworks

#### 7.8.1 Laboratory vs Field-Scale Discrepancies

Laboratory-scale measurements on powdered samples often overestimate the sorption capacity due to artificially enhanced surface area and fully exposed pore networks. In contrast, intact rock matrices may limit pore accessibility or exhibit different structural behavior. Bridging this gap requires systematic comparisons and representative core-scale testing.

Some studies use the maximum Langmuir capacity (*A*_0_ in eq 1) in their discussions and mix it with the real maximum capacity. This can be misleading, especially if the Langmuir isotherm fails to capture the sorption behavior of the sample and/or measurements are conducted below the plateau pressure, where saturation is not achieved.

#### 7.8.2 Toward Field-Relevant Modeling

Molecular-scale models and adsorption isotherms must be embedded within larger frameworks that account for heterogeneity, stress conditions, fracture networks, and hydrodynamics. The absence of validated upscaling strategies currently limits the practical use of laboratory data in field design.

### 7.9 Economic and Practical Viability

#### 7.9.1 Significance of Sorption

The practical significance of sorption must be evaluated in context. For UHS, sorption may represent a small fraction of the total storage capacity. For natural hydrogen, sorbed gas may act as either a source or a sink, depending on conditions.

#### 7.9.2 Cost–Benefit Analysis

Cost–benefit analysis is required to assess whether sorption-based contributions are meaningful in large-scale systems. For example, even if H_2_ storage in low-permeability media (e.g., shale and coal) is scientifically viable, its economic feasibility for large-scale storage must be assessed relative to other UHS methods. The same goes for the extraction of natural H_2_.

#### 7.9.3 Synergy with CO_2_ Storage

Co-injecting CO_2_ to displace adsorbed H_2_ (Section 6.2) or separate H_2_–CO_2_ mixtures from gray H_2_ production could offer dual advantages. However, leakage risks, chemical interactions, geomechanical instability, and economic feasibility must be considered alongside potential synergies.

### 7.10 In Situ Monitoring and Dynamic Experimental Methods

#### 7.10.1 Limitations of Static Laboratory Experiments

Most H_2_ sorption–desorption studies are conducted in batch or closed systems using static pressures, dry samples, and simplified conditions. These approaches provide initial estimates of capacity but do not reflect the dynamic changes that occur in real geological environments, including fluctuations in pressure, temperature, moisture, and geochemical composition. Static data also fail to capture time-resolved behaviors such as rate-limited uptake, delayed desorption, or hysteresis under cycling.

#### 7.10.2 Lack of In Situ Observation Tools

Real-time monitoring of H_2_ sorption under reservoir-relevant conditions remains rare. There is a lack of instrumentation that can track H_2_ uptake, pressure changes, and surface interactions within rock cores or packed beds under high-pressure, high-temperature, and saturated conditions. The ability to measure sorption while maintaining geochemical and microbial activities is especially limited. This prevents direct observation of coupled effects and long-term evolution of the sorption properties in realistic settings.

#### 7.10.3 Integration with Modeling and Interpretation

New in situ data can support the calibration of dynamic models that account for rate limitations, competitive effects, mineral reactivity, and transient responses. Integrating in situ measurements with sorption models, reactive transport codes, and geomechanical simulations will improve predictions of H_2_ mobility and retention in operational subsurface systems.

## 8 Final Remarks

H_2_ sorption and desorption in natural porous materials remain among the least understood processes relevant to subsurface energy systems. Although frequently cited in the contexts of UHS, natural hydrogen exploration, and radioactive waste disposal, the true significance of these interactions is difficult to quantify without consistent, high-quality data and a robust mechanistic understanding. Current evidence suggests that sorption capacities are very low but also highly variable depending on mineralogy, pore structure, and experimental conditions.

This account highlights several open questions. In particular, the lack of desorption data, the influence of water and coadsorbed gases, and the role of surface-specific interactions remain major scientific challenges. Additional uncertainties stem from the absence of standardized experimental protocols and the fact that H_2_ sorption levels are often very low, approaching the detection limits of current measurement techniques.

## Supplementary Material



## Notes

The effect of the elemental composition (C, H, N, O, S), derived from ultimate analysis, on H_2_ adsorption in coal, the influence of petrophysical properties (vitrinite, liptinite, inertinite, and mineral matter) on H_2_ adsorption in coal, and the model selection criteria for adsorption isotherms in clay, shale, and coal samples (PDF)
