# Supplementary material for: Revisiting Hydrogen Sorption–Desorption in Natural Rocks
Source: Ind Eng Chem Res. 2026 Feb 3;65(6):2984–3026. doi: 10.1021/acs.iecr.5c02081 (PMC12922789; doi:10.1021/acs.iecr.5c02081)
Supplement: Supplementary file 1 [file ie5c02081_si_001.pdf]

Supporting Information

Revisiting Hydrogen Sorption–Desorption in Natural Rocks

Mohammad Masoudi[1,2]*, Ariel G. Meyra[3,4], Mohammad Nooraiepour[2], Mohaddeseh Mousavi Nezhad[5], Aliakbar Hassanpouryouzband[6], and Helge Hellevang[2]

[1] Applied Geoscience Department, SINTEF Industry, 7465 Trondheim, Norway

[2] Department of Geosciences, University of Oslo, P.O. Box 1047 Blindern, 0316 Oslo, Norway

[3] Instituto de Física de Líquidos y Sistemas Bilógicos, Facultad de Ciencias Exactas-UNLP-CONICET, La Plata 1900, Argentina

[4] Centro de Investigación en Mecánica Experimental y Computacional, Berisso, Argentina

[5] Department of Civil and Environmental Engineering, University of Liverpool, Liverpool L69 3GH, U.K.

[6] School of Geosciences, Grant Institute, University of Edinburgh, West Main Road, Edinburgh EH9 3FE, U.K.

* Corresponding author: mohammad.masoudi@sintef.no

This document presents 2 figures and 3 tables.

Figure S.1 illustrates the effect of the elemental composition (C, H, N, O, S), derived from ultimate analysis, on hydrogen adsorption in coal. Figure S.2 shows hydrogen adsorption capacity in coal as a function of maceral composition (vitrinite, liptinite, inertinite).

Tables S.1 to S.3 outline the model selection criteria for adsorption isotherms in clay, shale, and coal samples, respectively.

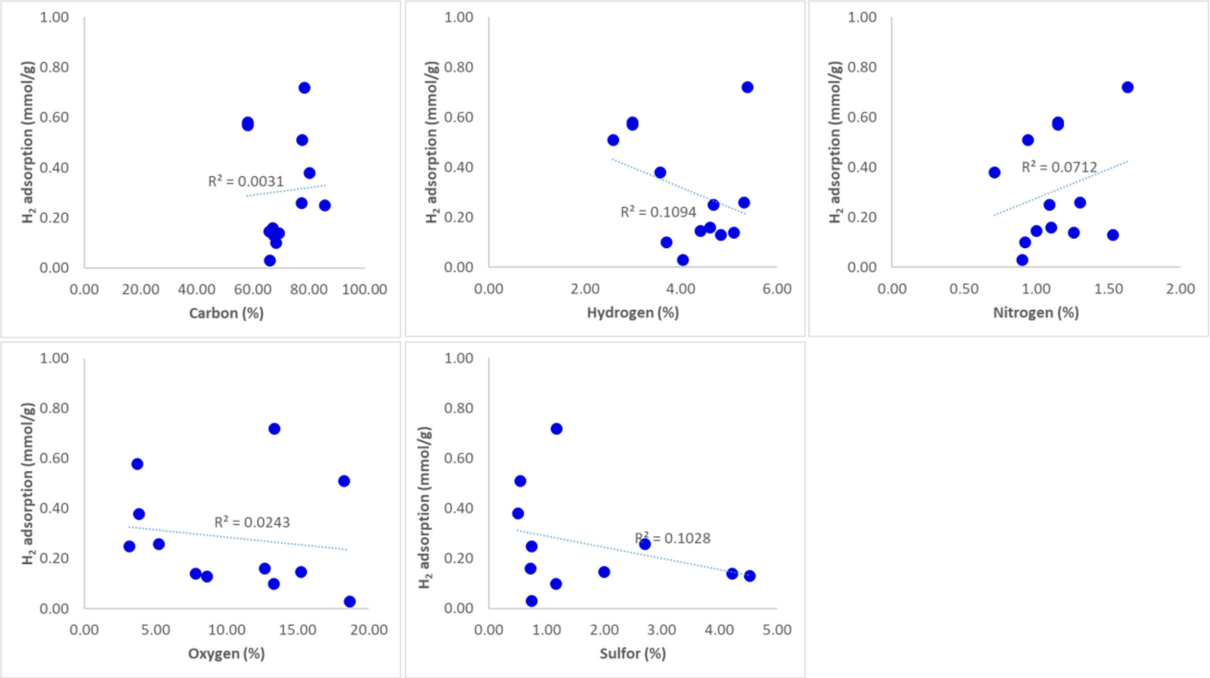

Figure S. 1. Impact of the elemental composition (carbon (C), hydrogen (H), Nitrogen (N), Oxygen (O), Sulfur (S)), derived from ultimate analysis on H_2 adsorption in coal.

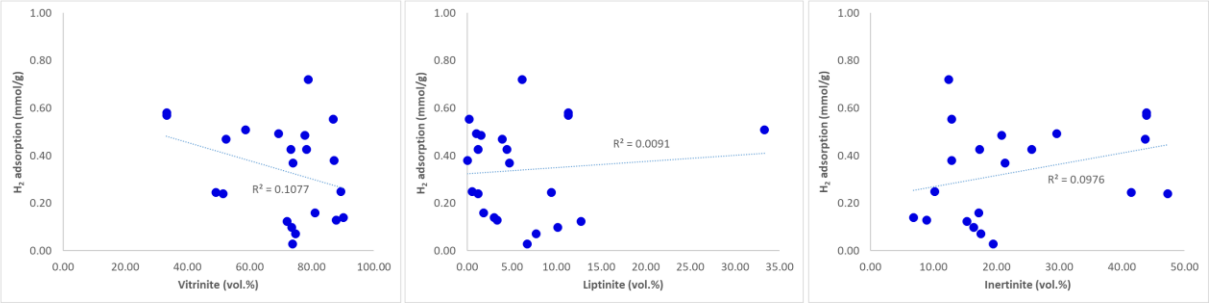

Figure S. 2. Hydrogen adsorption capacity in coal as a function of maceral composition (vitrinite, liptinite, inertinite).

Table S. 1. Model selection criteria for adsorption isotherms in clay samples

Model	Model identification criterion	Ruiz-García et al. (2013)				Bardelli et al. 2014				Mondelli et al. (2015)	
		Thermally treated carbon–montmorillonite (C/MMT)/ 25°C	Thermally treated carbon–sepiolite (C/SEP)/ 25°C	Thermally treated carbon–sepiolite (C/SEP)/ 50°C	Thermally treated carbon–sepiolite (C/SEP)/ 75°C	COXraw/ 28°C	COXraw/ 90°C	COXpur/ 28°C	COXpur/ 90°C	Na-montmorillonite with 0% of structural iron/ 90°C	Na-montmorillonite with 6% of structural iron/ 90°C
Langmuir	BIC	133.3668	155.6965	119.6145	123.1579	159.8987	251.2011	205.4099	250.9054	466.4986	225.8326
	AIC	131.9507	154.1513	118.6447	122.028	157.6278	248.0341	203.1389	248.3892	462.4839	223.5617
	AICC	132.9507	155.0744	119.978	123.228	158.2278	248.3977	203.7389	248.9109	462.7147	224.1617
	RMSE	71.1666	109.096	118.7311	93.6474	28.2116	29.647	75.8765	109.9243	64.5885	118.2829
Redlich-Peterson	BIC	-9.9002	-21.5396	-13.8457	-19.14	-107.4783	-61.7547	10.6077	-14.2126	-71.39	11.1739
	AIC	-12.0244	-23.8573	-15.3004	-20.8349	-110.8848	-66.5053	7.2012	-17.9869	-77.412	7.7674
	AICC	-9.8425	-21.8573	-12.3004	-18.1682	-109.6216	-65.7553	8.4644	-16.8959	-76.9414	9.0306
	RMSE	0.54837	0.39336	0.41168	0.35625	0.07879	0.36531	1.0265	0.63047	0.46847	1.0392
Sips	BIC	-40.5877	-114.9665	-90.7004	-75.0838	-115.1532	-227.6662	-122.4778	-152.9658	-333.2843	-108.2684
	AIC	-42.7118	-117.2842	-92.1551	-76.7786	-118.5597	-232.4167	-125.8843	-156.7401	-339.3063	-111.6749
	AICC	-40.53	-115.2842	-89.1551	-74.112	-117.2965	-231.6667	-124.6212	-155.6492	-338.8357	-110.4117
	RMSE	0.19716	0.021224	0.016743	0.041428	0.066682	0.036467	0.056866	0.043735	0.04332	0.077448
Toth	BIC	-9.6434	-105.4707	-82.5524	-56.6965	-76.3346	-133.9822	-38.4133	-16.9211	-195.3795	-36.9021
	AIC	-11.7676	-107.7885	-84.0071	-58.3914	-79.7411	-138.7327	-41.8198	-20.6953	-201.4015	-40.3085
	AICC	-9.5857	-105.7885	-81.0071	-55.7247	-78.4779	-137.9827	-40.5566	-19.6044	-200.931	-39.0454
	RMSE	0.55308	0.028556	0.023511	0.084029	0.15506	0.13397	0.35361	0.59847	0.15176	0.36542

Model	Model identification criterion	Ziemiański and Derkowski, 2022									
		TMA_Mt_315°C/25°C	TMA_Mt_315°C/50°C	TMA_Mt_315°C/70°C	Cs_Mt_60°C /25°C	Cs_Mt_210°C/25°C	Mg_Mt_40°C/25°C	Mg_Mt_60°C/25°C	Mg_Mt_110°C/25°C	Mg_Mt_210°C/25°C	Mg_Mt_110°C/ 50°C
Langmuir	BIC	95.3816	92.7498	87.6862	75.0229	79.3648	77.3627	70.8001	69.682	71.9752	61.3863
	AIC	94.7764	92.1447	87.081	74.4177	78.569	76.7575	70.4057	69.0768	71.1794	60.9919
	AICC	96.4907	93.859	88.7953	76.132	80.069	78.4718	72.4057	70.7911	72.6794	62.9919
	RMSE	93.5803	82.0422	63.6916	33.8143	29.6511	38.011	40.013	25.8896	21.1917	23.7175
Redlich-Peterson	BIC	-27.8947	-43.8376	-28.1029	-65.1902	-57.9279	-37.0304	-39.7947	-54.7979	-53.66	-38.7823
	AIC	-28.8024	-44.7453	-29.0107	-66.0979	-59.1216	-37.9382	-40.3863	-55.7057	-54.8537	-39.374
	AICC	-24.8024	-40.7453	-25.0107	-62.0979	-55.693	-33.9382	-35.5863	-51.7057	-51.4251	-34.574
	RMSE	0.1755	0.079082	0.17368	0.02719	0.051815	0.11115	0.076	0.045717	0.062908	0.080397
Sips	BIC	-103.2932	-87.1533	-98.7194	-125.1725	-133.267	-116.2213	-105.1772	-115.8285	-104.5552	-87.423
	AIC	-104.201	-88.061	-99.6271	-126.0803	-134.4606	-117.1291	-105.7689	-116.7362	-105.7489	-88.0147
	AICC	-100.201	-84.061	-95.6271	-122.0803	-131.0321	-113.1291	-100.9689	-112.7362	-102.3204	-83.2147
	RMSE	0.0040459	0.0090676	0.0050855	0.0013549	0.0016874	0.0021198	0.0020105	0.0021618	0.006223	0.0053909
Toth	BIC	86.5103	-43.3386	-53.1468	-52.9229	99.5312	80.2594	73.5564	80.2594	-67.7301	-60.6954
	AIC	85.6026	-44.2464	-54.0545	-53.8306	98.3375	79.3516	72.9647	79.3516	-68.9238	-61.2871
	AICC	89.6026	-40.2464	-50.0545	-49.8306	101.7661	83.3516	77.7647	83.3516	-65.4952	-56.4871
	RMSE	53.5239	0.08108	0.049651	0.050211	66.4971	39.1571	41.2753	39.1571	0.033186	0.023798

Model	Model identification criterion	Ziemiański and Derkowski, 2022									
		Mg_Mt_210 °C/ 70°C	Li_Mt_40°C/ 25°C	Li_Mt_60°C / 25°C	Li_Mt_210°C / 25°C	Li_Mt_210°C / 50°C	Li_Mt_210°C / 70°C	Cs_Bd(f)_210°C/ 25°C	Zempleni_210°C/ 25°C	LePUY(f)_210°C/ 25°C	LePUY(f)_210°C/ 50°C
Langmuir	BIC	59.5601	67.3382	67.8468	62.7536	62.0789	60.9105	62.0099	57.0486	75.6124	72.765
	AIC	59.1656	66.9438	67.4523	62.3591	61.6845	60.5161	61.6154	56.4435	75.0073	72.1598
	AICC	61.1656	68.9438	69.4523	64.3591	63.6845	62.5161	63.6154	58.1577	76.7215	73.8741
	RMSE	21.4292	33.0122	33.9581	25.5893	24.6479	23.0989	24.5536	13.7656	34.826	30.2045
Redlich-Peterson	BIC	-45.6116	-40.1806	-40.842	-36.5987	-37.4952	-39.8535	-42.2223	-55.0559	-46.9345	-52.8907
	AIC	-46.2033	-40.7722	-41.4337	-37.1904	-38.0868	-40.4452	-42.814	-55.9637	-47.8422	-53.7985
	AICC	-41.4033	-35.9722	-36.6337	-32.3904	-33.2868	-35.6452	-38.014	-51.9637	-43.8422	-49.7985
	RMSE	0.055013	0.074388	0.071704	0.090766	0.086357	0.075752	0.066411	0.045131	0.067738	0.050291
Sips	BIC	-100.1919	-102.1302	-105.2661	-106.5504	-86.2036	-82.1803	-80.9139	-99.4679	-125.496	-123.5577
	AIC	-100.7836	-102.7219	-105.8578	-107.1421	-86.7952	-82.772	-81.5056	-100.3756	-126.4038	-124.4655
	AICC	-95.9836	-97.9219	-101.0578	-102.3421	-81.9952	-77.972	-76.7056	-96.3756	-122.4038	-120.4655
	RMSE	0.002652	0.0023813	0.0020006	0.0018628	0.0057688	0.0072137	0.0077395	0.0048987	0.0013332	0.0014689
Toth	BIC	-45.7366	-60.0177	-46.3798	-60.6249	-59.9003	-56.9724	-52.1262	77.6967	-53.2072	-56.149
	AIC	-46.3283	-60.6093	-46.9714	-61.2166	-60.492	-57.5641	-52.7178	76.7889	-54.1149	-57.0568
	AICC	-41.5283	-55.8093	-42.1714	-56.4166	-55.692	-52.7641	-47.9178	80.7889	-50.1149	-53.0568
	RMSE	0.054633	0.024711	0.052715	0.023891	0.024872	0.029266	0.038308	34.4478	0.049502	0.042731

Model	Model identification criterion	Ziemiański and Derkowski, 2022	Wolff-Boenisch et al., 2023			Wang et al. 2023					
		LePUY(f)_210°C/ 70°C	Natural pure montmorillonite/-196°C	Natural pure montmorillonite/-78°C	Natural pure montmorillonite/30°C	Palygorskite/0°C	Palygorskite/25°C	Palygorskite/45°C	Palygorskite/65°C	Palygorskite/75°C	Sepiolite/0°C
Langmuir	BIC	69.6661	24.269	43.8589	33.7876	123.4333	124.9705	120.8528	116.6241	114.9861	159.5198
	AIC	69.0609	25.4964	43.9671	34.2041	121.8881	123.4254	119.3077	115.079	113.4409	157.9746
	AICC	70.7752	37.4964	46.9671	38.2041	122.8112	124.3484	120.2307	116.002	114.364	158.8977
	RMSE	25.869	14.6883	17.3709	12.3914	39.8055	41.7643	36.7216	32.1759	30.5703	122.9414
Redlich-Peterson	BIC	-51.2232	-3.9989	-28.9788	-32.8869	-79.8389	-69.2529	-75.4514	-79.9482	-93.7013	-36.9558
	AIC	-52.1309	-2.1578	-28.8166	-32.2622	-82.1567	-71.5707	-77.7691	-82.266	-96.019	-39.2736
	AICC	-48.1309	-20.8166	-20.8166	-20.2622	-80.1567	-69.5707	-75.7691	-80.266	-94.019	-37.2736
	RMSE	0.054664	0.3607	0.083168	0.041233	0.063616	0.088559	0.072964	0.063399	0.04125	0.24297
Sips	BIC	-116.9102	-20.1317	-58.4539	-56.3967	-243.3531	-243.6747	-211.0977	-188.1765	-196.6636	-132.6723
	AIC	-117.8179	-18.2906	-58.2917	-55.772	-245.6709	-245.9925	-213.4155	-190.4943	-198.9814	-134.9901
	AICC	-113.8179	-50.2917	-50.2917	-43.772	-243.6709	-243.9925	-211.4155	-188.4943	-196.9814	-132.9901
	RMSE	0.002048	0.048011	0.01013	0.005813	0.00038406	0.00038022	0.0010523	0.002154	0.0016522	0.012205
Toth	BIC	-55.5082	0.02703	40.6518	43.7369	-61.0716	-105.9783	-89.9546	-90.2758	-89.5992	-31.7912
	AIC	-56.416	1.8681	40.8141	44.3616	-63.3893	-108.2961	-92.2724	-92.5936	-91.917	-34.1089
	AICC	-52.416	48.8141	48.8141	56.3616	-61.3893	-106.2961	-90.2724	-90.5936	-89.917	-32.1089
	RMSE	0.044122	0.59662	12.0218	24.4542	0.11436	0.028107	0.046374	0.045911	0.046892	0.28553

Model	Model identification criterion	Wang et al. 2023									
		Sepiolite 25°C	Sepiolite 45°C	Sepiolite 65°C	Sepiolite 75°C	Montmorillonite/0°C	Montmorillonite/25°C	Montmorillonite/45°C	Montmorillonite/65°C	Montmorillonite/75°C	Chlorite/0°C
Langmuir	BIC	156.9353	158.2489	156.9671	149.4952	102.5197	85.2078	78.6782	58.1105	0.56582	33.0853
	AIC	155.3901	156.7037	155.4219	147.9501	100.9745	83.6626	77.133	56.5654	-0.97935	31.5402
	AICC	156.3132	157.6268	156.345	148.8731	101.8976	84.5857	78.0561	57.4884	-0.056277	32.4632
	RMSE	113.4022	118.1541	113.515	89.8767	20.7066	12.0547	9.8297	5.1689	0.8559	2.3646
Redlich-Peterson	BIC	-66.5015	-80.1266	-87.5435	-143.9652	-86.6601	-89.7119	-96.1873	-105.6983	-172.5516	-137.8682
	AIC	-68.8192	-82.4444	-89.8613	-146.2829	-88.9779	-92.0297	-98.5051	-108.016	-174.8694	-140.1859
	AICC	-66.8192	-80.4444	-87.8613	-144.2829	-86.9779	-90.0297	-96.5051	-106.016	-172.8694	-138.1859
	RMSE	0.096511	0.063046	0.050003	0.0085755	0.051403	0.046727	0.038167	0.028354	0.0035099	0.010375
Sips	BIC	-130.56	-150.7671	-150.2595	-164.2478	-211.4929	-227.483	-227.2686	-199.5836	-206.1408	-216.7743
	AIC	-132.8777	-153.0848	-152.5772	-166.5655	-213.8106	-229.8008	-229.5863	-201.9013	-208.4586	-219.0921
	AICC	-130.8777	-151.0848	-150.5772	-164.5655	-211.8106	-227.8008	-227.5863	-199.9013	-206.4586	-217.0921
	RMSE	0.013037	0.0069334	0.0070443	0.0045498	0.0010394	0.00063064	0.00063488	0.0015081	0.0012287	0.00088129
Toth	BIC	-108.76	-79.9691	-122.4385	-144.649	-120.4723	-124.6477	-140.8839	-38.618	-188.2892	143.4515
	AIC	-111.0778	-82.2869	-124.7562	-146.9667	-122.7901	-126.9654	-143.2017	-40.9357	-190.6069	141.1337
	AICC	-109.0778	-80.2869	-122.7562	-144.9667	-120.7901	-124.9654	-141.2017	-38.9357	-188.6069	143.1337
	RMSE	0.025766	0.063357	0.016804	0.0083942	0.017869	0.015683	0.0094423	0.23068	0.0021464	68.2331

Model	Model identification criterion	Wang et al. 2023				Ghosh et al. 2023				Zhang et al. (2024)	
		Chlorite/25°C	Chlorite/45°C	Chlorite/65°C	Chlorite/75°C	Hydrophilic bentonite (BEN-2)/40°C	Hydrophilic bentonite (BEN-1)/40°C	Acid-treated montmorillonite (MMT-2)/40°C	Acid-treated montmorillonite (MMT-1)/40°C	Sepiolite/0°C	Sepiolite/10°C
Langmuir	BIC	26.9394	33.6471	24.1638	22.7826	25.1258	68.9545	85.5301	76.1079	136.17	132.498
	AIC	25.3942	32.1019	22.6186	21.2375	24.9669	67.8246	84.4002	75.1381	134.6249	130.9528
	AICC	26.3173	33.025	23.5417	22.1605	27.3669	69.0246	85.6002	76.4715	135.5479	131.8759
	RMSE	1.9514	2.4065	1.7893	1.7137	3.7078	11.644	22.028	19.377	59.2655	52.8404
Redlich-Peterson	BIC	-134.1791	-139.0483	-138.475	-198.667	-50.1341	-72.8423	-50.1341	-50.1341	-73.2903	-215.606
	AIC	-136.4969	-141.3661	-140.7928	-200.9848	-50.3724	-74.5372	-50.3724	-50.3724	-75.6081	-217.9238
	AICC	-134.4969	-139.3661	-138.7928	-198.9848	-44.3724	-71.8705	-44.3724	-44.3724	-73.6081	-215.9238
	RMSE	0.011643	0.0099998	0.010181	0.0015519	0.029503	0.045158	0.029503	0.029503	0.078062	0.00091406
Sips	BIC	-207.6353	-226.288	-250.7948	-211.9599	-39.8054	-139.5828	-92.0391	-98.5235	-188.2838	-216.1006
	AIC	-209.953	-228.6058	-253.1126	-214.2776	-40.0438	-141.2777	-93.734	-99.9782	-190.6016	-218.4184
	AICC	-207.953	-226.6058	-251.1126	-212.2776	-34.0438	-138.611	-91.0673	-96.9782	-188.6016	-216.4184
	RMSE	0.0011726	0.00065464	0.00030437	0.0010244	0.056262	0.0034668	0.021581	0.012085	0.0021468	0.00090004
Toth	BIC	150.8753	89.1365	65.2654	-52.8172	40.3644	12.7469	38.9197	64.0609	-105.2366	-197.0008
	AIC	148.5575	86.8187	62.9477	-55.135	40.1261	11.052	37.2249	62.6062	-107.5544	-199.3186
	AICC	150.5575	88.8187	64.9477	-53.135	46.1261	13.7187	39.8916	65.6062	-105.5544	-197.3186
	RMSE	86.0495	12.4982	5.9276	0.14801	8.4392	1.2145	3.3233	10.5761	0.028766	0.0016348

Model	Model identification criterion	Zhang et al. (2024)									
		Sepiolite/20°C	Sepiolite/30°C	Sepiolite/40°C	Sepiolite/50°C	Montmorillonite/0°C	Montmorillonite/10°C	Montmorillonite/20°C	Montmorillonite/30°C	Montmorillonite/40°C	Montmorillonite/50°C
Langmuir	BIC	130.8732	128.3916	124.6967	119.2856	50.9823	56.1912	50.7591	43.3869	43.4278	38.3565
	AIC	129.3281	126.8464	123.1516	117.7404	49.4371	54.646	49.214	41.8417	41.8826	36.8113
	AICC	130.2511	127.7695	124.0746	118.6635	50.3602	55.5691	50.137	42.7648	42.8057	37.7344
	RMSE	50.2244	46.4767	41.4085	34.9664	4.1367	4.868	4.108	3.2627	3.2669	2.7881
Redlich-Peterson	BIC	-61.0538	-113.103	-65.7219	-72.7171	-65.0749	-126.406	-163.8954	-148.6532	-148.0056	-193.6086
	AIC	-63.3716	-115.4207	-68.0397	-75.0349	-67.3927	-128.7237	-166.2132	-150.9709	-150.3234	-195.9264
	AICC	-61.3716	-113.4207	-66.0397	-73.0349	-65.3927	-126.7237	-164.2132	-148.9709	-148.3234	-193.9264
	RMSE	0.11442	0.022496	0.098891	0.079473	0.10091	0.014845	0.0046002	0.0074069	0.0075583	0.0018177
Sips	BIC	-193.4449	-133.2672	-160.5064	-181.9614	-201.817	-223.7269	-187.5592	-208.7128	-201.6549	-197.559
	AIC	-195.7626	-135.5849	-162.8242	-184.2792	-204.1348	-226.0446	-189.877	-211.0306	-203.9727	-199.8768
	AICC	-193.7626	-133.5849	-160.8242	-182.2792	-202.1348	-224.0446	-187.877	-209.0306	-201.9727	-197.8768
	RMSE	0.001827	0.01198	0.0051141	0.0026157	0.0014064	0.00070918	0.0021959	0.0011338	0.0014136	0.0016066
Toth	BIC	-64.23	-70.091	-68.8722	-116.4053	37.9777	-169.1775	-108.8962	-179.5214	-150.9299	-146.3298
	AIC	-66.5478	-72.4088	-71.19	-118.7231	35.66	-171.4953	-111.2139	-181.8392	-153.2477	-148.6476
	AICC	-64.5478	-70.4088	-69.19	-116.7231	37.66	-169.4953	-109.2139	-179.8392	-151.2477	-146.6476
	RMSE	0.10361	0.08627	0.089619	0.020291	2.5266	0.0039002	0.025657	0.0028229	0.0068982	0.0079647

Model	Model identification criterion	Masoudi et al. (2025)				
		Smectite/50	Smectite (Dry)/50	Montmorillonite/50	Montmorillonite_dry/50	Montmorillonite_semi_dry/50
Langmuir	BIC	184	227	173	275	229
	AIC	181	223	171	272	225
	AICC	181	224	171	272	226
	RMSE	12.5	12.8	16.2	34	23
Redlich-Peterson	BIC	-373	-420	-280	-169	-198
	AIC	-377	-425	-285	-174	-203
	AICC	-376	-425	-284	-173	-202
	RMSE	0.004	0.0066	0.007	0.093	0.05
Sips	BIC	-199.2	-497.38	-337.9	-417.9	-385.09
	AIC	-203.9	-502.6	-342.1	-422.8	-389.7
	AICC	-203.1	-502.05	-341.2	-422.1	-388.98
	RMSE	0.049	0.002	0.003	0.0035	0.0035
Toth	BIC	-313.2	-480.5	-297.2	-265	-314.9
	AIC	-317.89	-485.84	-301.4	-269.9	-319.6
	AICC	-317.12	-485.2	-300.49	-269.2	-318.85
	RMSE	0.0097	0.0032	0.0059	0.026	0.0095

Table S. 2. Model selection criteria for adsorption isotherms in shale samples.

Model	Model identification criterion	Abid et al. 2022		Alanazi et al. 2023				Wang et al. 2024			Wang et al. (2024)-Chang 7
		Eagle ford shale 30°C	Aged shale with humic acid 30°C	Jordanian organic-rich source rocks-1 60°C	Jordanian organic-rich source rocks-2 60°C	Jordanian organic-rich source rocks-3 60°C	Jordanian organic-rich source rocks-4 60°C	Longmaxi formation shale 30°C	Longmaxi formation shale 45°C	Longmaxi formation shale 60°C	Sample 412 25°C
Langmuir	BIC	14	37.1	32	35	48	48	46	44	47	69
	AIC	15	37.5	33	36	48	49	46	43	46	67
	AICC	19	41.5	37	40	52	53	48	45	48	68
	RMSE	2.5	16.33	11.45	14	41	43	14.11	9.17	19.8	7.3
Redlich-Peterson	BIC	-44	-27	-18	-17	-17	-18	-24	-41	-36	-106
	AIC	-43	-26	-17	-17	-16	-17	-25	-41	-37	-108
	AICC	-31	-14	-5	-5	-4	-5.8	-19	-36	-32	-106
	RMSE	0.015	0.066	0.1	0.14	0.149	0.137	0.14	0.07	0.089	0.027
Sips	BIC	-46	-34	-18	-18	-18	-18.9	-69	-91	-96	-221
	AIC	-45	-33	-18	-18	-18	-18.34	-69	-91	-97	-224
	AICC	-33	-21	-6.2	-6.27	-6.326	-6.34	-63	-86	-92	-222
	RMSE	0.0137	0.0373	0.13	0.13	0.13	0.13	0.0087	0.0043	0.0032	0.00075
Toth	BIC	-20	-41.9	-9.8	-14.86	-18.97	-18.95	28	28	23.96	77
	AIC	-19	-41.3	-9.19	-14.23	-18.35	-18.33	28	27	23.37	75
	AICC	-7.4	-29.3	2.8	-2.23	-6.35	-6.33	34	32	28.17	77
	RMSE	0.12	0.019	0.28	0.185	0.13	0.131	4.11	3.34	2.62	8.7

Model	Model identification criterion	Wang et al. (2024)-Chang 7								Al-Harbi et al. (2023)	Alanazi et al. (2025)
		Sample 412 45°C	Sample 412 65°C	Sample 427 25°C	Sample 427 45°C	Sample 427 65°C	Sample 413 25°C	Sample 413 45°C	Sample 413 65°C	Midra shale (palygorskite rocks) 50°C	Jo-1 -80°C
	BIC	65	61	77	75	77	79	78	73	30	58
	AIC	64	59	76	74	75	78	76	72	29	58
	AICC	65	60	77	75	76	79	77	73	32	60
Langmuir	RMSE	7.4	5.1	11	10	9.3	10.22	11.33	8.4	5	20.58
	BIC	-98	-140	-88	-92	-120	-112	-89	-102	-42	-82
	AIC	-100	-143	-90	-94	-123	-114	-91	-104	-43	-83
Redlich-Peterson	AICC	-98	-141	-88	-92	-121	-112	-89	-102	-37	-78
	RMSE	0.028	0.012	0.04	0.03	0.017	0.022	0.039	0.031	0.04	0.0069
	BIC	211	-246	-195	-197	-211	-227	-209	-223	-52	-82.7
	AIC	213	-248	-197	-200	-213	-230	-211	-225	-52	-83
	AICC	211	-247	-195	-197	-211	-228	-209	-223	-46	-78
Sips	RMSE	0.0006	0.00055	0.0011	0.001	0.001	0.0006	0.00071	0.00071	0.025	0.0069
	BIC	83	-188	-143	-111	255	78	78	78	-47	-82
	AIC	81	-191	-146	-113	253	76	76	75	-47	-83
	AICC	83	-189	-143	-111	255	78	78	77	-41	-78
Toth	RMSE	12	0.003	0.0062	0.018	2271	9.04	10.4	8.83	0.035	0.007

Model	identification criterion	Alanazi et al. (2025)							Masoudi et al. (2025)		
		Jo-1 0°C	Jo-1 30°C	Jo-1 60°C	Jo-2 -80°C	Jo-2 0°C	Jo-2 30°C	Jo-2 60°C	Hekkingen 50°C	Draupne 50°C	Rurikfjellet 50°C
	BIC	47	37	34	77	53	61	54	223	171	162
	AIC	47	36	34	76	54	61	54	221	169	159
	AICC	50	38	37	78	58	63	57	221	169	159
Langmuir	RMSE	15	6	8	57	64	35	37	33	17	17.8
	BIC	-49	-53	-41	-31	-19	-36	-30	-97	-131	-120
	AIC	-49	-54	-41	-32	-18	-36	-30	-101	-135	-124
Redlich-Peterson	AICC	-43	-49	-33	-27	-6.96	-30	-22	-100	-134	-123
	RMSE	0.03	0.034	0.03	0.11	0.12	0.06	0.07	0.17	0.086	0.089
	BIC	-67	-68	-48	-66	-40	-61	-57	-442	-375	-324
	AIC	-67	-69	-48	-66	-39	-62	-56	-446	-379	-328
	AICC	-61	-64	-40	-62	-27	-56	-48	-445.5	-378	-327
Sips	RMSE	0.01	0.015	0.02	0.017	0.02	0.014	0.011	0.00067	0.0012	0.002
	BIC	-16	10	6.3	-32	-25	-21	22	-297	328	-272
	AIC	-17	10.21	6.5	-32	-24	-21	22	-302	324	-276
	AICC	-11	15	14.5	-27	-12	-15	30	-301	325	-275
Toth	RMSE	0.23	1.26	1.03	0.11	0.079	0.179	3.3	0.0069	243	0.0053

Model	Model identification criterion	Masoudi et al. (2025)						
		Agardfjellet 50°C	Hekkingen_dry 50°C	Draupne_dry 50°C	Rurikfjellet_dry 50°C	Agardfjellet_dry 50°C	Hekkingen'/30°C	Hekkingen 70°C
	BIC	119	129	98	113	135	132	121
	AIC	116	127	94	110	132	129	119
	AICC	116	127	95	110	133	130	120
Langmuir	RMSE	4.9	7.7	3.19	3.4	10.87	12.35	15.66
	BIC	-341	-244	-492	-466	-253	-219	-193
	AIC	-346	-249	-497	-471	-257	-222	-196
Redlich-Peterson	AICC	-45	-248	-497	-471	-256	-221	-195
	RMSE	0.0065	0.014	0.0015	0.0038	0.00766	0.01	0.0079
	BIC	-465	-374	-507	-556	-306	-341	-305
	AIC	-470	-378	-512	-562	-310	-345	-308
	AICC	-469	-377	-511	-561	-309	-344	-307
Sips	RMSE	0.0011	0.0016	0.0012	0.0013	0.0028	0.00088	0.00055
	BIC	-323	-230	454	-502	231	216	186
	AIC	-328	-235	449	-507	227	213	183
	AICC	-327	-234	450	-507	228	214	185
Toth	RMSE	0.0084	0.017	294	0.0025	60.5277	62	68.63

Table S. 3. Model selection criteria for adsorption isotherms in coal samples.

Model	Model identification criterion	Yang and Saunders (1985)		Iglauer et al. (2021)			Abid et al. (2022)				Liu and Liu (2023)
		Pittsburgh bituminous coal (PB coal) 22°C	Pittsburgh bituminous coal (PB coal) 105°C	Sub-bituminous coal 30°C	Sub-bituminous coal 45°C	Sub-bituminous coal 60°C	Sub-bituminous coal 30°C	Sub-bituminous coal-1 30°C	Sub-bituminous coal-2 30°C	Sub-bituminous coal-3 30°C	Lignite type A (LigA) from Beulah Seam, USA 30°C
	**BIC**	57.6135	44.9627	47.2473	39.1364	36.0268	28.7614	39.3206	45.898	47.4192	20.1251
	**AIC**	58.0299	45.7438	48.0284	40.3638	37.2542	29.9888	40.5481	47.1255	48.2004	20.5416
	**AICC**	62.0299	51.7438	54.0284	52.3638	49.2542	41.9888	52.5481	59.1255	54.2004	24.5416
**Langmuir**	**RMSE**	90.242	64.9994	81.6825	94.2056	63.8653	25.7543	96.4	219.3528	83.0993	3.9688
	**BIC**	-40.0516	-33.6893	-15.7954	-12.0778	-11.6947	-22.775	-11.0104	-12.6163	-16.347	-53.0624
	**AIC**	-39.4269	-32.5176	-14.6238	-10.2367	-9.8536	-20.9339	-9.1693	-10.7752	-15.1753	-52.4377
	**AICC**	-27.4269	-8.5176	9.3762	Inf	Inf	Inf	Inf	Inf	8.8247	-40.4377
**Redlich-Peterson**	**RMSE**	0.022696	0.021242	0.12715	0.13139	0.13784	0.034502	0.15014	0.12284	0.12033	0.0076748
	**BIC**	-69.7601	-45.7019	-35.5601	-32.2244	-31.7896	-13.2494	-114.4017	-121.9383	-35.8479	-59.7032
	**AIC**	-69.1353	-44.5302	-34.3884	-30.3833	-29.9484	-11.4083	-125.2317	-120.0972	-34.6762	-59.0785
	**AICC**	-57.1353	-20.5302	-10.3884	Inf	Inf	Inf	Inf	Inf	-10.6762	-47.0785
**Sips**	**RMSE**	0.0019088	0.00639	0.017618	0.010589	0.011181	0.11349	3.66E-07	1.43E-07	0.017118	0.004413
	**BIC**	-31.5792	-29.5424	-23.7639	-18.0571	32.6148	27.2306	-12.6711	7.334	-27.4886	68.0296
	**AIC**	-30.9544	-28.3707	-22.5922	-16.216	34.4559	29.0717	-10.83	9.1752	-26.317	68.6543
	**AICC**	-18.9544	-4.3707	1.4078	AICc: Inf	AICc: Inf	Inf	Inf	Inf	-2.317	80.6543
**Toth**	**RMSE**	0.045981	0.032159	0.057314	0.062225	35.0575	17.885	0.122	1.4872	0.03949	185.155

Model	Model identification criterion	Liu and Liu (2023)							Arif et al. (2022)		
		Sub-bituminous type B (SubB)/ 30 from Rosebud Seam 30°C	Sub-bituminous type A (SubA) from Deadman Seam 30°C	High-volatile type C bituminous (HvCb) from Illinois #6 Seam 30°C	High-volatile type B bituminous (HvBb) from Kentucky #9 Seam 30°C	Low-volatile bituminous (LvB) from Pocahontas #3 Seam 30°C	Fresh semi-anthracite (SemiAn) from Goodspring #3 Seam 30°C	Anthracite (An) from Lykens #2 Seam 30°C	Anthracite coal from USA 30°C	Anthracite coal from USA 45°C	Anthracite coal from USA 60°C
	**BIC**	48.5269	65.527	56.4303	52.2153	69.2446	66.1262	68.6635	39.3848	37.1148	29.5271
	**AIC**	48.6351	65.3681	56.5385	52.0565	69.0857	65.9673	68.5046	40.6122	38.3422	30.7545
	**AICC**	51.6351	67.7681	59.5385	54.4565	71.4857	68.3673	70.9046	52.6122	50.3422	42.7545
**Langmuir**	**RMSE**	24.2453	46.3169	42.6381	20.1565	58.4315	48.0843	56.3474	97.1759	73.1695	28.341
	**BIC**	-68.0675	-55.3181	-50.2002	-70.42	-64.3573	-41.7429	-50.2457	-6.9883	-21.7421	-40.999
	**AIC**	-67.9052	-55.5564	-50.0379	-70.6583	-64.5956	-41.9812	-50.484	-5.1472	-19.9009	-39.1579
	**AICC**	-59.9052	-49.5564	-42.0379	-64.6583	-58.5956	-35.9812	-44.484	Inf	Inf	Inf
**Redlich-Peterson**	**RMSE**	0.0050978	0.021338	0.018266	0.0083029	0.012128	0.049845	0.029298	0.24823	0.039257	0.0035361
	**BIC**	-59.0335	-64.4547	-64.7661	-70.9274	-81.7902	-91.4486	-91.2649	-118.2867	-107.0086	-56.677
	**AIC**	-58.8712	-64.693	-64.6039	-71.1657	-82.0285	-91.6869	-91.5032	-116.4456	-105.1675	-54.8359
	**AICC**	-50.8712	-58.693	-56.6039	-65.1657	-76.0285	-85.6869	-85.5032	Inf	Inf	Inf
**Sips**	**RMSE**	0.0097191	0.012055	0.0064535	0.0080438	0.0040795	0.0022307	0.0022565	2.25E-07	9.23E-07	0.00049821
	**BIC**	-41.4579	-43.0601	-37.3823	-50.7434	-34.4427	-42.7003	-68.4273	-14.1986	-19.6948	29.2252
	**AIC**	-41.2957	-43.2984	-37.22	-50.9817	-34.6811	-42.9386	-68.6656	-12.3575	-17.8537	31.0664
	**AICC**	-33.2957	-37.2984	-29.22	-44.9817	-28.6811	-36.9386	-62.6656	Inf	Inf	Inf
**Toth**	**RMSE**	0.034107	0.045906	0.045632	0.0284	0.078664	0.046951	0.0094042	0.10079	0.050706	22.9495

Model	Model identification criterion	Arif et al. (2022)						Li et al. (2024)			Liu and Liu (2024)
		Bituminous coal from USA 30°C	Bituminous coal from USA 45°C	Bituminous coal from USA 60°C	Sub-bituminous coal from Australia 30°C	Sub-bituminous coal from Australia 45°C	Sub-bituminous coal from Australia 60°C	Cretaceous Cameo coal 30°C	Cretaceous Cameo coal 45°C	Cretaceous Cameo coal 60°C	Anthracite coal specimen, Williamstown Pennsylvania (Dry basis)
Langmuir	BIC	38.5845	38.0086	42.4275	41.5097	31.269	23.5824	199.0099	195.3153	190.3352	72.9454
	AIC	39.8119	39.236	43.6549	42.7371	32.4964	24.8098	196.9208	193.2262	188.2461	72.551
	AICC	51.8119	51.236	55.6549	54.7371	44.4964	36.8098	197.5875	193.8929	188.9128	74.551
	RMSE	87.9257	81.8187	142.1473	126.7402	35.2353	13.4803	98.8252	90.5033	80.3838	45.0776
Redlich-Peterson	BIC	-21.7489	-15.8366	-18.0795	-19.9044	-14.864	-16.9714	-128.1455	-142.0801	-133.1702	-41.1488
	AIC	-19.9077	-13.9955	-16.2384	-18.0633	-13.0229	-15.1303	-131.279	-145.2137	-136.3038	-41.7405
	AICC	Inf	Inf	Inf	Inf	Inf	Inf	-129.8673	-143.8019	-134.892	-36.9405
	RMSE	0.039224	0.082131	0.062051	0.049395	0.092749	0.07127	0.038062	0.027315	0.03377	0.070492
Sips	BIC	-101.6096	-28.8275	-25.0893	-107.1189	-31.2816	-33.7645	-333.9084	-268.6011	-247.5083	-104.672
	AIC	-99.7684	-26.9864	-23.2482	-105.2778	-29.4405	-31.9234	-337.042	-271.7346	-250.6418	-105.2637
	AICC	Inf	Inf	Inf	Inf	Inf	Inf	-335.6302	-270.3229	-249.2301	-100.4637
	RMSE	1.81E-06	0.016191	0.025835	9.10E-07	0.011914	0.008735	0.00028368	0.0013432	0.0022194	0.0020677
Toth	BIC	-12.082	-12.4436	-17.1526	-12.0847	-3.6878	24.0859	-83.691	-91.3989	-120.2345	-42.8715
	AIC	-10.2409	-10.6025	-15.3115	-10.2436	-1.8467	25.927	-86.8246	-94.5324	-123.368	-43.4632
	AICC	Inf	Inf	Inf	Inf	Inf	Inf	-85.4128	-93.1207	-121.9563	-38.6632
	RMSE	0.13132	0.12552	0.069674	0.13128	0.375	12.0718	0.10969	0.091298	0.04595	0.064059
